# The role and impact of the IL-6 mediated JAK2-STAT1/3 signaling pathway in the pathogenesis of gout

**DOI:** 10.3389/fphar.2025.1480844

**Published:** 2025-03-18

**Authors:** Zeng Zhang, Peng Wang, Tianyi Lei, Jianwei Guo, Yi Jiang, Yanhui Li, Jianxiong Zheng, Shunbing Wang, Haimuzi Xu, Guilin Jian, Quanbo Zhang, Yufeng Qing

**Affiliations:** ^1^ Hyperuricaemia and Gout Research Centre, Affiliated Hospital of North Sichuan Medical College, Nanchong, Sichuan, China; ^2^ Department of Geriatrics, Affiliated Hospital of North Sichuan Medical College, Nanchong, Sichuan, China; ^3^ Department of Rheumatology and Immunology, Affiliated Hospital of North Sichuan Medical College, Nanchong, Sichuan, China; ^4^ The Third People’s Hospital of Suining, Suining, Sichuan, China

**Keywords:** gout, IL-6, JAK2, STAT1, stat3, inflammation

## Abstract

**Background:**

Interleukin-6 (IL-6) is a pleiotropic cytokine, with specific effects depending on the immune microenvironment. Extensive research has confirmed the pathological roles of the IL-6/JAK2/STAT1/3 signaling pathway in inflammation, autoimmunity, and cancer, as well as its involvement in the pathogenesis of various rheumatic diseases. However, the role and impact of IL-6 as an upstream regulator of the JAK2-STAT1/3 pathway in gout have seldom been reported. This study explores the influence and role of upstream IL-6 in regulating the JAK2-STAT1/3 signaling pathway on gout inflammation, offering new insights for targeted therapeutic interventions and drug development in gout management.

**Methods and Results:**

Clinical data and peripheral blood specimens were collected from gout patients and healthy individuals. In vitro and in vivo models of acute gout inflammation were established by stimulating PBMCs, THP-1 cells, and mice with MSU crystals. IL-6 expression was manipulated using IL-6 agonists and IL-6 knockout (KO) mouse technology to investigate the role and impact of the IL-6-mediated JAK2-STAT1/3 signaling pathway in gout models. RT-qPCR, WB, and ELISA were utilized to assess gene and protein expression levels. Paw swelling in mice was measured using a caliper gauge, while HE and IHC staining were conducted to evaluate the inflammatory status of mouse paw pad synovial tissues and detect the positive expression of relevant proteins. Serum IL-6 protein expression levels were significantly elevated in patients with gouty arthritis (GA) compared to healthy individuals, with multifactor logistic regression revealing an odds ratio (OR) of 2.175 for IL-6. In GA patients, mRNA expression of IL-6, JAK2, STAT1/3, and IL-1β was notably lower in the gout group compared to the healthy control (HC) group. Moreover, IL-6, JAK2, STAT1/3, p-JAK2, p-STAT1/3, and IL-1β proteins were markedly higher in the acute gout (AG) group compared to the intercritical gout (IG) and HC groups. Within the IG group, IL-6, JAK2, STAT3, and IL-1β proteins were significantly elevated compared to the HC group, whereas STAT1, p-JAK2, and p-STAT1/3 proteins were significantly lower. The expression of IL-6 protein and JAK2 mRNA showed positive correlations with certain inflammatory markers. In the 2h human blood in vitro gout inflammation model, expressions of IL-1β, IL-6, JAK2 mRNA, and IL-1β, IL-6, JAK2, STAT1/3, p-JAK2, p-STAT1/3 proteins were significantly higher compared to both the blank control and PBS-negative control groups. In the acute gout THP-1 cell model, The 6-hour model group showed significantly higher levels of IL-1β, IL-6, JAK2, STAT1/3 mRNA, and corresponding proteins, including their phosphorylated forms, compared to the blank control group. Additionally, treatment with an IL-6 agonist further increased these expression levels compared to the untreated model group. In the acute gout mouse model, IL-6 KO mice exhibited significantly reduced footpad swelling and swelling index compared to wild-type (WT) mice. HE staining revealed decreased inflammatory cell infiltration in IL-6 KO mice. Furthermore, Compared to 12-hour gout model WT mice, IL-1β, IL-6, JAK2, STAT1/3 mRNA, protein expression, and phosphorylated protein levels were notably decreased in IL-6 KO mice. IHC staining showed reduced positive expression of p-JAK2 and p-STAT1/3 in IL-6 KO mice. At the 24-hour mark, IL-6 mRNA and protein expression levels did not differ significantly between IL-6 KO and WT mice; however, IL-1β mRNA and protein expression, as well as JAK2 and STAT3 mRNA expression, were reduced in IL-6 KO mice, while STAT1 mRNA expression remained similar.

**Conclusion:**

IL-6 emerges as a potential risk factor for acute gout attacks, with its involvement in the JAK2-STAT1/3 signaling pathway contributing to the inflammation and pathogenesis process of acute gout through positive feedback mechanisms.

## Introduction

Gouty arthritis (GA) is an inflammatory disorder caused by disrupted purine metabolism, leading to abnormal deposition of monosodium urate (MSU) crystals in joints and surrounding tissues. It presents with joint redness, swelling, heat, pain, and functional impairment, potentially resulting in severe complications such as joint disability, uric acid nephropathy, and renal failure (He et al., 2023). In China, the number of gout patients was 16.2 million in 2019, with an age-standardized prevalence rate (ASPR) of 12.3% in males and 3.9% in females. The ASPR of gout has been increasing from 1990 to 2019, and projections indicate it will reach 11.7% in males and 4.0% in females by 2029, posing a significant burden on society and healthcare systems (Zhu et al., 2022). The inflammatory response in gout involves various cytokines such as IL-6, IL-1β, and TNF-α, pivotal in the amplification cascade of inflammation. Overproduction of these cytokines can lead to systemic manifestations like hemodynamic instability and metabolic disorders, contributing to pain syndromes (Pinto et al., 2021). Research (Zhang et al., 2021) has identified that both acute and chronic inflammation, alongside immune dysregulation, are significant factors in the pathogenesis of gout. Toll-like receptors and the NLRP3 inflammasome have been highlighted as crucial mechanisms underlying gout (Zhang et al., 2021), yet the *in vivo* self-regulation mechanism remains unclear. The JAK2-STAT1/3 signaling pathway, modulated by IL-6, is an intracellular pathway crucial for immune regulation. IL-6 mediates inflammation occurrence and progression through this pathway. Increasing evidence indicates (Hu et al., 2021) that dysregulation of the JAK2-STAT1/3 pathway is linked to various cancers, autoimmune disorders, and inflammatory conditions, and it plays a critical role in vivo self-regulation mechanisms.

The primary treatment goals for acute gout focus on alleviating inflammation, managing pain, and relieving symptoms. Common therapeutic approaches include non-steroidal anti-inflammatory drugs (NSAIDs), glucocorticoids, colchicine, and IL-1 antagonists to counteract acute inflammation. Chronic management of gout primarily revolves around lowering uric acid levels, often using medications such as allopurinol, to prevent recurrence and disease progression. However, these conventional therapies frequently lead to varying degrees of adverse reactions and complications (Keysser, 2020). In recent years, IL-6 has emerged as a pivotal factor in several inflammatory diseases (Kaneko and Takeuchi, 2021), including rheumatoid arthritis, systemic juvenile idiopathic arthritis, and vasculitis. The efficacy of tocilizumab, the first approved anti-IL-6 biologic, has been validated in treating these conditions (Kaneko and Takeuchi, 2021). Notably, studies by Mokuda et al. (2014) and Pinto et al. (2013) have reported the effectiveness of tocilizumab in treating a resistant case of severe tophaceous gout in a female patient and a severe tophaceous gout case in a male patient. Furthermore, research suggests that tocilizumab or baricitinib can inhibit IL-6 or its mediated JAK/STAT signaling pathway-induced inflammation in MSU-induced neutrophils (Temmoku et al., 2021). The JAK/STAT pathway functions as a central signaling hub for numerous inflammatory cytokines and plays a crucial role in the pathogenesis and progression of rheumatic diseases. Consequently, an increasing number of JAK inhibitors are being utilized in the treatment of rheumatic immune disorders (Tzeng et al., 2021). Currently, there is significant progress in clinical trials involving candidate molecules targeting the IL-6 and IL-6 signaling pathways across various diseases (Rose-John et al., 2023; Yao et al., 2014). Advancing biological understanding of the IL-6 and JAK/STAT signaling pathways enables clinical practitioners to better grasp how these insights influence the treatment strategies for autoimmune and inflammatory conditions. This study employs clinical analysis and establishes both *in vitro* and *in vivo* models of acute gouty arthritis (AGA) to investigate the role and impact of upstream IL-6 regulation of the JAK2-STAT1/3 signaling pathway on gout inflammation, while exploring potential underlying mechanisms. The objective is to enhance understanding of the self-regulatory mechanisms in gout and to offer new perspectives or theoretical foundations for clinical treatment strategies.

## Materials and methods

### Preparation of MSU crystals

One Gram of uric acid was dissolved in 200 mL of boiling water containing 6 mL of 1N NaOH. Hydrochloric acid was added to adjust the pH of the solution to 7.2. The solution was cooled with stirring at room temperature and then incubated overnight at 4°C. The precipitate was separated from the solution by filtration and dried under low temperature conditions. The crystals were weighed under sterile conditions and suspended in PBS at concentrations of 80 mg/mL and 25 mg/mL.

### Patient samples and clinical data

A total of 111 cases were included in this study, comprising 55 cases of acute-phase gout (AG group) and 56 cases of intermittent gout (IG group), all male patients attending the Department of Rheumatology and Immunology at the Affiliated Hospital of Chuanbei Medical College from January 2023 to June 2023. All patients met the diagnostic criteria for gout established by ACR/EULAR in 2015, and complete clinical data were available. During the same period, blood specimens and data were collected from 57 male individuals undergoing health check-ups (HC group) in the hospital’s medical examination department. Peripheral blood mononuclear cells (PBMCs) and serum were obtained from these participants. Informed consent was obtained from all participants, and the study was approved by the Medical Ethics Committee (approval number: 2022ER376-1).

### Human blood *in vitro* function experiment

Peripheral venous blood (32 mL each from 5 cases of HC) was collected and divided into eight groups. PBMCs were isolated using lymphocyte isolation solution in an ultra-clean environment. The cells were cultured in RPMI-1640 medium supplemented with 10% fetal bovine serum at a density of 5 × 10^5^ cells/mL in 6-well plates. MSU crystals at a concentration of 100 μg/mL were used to stimulate cells at time points of 0, 1, 2, 4, 6, 8, 10, and 12 h. Incubation was conducted under standard conditions in a CO2 incubator (5 mL/L CO2). The concentrations of IL-1β and IL-6 proteins in plasma were measured using ELISA. Additionally, three tubes (4 mL each) of peripheral venous blood from 19 HC cases were collected, and PBMCs were isolated and cultured as described above. These cells were stimulated with 100 μg/mL MSU crystals for 2 h. A blank control group and a negative control group (PBS stimulation for 2 h) were included. Supernatants and cells were collected after treatment.

### THP-1 cell experiments

Human myeloid leukemia mononuclear cells (THP1) were obtained from the cell bank of the Chinese Academy of Sciences. Cells were cultured in RPMI-1640 medium supplemented with 10% fetal bovine serum (ThermoFisher Scientific, USA) and 1% penicillin-streptomycin, and maintained in a humidified incubator at 37°C with 5% CO2. THP1 cells were differentiated using 100 ng/mL phorbol ester (Sigma, USA) for 48 h. Subsequently, cells were stimulated with 100 μg/mL MSU for 0, 3, 6, 9, and 12 h and maintained in a 37°C incubator with 5% CO2. For specific experiments, cells were stimulated with 100 μg/mL MSU for 6 h alone, or with 100 μg/mL MSU in combination with IL-6R alpha [MedChemExpress (MCE) Catalogue No: HY-P7223, USA] for a total of 6 h. A blank control was included for comparison. Supernatants and cells were collected after treatment.

### Animal experiments

Heterozygous IL-6 knockout (IL-6+/−) mice were generated by breeding IL-6+/+ and IL-6−/− mice, followed by genotyping of the offspring within the same litter to identify IL-6+/+, IL-6+/−, and IL-6−/− genotypes. The IL-6 KO mice [obtained from the Max Planck Institute for Immunobiology, Freiburg, Germany; B6; 129S2 (Stock No. 002254)] and wild-type (WT) mice [purchased from SPF (Beijing) Biotechnology Co., Ltd.; SCXK (Jing) 2019–0,010] weighed 20–25 g. All mice were housed in pathogen-free facilities at the North Sichuan Medical College Animal Center under a 12-h light/dark cycle, with a relative humidity of 50%–70% and a temperature of 24°C ± 2°C. All animal handling and experimental procedures complied with the guidelines of the Institutional Animal Care and Use Committee (IACUC), and the study was approved by the Animal Ethics Committee of North Sichuan Medical College [Approval No. NSMC-IACUC-2023–082]. Each group, consisting of 6–8 mice, received an injection of 150 μL MSU (80 mg/mL) into the synovial space of the right foot pad of WT and IL-6 KO mice. The swelling index was calculated as (thickness of footpad injected with MSU - initial footpad thickness)/initial footpad thickness, with a ratio >0.15 indicating inflammation. Footpad thickness was measured at specified time points using electronic calipers. Subsequently, mice were anesthetized and euthanized in batches, and footpad tissues were processed for total RNA extraction using Trizol, total protein extraction *via* RIPA homogenization, and supernatant collection for cytokine analysis. Synovial tissues were fixed in 4% paraformaldehyde. Sections were subjected to Hematoxylin-eosin (HE) staining for histological analysis of inflammatory cell infiltration under a light microscope (×40 objective lens). Immunohistochemistry (IHC) staining was performed to observe p-JAK2 and p-STAT1/3 positive areas, following the kit instructions and high-pressure antigen retrieval method. Primary antibodies included rabbit anti-mouse p-JAK2, p-STAT1, and p-STAT3 antibodies (diluted 1:200). Slides were examined at ×400 magnification using a BA400Digital microscope and analyzed with the Halo 101-WL-HALO-1 Data Image Acquisition System.

### Primer design and synthesis

The primers for human and mouse β-Actin, GAPDH, IL-1β, IL-6, JAK2, STAT1, and STAT3 genes were designed based on their gene sequences obtained from PubMed Gene. The primers were synthesized by Shanghai Shenggong Bioengineering Company, and the gene sequences are detailed in Table 1 and Table 2.

**TABLE 1:** Primer sequences for human internal reference and target genes.

Gene name	Forward primer(5′-3′)	Reverse primer(5′-3′)
β-Actin	5′GAG​CTA​CGA​GCT​GCC​TGA​CG3′	5′GTA​GTT​TCG​TGG​ATG​CCA​CAG3′
GAPDH	5′ATC​GCC​CAC​TTG​ATT​TTG​G3′	5′GGA​TTT​GGT​CGT​ATT​GGG​CG3′
IL-1β	5′ATG​ATG​GCT​TAT​TAC​AGT​GGC​AA3′	5′GTC​GGA​GAT​TCG​TAG​CTG​GA3′
IL-6	5′ACT​CAC​CTC​TTC​AGA​ACG​AAT​TG3′	5′CCA​TCT​TTG​GAA​GGT​TCA​GGT​TG3′
JAK2	5′TCT​GGG​GAG​TAT​GTT​GCA​GAA3′	5′AGA​CAT​GGT​TGG​GTG​GAT​ACC3′
STAT1	5′ATC​AGG​CTC​AGT​CGG​GGA​ATA3′	5′TGG​TCT​CGT​GTT​CTC​TGT​TCT3′
STAT3	5′ACC​AGC​AGT​ATA​GCC​GCT​TC3′	5′GCC​ACA​ATC​CGG​GCA​ATC​T3′

**TABLE 2:** Primer sequences for mouse internal reference and target genes.

Gene name	Forward primer(5′-3′)	Reverse primer(5′-3′)
β-Actin	5′GAG​CTA​CGA​GCT​GCC​TGA​CG3′	5′GTA​GTT​TCG​TGG​ATG​CCA​CAG3′
GAPDH	5′AGG​TCG​GTG​TGA​ACG​GAT​TTG3′	5′GGG​GTC​GTT​GAT​GGC​AAC​A3′
IL-1β	5′GAA​ATG​CCA​CCT​TTT​GAC​AGT​G3′	5′TGG​ATG​CTC​TCA​TCA​GGA​CAG3′
IL-6	5′TCT​ATA​CCA​CTT​CAC​AAG​TCG​GA3′	5′GAA​TTG​CCA​TTG​CAC​AAC​TCT​TT3′
JAK2	5′GGA​ATG​GCC​TGC​CTT​ACA​ATG3′	5′TGG​CTC​TAT​CTG​CTT​CAC​AGA​AT3′
STAT1	5′GCT​GCC​TAT​GAT​GTC​TCG​TTT3′	5′TGC​TTT​TCC​GTA​TGT​TGT​GCT3′
STAT3	5′AGA​ACC​TCC​AGG​ACG​ACT​TTG3′	5′TCA​CAA​TGC​TTC​TCC​GCA​TCT3′

### Total RNA extraction and quantitative reverse transcription PCR (qRT-PCR)

Total RNA was extracted from PBMCs, THP-1 cells, and mouse synovial tissues using the Trizol method. The RNA concentration was determined by UV spectrophotometry, with optimal absorbance values ranging between 1.8 and 2.0. Subsequently, cDNA synthesis was performed through reverse transcription. RT-qPCR was conducted using the SYBR Green PCR Mix kit (Takara, Japan) and the StepOnePlus Real-Time PCR System (CFXconnect, BIO-RAD, USA). The reaction volume for RT-qPCR was set at 10 μL, comprising 5 µL of Power SYBR Green PCR Mix, 3.4 µL of deionized water, 0.3 µL of each primer (forward and reverse), and 1 µL of cDNA. Reaction conditions: first step: 95°C 30s one cycle→95°C 5s→60°C 34s 40 cycles. Step 2: 95°C 5s→60°C 60s→95°C 15s one cycle. Specimens were arranged in duplicate wells, and lysis curves were analyzed upon reaction completion. The ∆Ct value, calculated as the difference between the Ct value of the target gene and the Ct value of the internal reference, was used to represent the mRNA expression level of the target gene through the 2^-∆Ct^ method.

### Western blotting (WB) and protein blot analysis

Cells were lysed using the RIPA method, and protein concentrations were determined using the BCA assay. Samples were separated by 8%–10% SDS-PAGE and transferred onto polyvinylidene difluoride (PVDF) membranes (Sigma-Aldrich, USA) at 250 V. The membranes were blocked with BSA or IBlockTM for 30–60 min at room temperature and then incubated overnight at 4°C with primary antibodies. After extensive washing with TBST, the membranes were incubated with secondary antibodies at room temperature for 1 h. Protein signals were detected using an ultra-sensitive chemiluminescence method (Affinity ECL Reagent: FG-level) and captured with a Tanon-5200 chemiluminescence image analysis system. Primary antibodies used included rabbit antibodies against JAK2, p-JAK2, STAT1/3, p-STAT1/3 (Abcam, UK), rabbit antibodies against murine IL-1β, GAPDH, and rabbit antibodies against IL-6 (Affinity Biosciences, USA). Secondary antibodies used were goat anti-rabbit or anti-mouse antibodies (CST, USA). Grey values were quantified using ImageJ software, and the ratio to GAPDH was used for semi-quantitative analysis.

### Enzyme-linked immunosorbent assay (ELISA)

Cytokine levels in serum, cell culture supernatant, and mouse tissue supernatant were measured using ELISA kits from Xinbosheng Reagent Kit (Beijing, China), Signalway Antibody (SAB, USA), and R&D Systems (USA), following the manufacturer’s protocols.

### Statistical analysis

SPSS 26.0 and GraphPad Prism 8 software were utilized for statistical analyses. For normally distributed data, t-tests or one-way ANOVA followed by LSD *post hoc* tests were employed. Non-normally distributed data were analyzed using Kruskal–Wallis H tests and Mann-Whitney tests. Spearman correlation analysis was used to assess relationships between variables. Receiver Operating Characteristic (ROC) curves were constructed to evaluate diagnostic accuracy, and logistic regression was employed to assess risk factors. Statistical significance was set at *P* < 0.05.

## Results

### Clinical data and laboratory test indices

Table 3 presents the general data and clinical characteristics of the subjects. Age, gender, LY, HDL, and LDLC did not differ significantly among the three groups (*P* > 0.05). Compared to the HC group, the AG group showed significantly higher levels of IL-6, sUA, Crea, eGFR, Cysc, GLU, Globulin, WBC, GR, MO, TG, TC, VLDL, apoA1, and apoB100. Similarly, the IG group exhibited higher levels of IL-6, sUA, Crea, eGFR, Cysc, GLU, Globulin, WBC, GR, LY, MO, TG, TC, VLDL, apoA1, and apoB100 compared to the HC group (all *P* < 0.05). Within the AG group, IL-6, sUA, Cysc, Globulin, ESR, hsCRP, WBC, GR, MO, and apoA1 levels were significantly higher than those in the IG group (all *P* < 0.05).

**TABLE 3:** Comparison of clinical data and laboratory indicators between groups.

Items	Gout group(n = 111)	AG group(n = 55)	IG group(n = 56)	HC group(n = 57)	*F/H* value	*P* Value
Age(years) (‾x±SD)	40.09 ± 10.02	39.73 ± 10.34	40.45 ± 9.78	39.11 ± 11.86	0.22	0.801
Gender F/M	0/111	0/55	0/56	0/57	—	—
sUA(umol/L) (‾x±SD)	502.0 ± 125.0^a^	529.3 ± 138.9^a^ ^,^ ^b^	475.2 ± 103.9^a^	347.8 ± 42.4	46.36	<0.001
Crea(mmol/L) (‾x±SD)	86.05 ± 14.15^a^	84.99 ± 14.54^a^	87.09 ± 13.82^a^	70.99 ± 10.49	7.55	<0.001
eGFR(ml·min^-1^·1.73min^-2^) (‾x±SD)	89.20 ± 13.83^a^	90.51 ± 14.95^a^	87.91 ± 12.64^a^	100.10 ± 15.81	11.02	<0.001
Cysc(mg/L) (‾x±SD)	1.11 ± 0.31^a^	1.04 ± 0.22^a^ ^,^ ^b^	1.18 ± 0.36^a^	0.82 ± 0.13	28.56	<0.001
GLU(mmol/L) (‾x±SD)	5.62 ± 0.62^a^	5.57 ± 0.67^a^	5.68 ± 0.57^a^	4.75 ± 0.70	34.35	<0.001
Globulin(g/L) (‾x±SD)	32.76 ± 3.48^a^	33.53 ± 2.54^a^ ^,^ ^b^	32.00 ± 4.09^a^	29.14 ± 2.93	26.22	<0.001
ESR(mm/1 h)[M(Q_1_,Q3)]	14.00(8.00.17.00)	17.00(14.00.22.00)^b^	10.00(6.00.13.00)	—	6.39	<0.001
hsCRP(mg/L)[M(Q_1_,Q3)]	8.39(2.29.28.39)	28.39(16.64.41.54)^b^	2.33(1.15.4.21)	—	9.05	<0.001
WBC(×10^9^/L) (‾x±SD)	7.62 ± 1.98^a^	8.76 ± 1.87^a^ ^,^ ^b^	6.51 ± 1.36^a^	6.02 ± 1.10	54.76	<0.001
GR(×10^9^/L) (‾x±SD)	4.66 ± 1.14^a^	5.26 ± 0.80^a^ ^,^ ^b^	4.07 ± 1.12^a^	3.52 ± 0.82	51.70	<0.001
LY(×10^9^/L) (‾x±SD)	2.07 ± 0.57^a^	2.06 ± 0.64	2.07 ± 0.50^a^	1.89 ± 0.57	2.27	0.106
Mo(×10^9^/L) (‾x±SD)	0.48 ± 0.14^a^	0.54 ± 0.13^a^ ^,b^	0.41 ± 0.12^a^	0.33 ± 0.10	47.66	<0.001
TG(mmol/L) (‾x±SD)	2.05 ± 0.81^a^	1.94 ± 0.85^a^	2.15 ± 0.77^a^	1.15 ± 0.44	31.41	<0.001
TC(mmol/L) (‾x±SD)	4.77 ± 1.05^a^	4.61 ± 0.94^a^	4.92 ± 1.13^a^	4.32 ± 0.54	6.17	0.003
HDL(mmol/L) (‾x±SD)	1.16 ± 0.28	1.14 ± 0.26	1.18 ± 0.29	1.23 ± 0.30	1.37	0.257
LDLC(mmol/L) (‾x±SD)	2.59 ± 0.63	2.50 ± 0.66	2.68 ± 0.60	2.52 ± 0.46	1.72	0.182
VLDL(mmol/L) (‾x±SD)	0.88 ± 0.28^a^	0.88 ± 0.28^a^	0.97 ± 0.42^a^	0.63 ± 0.21	18.06	<0.001
apoA1(mmol/L) (‾x±SD)	1.14 ± 0.22^a^	1.10 ± 0.22^a^ ^,^ ^b^	1.18 ± 0.20^a^	1.34 ± 0.20	20.05	<0.001
apoB100(mmol/L) (‾x±SD)	0.89 ± 0.20^a^	0.86 ± 0.19^a^	0.91 ± 0.23^a^	0.75 ± 0.12	12.62	<0.001
IL-6(pg/mL)	16.61(7.57.40.98)^a^	40.98(26.71.61.68)^a^ ^,^ ^b^	7.67(6.04.9.55)^a^	4.02(2.12.5.63)	199.90	<0.001

^a^ *P* < 0.05 vs. HC group.

^b^ *P* < 0.05 vs. IG group.

### Multifactorial logistic regression of acute gouty attacks

To enhance model stability, the IG and HC groups were consolidated. Subsequently, all clinical data and serum IL-6 concentrations from the AG and combined groups were subjected to univariate regression analysis. Variables with a significance level of *P* < 0.05 and a Variance Inflation Factor (VIF) less than 10 were selected for inclusion in logistic regression, as detailed in Table 4. Using the forward stepwise regression method with an entry SLE of 0.05 and stay of 0.1, the final logistic regression results identified IL-6 as a significant risk factor for acute gouty attacks.

**TABLE 4:** Univariate and multivariate logistic regression analysis of acute gouty attacks.

Variables	AG group (n = 55)	Combined group (n = 113)	Univariate analysis	Multivariate analysis	
			*P*_*1*_-value	*P*_*2*_-value	OR(95%CI)
IL-6(pg/mL)	40.98(26.71.61.68)	5.72(4.00.7.86)	0.009	0.009	2.175(1.219.3.881)
sUA(umol/L)	529.3 ± 138.9	410.9 ± 101.5	<0.001	0.179	
Globulin(g/L)	33.53 ± 2.54	30.56 ± 3.82	<0.001	0.171	
ESR(mm/1 h)	17.00(14.00.22.00)	10.00(6.00.13.00)	<0.001	0.765	
WBC(×10^9^/L)	8.76 ± 1.87	6.26 ± 1.25	<0.001	0.492	
GR(×10^9^/L)	5.26 ± 0.80	3.79 ± 1.02	<0.001	0.564	
Mo(×10^9^/L)	0.54 ± 0.13	0.37 ± 0.11	<0.001	0.088	
TG(mmol/L)	1.94 ± 0.85	1.65 ± 0.80	0.035	0.134	
apoA1(mmol/L)	1.10 ± 0.22	1.26 ± 0.21	<0.001	0.165	

Note: Multifactorial Logistic Regression for Acute Gouty Attacks. The combined group consists of the IG, and HC, groups. *P*1 values were obtained through unifactorial logistic regression, while *P*2 and OR, values were derived from multifactorial logistic regression.

### Comparative analysis of transcriptional and translational expression of IL-1β, IL-6, JAK2, and STAT1/3 in PBMCs from gout patients and healthy controls

**FIGURE 1:**
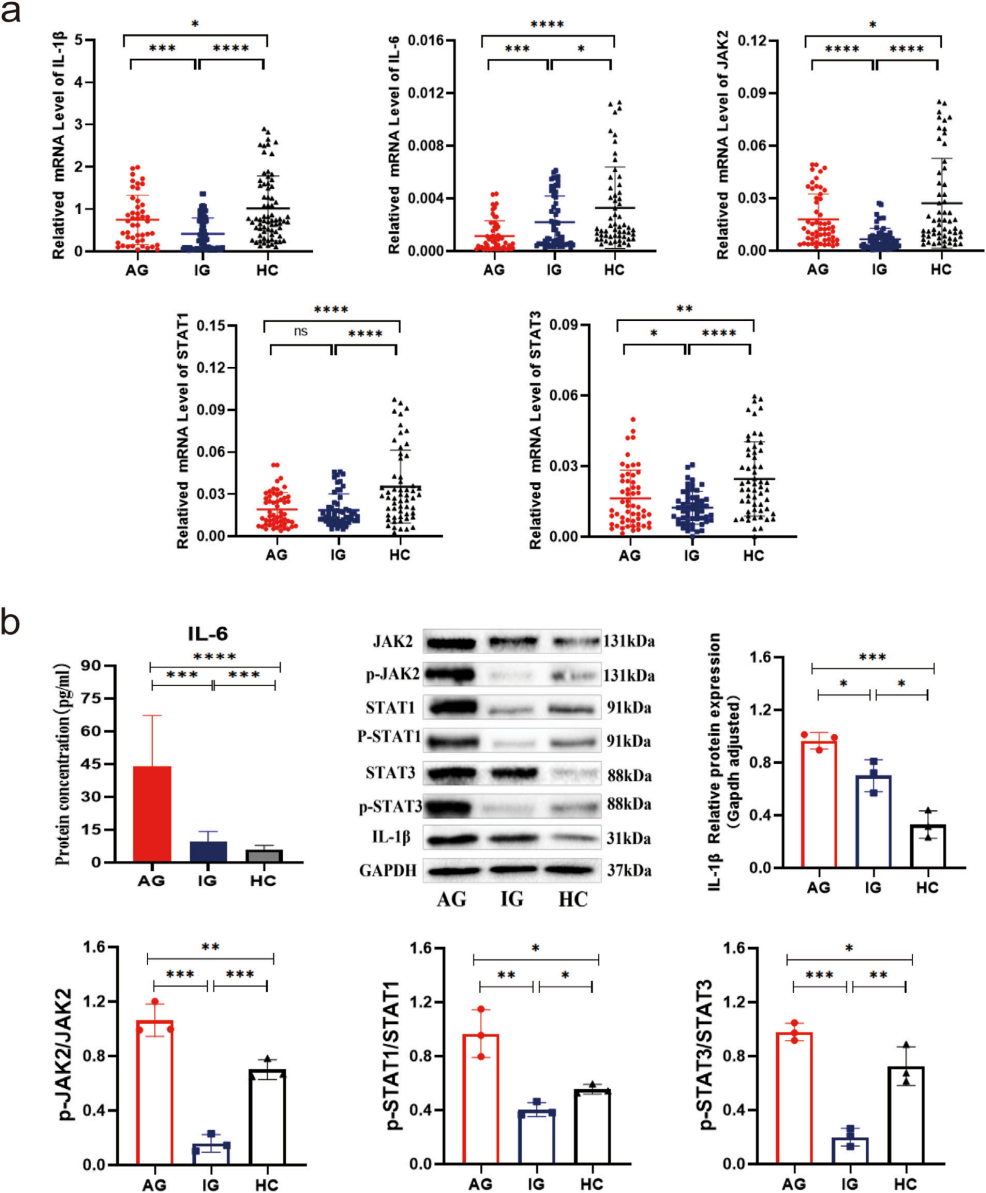
Transcriptional and translational expression of IL-1β, IL-6, JAK2, and STAT1/3 in PBMCs of gout patients and healthy controls. **(a)** Scatter plot showing mRNA expression levels of IL-1β, IL-6, JAK2, and STAT1/3. **(b)** IL-6 serum ELISA results, as well as protein bands and expression histograms for JAK2, STAT1/3, p-JAK2, p-STAT1/3, and IL-1β. Data are expressed as mean ± SD from three independent experiments. *p < 0.05, **p < 0.01, ***p < 0.001, ****p < 0.0001, ns p > 0.05.

The expression levels of IL-1β, IL-6, JAK2, and STAT1/3 mRNA were significantly lower in the gout group compared to the HC group (all *P* < 0.001). Subgroup analysis further revealed statistically significant differences in expression among all three groups (all *P* < 0.001). Specifically, IL-6 mRNA expression was significantly lower in both the AG and IG groups compared to the HC group, with lower levels observed in the AG group compared to the IG group (all *P* < 0.05). The expression of JAK2, STAT3, and IL-1β mRNA was significantly lower in both the AG and IG groups compared to the HC group, and levels were higher in the AG group than the IG group (*P* < 0.05). STAT1 mRNA expression was significantly lower in both the AG and IG groups compared to the HC group (*P* < 0.001), with no statistically significant difference between the AG and IG groups (*P* > 0.05) (Figure 1a). Protein levels of IL-6, JAK2, STAT1/3, p-JAK2, p-STAT1/3, and IL-1β among the three groups showed statistically significant differences (*P* < 0.001). Specifically, the AG group exhibited significantly higher levels compared to the IG and HC groups. Compared to the HC group, the IG group showed significant increases in IL-6, JAK2, STAT3, and IL-1β protein levels, while STAT1, p-JAK2, and p-STAT1/3 protein levels were significantly decreased (*P* < 0.05) (Figure 1b). These findings underscore elevated serum IL-6 levels and dysregulated expression of IL-6/JAK2/STAT1/3 signaling pathway-related genes in gout patients.

### Correlation analysis and ROC curves of IL-6 and JAK2 mRNA or protein expression with inflammatory markers in gout patients

**FIGURE 2:**
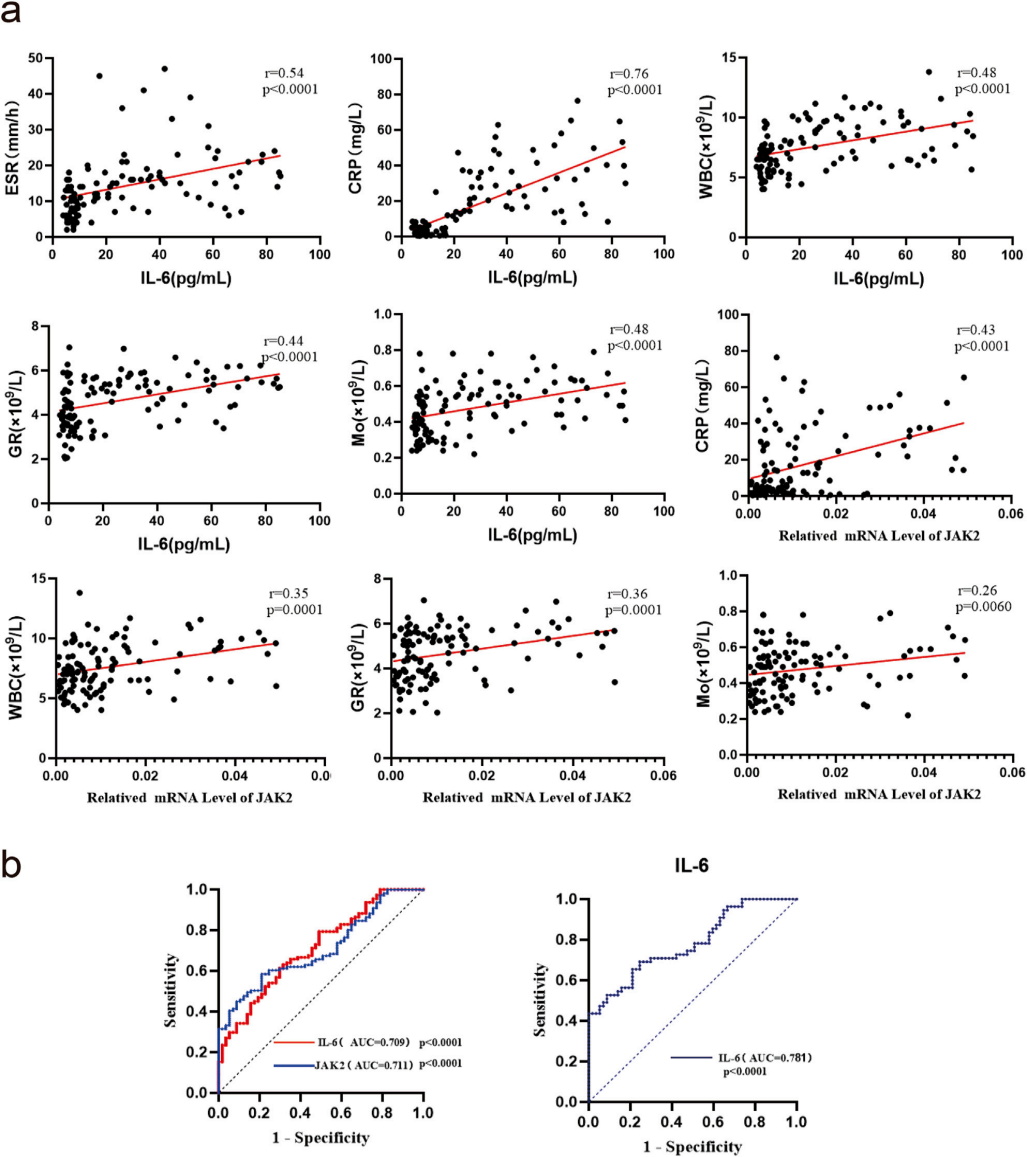
Correlation analysis and ROC curves of IL-6 and JAK2 mRNA or protein expression with inflammatory markers in gout patients. **(a)** Correlation analysis(n = 111). **(b)** ROC curve analysis:the AUC analysis for IL-6 and JAK2 is based on mRNA expression data.

ESR, CRP, WBC, GR, Mo, and LY serve as inflammation-related indicators crucial for assessing disease activity in gouty arthritis. Spearman correlation analysis revealed significant positive associations between ESR, CRP, WBC, GR, Mo, and serum IL-6 protein expression (all *P* < 0.05). Additionally, CRP, WBC, GR, Mo, and JAK2 mRNA expression showed significant positive correlations (*P* < 0.05) (Figure 2a). The Area Under the Curve (AUC) values (95% CI) for IL-6 and JAK2 mRNA expression in GA were 0.709 (0.632, 0.786) and 0.711 (0.631, 0.791), respectively, while for AG, the AUC (95% CI) of IL-6 mRNA expression was 0.781 (0.697, 0.865) (Figure 2b). These findings suggest that IL-6 and JAK2 are linked to both clinical and laboratory aspects of GA and offer additional diagnostic value in the evaluation of gout.

### Changes in the expression levels of IL-1β, IL-6, JAK2 and STAT1/3 in an *in vitro* gouty inflammation model in human blood

**FIGURE 3:**
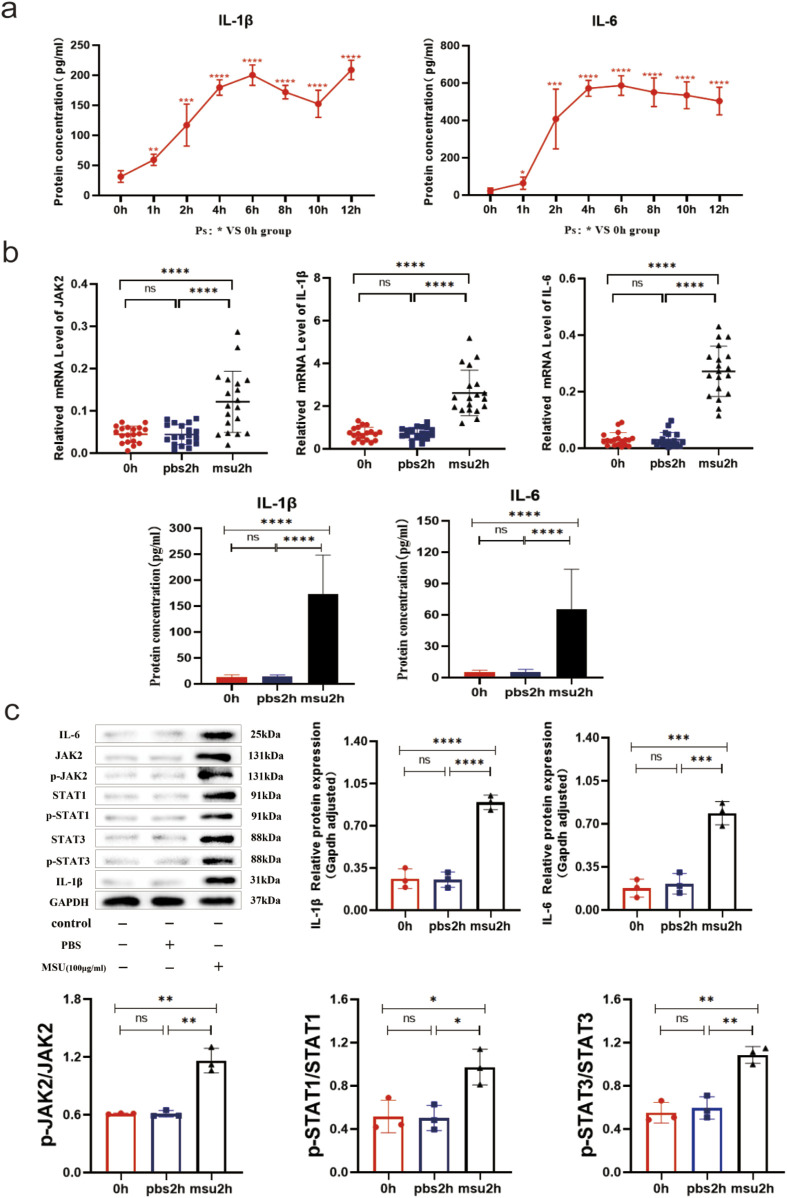
0–12h and 2 h Human Blood *In Vitro* Gout Inflammation Model. **(a)** IL-1β and IL-6 serum ELISA results in the 0–12 h human blood *in vitro* gout inflammation model. **(b)** JAK2 and STAT1/3 mRNA results, along with IL-1β and IL-6 serum ELISA results in the 2 h human blood *in vitro* gout inflammation model. **(c)** Protein bands and expression histograms for IL-6, JAK2, STAT1/3, p-JAK2, p-STAT1/3, and IL-1β in the 2 h human blood *in vitro* gout inflammation model. Data are expressed as mean ± SD from three independent experiments. *p < 0.05, **p < 0.01, ***p < 0.001, ****p < 0.0001, ns p > 0.05.

PBMCs from healthy individuals were stimulated with MSU to establish an *in vitro* model of gout using human blood. The expression of relevant genes was monitored at various time points. Compared to baseline (0 h), both IL-1β and IL-6 protein levels were significantly elevated after 1 h (both *P* < 0.05), with peak inflammation observed at 4–6 h, indicating successful establishment of the acute gout inflammation model (Figure 3a). In the 2-h *in vitro* gout inflammation model using human blood, mRNA expression of IL-1β, IL-6, and JAK2, as well as protein expression of IL-1β, IL-6, JAK2, STAT1/3, p-JAK2, and p-STAT1/3, were significantly higher in the model group compared to both the blank control group and the PBS-negative control group (all *P* < 0.05). No statistically significant differences were observed between the blank control group and the PBS-negative control group (both *P* > 0.05) (Figures 3b, c). These findings suggest that the IL-6-JAK2-STAT1/3 signaling pathway may be involved in the activation of acute gout inflammation or its pathogenesis process.

### In the THP-1 gout inflammation model lasting 6 hours, the addition of an IL-6 agonist enhances the inflammatory response via the JAK2-STAT1/3 signaling pathway

**FIGURE 4:**
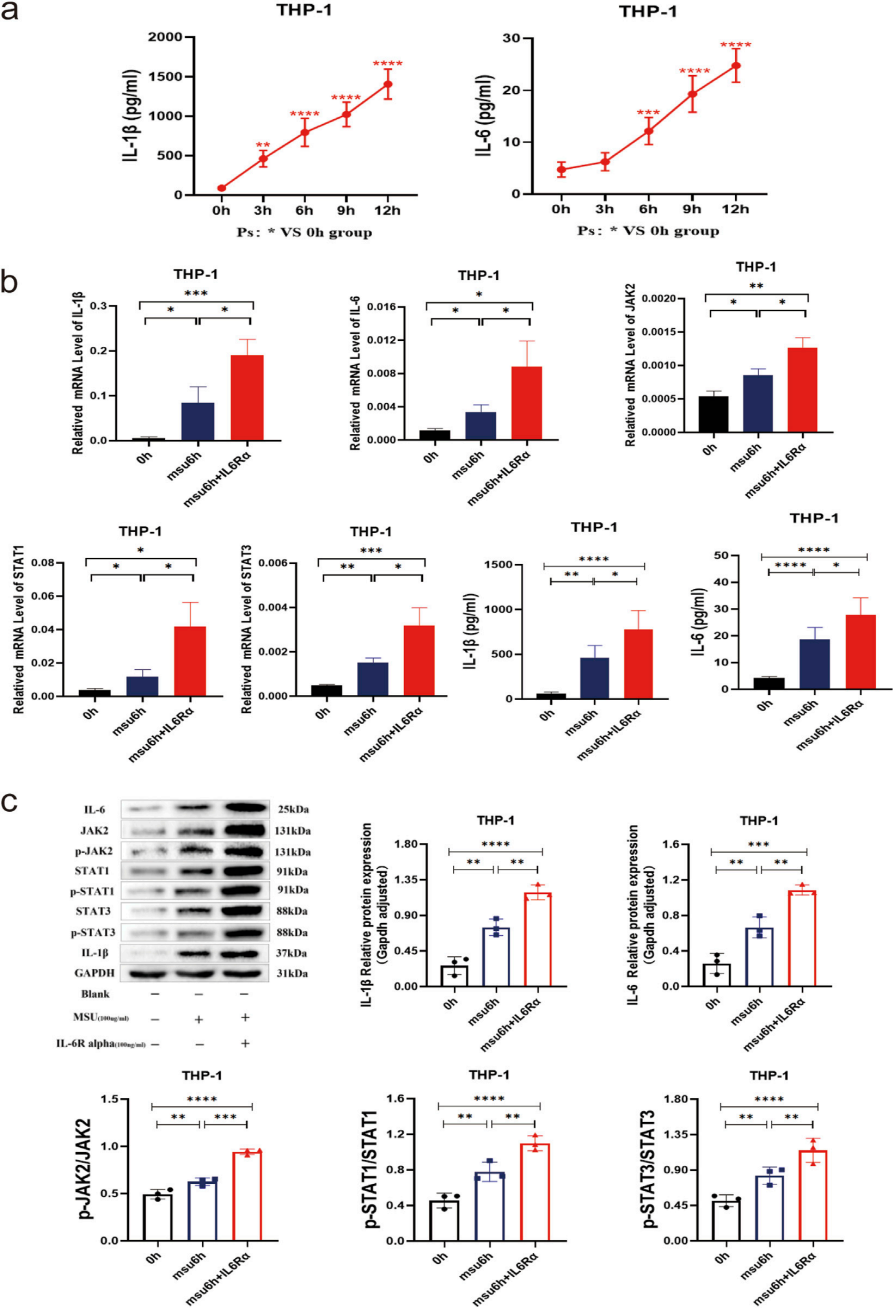
THP-1 Gout 6 h Inflammation Model with Combined IL-6 Agonist (IL-6Rα) to Observe the Potential Role and Effect on the JAK2-STAT1/3 Signaling Pathway. **(a)** ELISA results of IL-1β and IL-6 in supernatants from the 0–12 h THP-1 gout inflammation model. **(b)** IL-1β, IL-6, JAK2, and STAT1/3 mRNA results, along with IL-1β and IL-6 supernatant ELISA results in the THP-1 gout 6 h inflammation model with combined IL-6 agonists. **(c)** Protein bands and expression histograms for IL-6, JAK2, STAT1/3, p-JAK2, p-STAT1/3, and IL-1β in the THP-1 gout 6 h inflammation model with combined IL-6 agonists. Data are expressed as mean ± SD from three independent experiments. *p < 0.05, **p < 0.01, ***p < 0.001, ****p < 0.0001, ns p > 0.05.

THP-1 macrophages were stimulated with MSU to establish an acute gout cell model, and gene expression was monitored at different time points. Compared to baseline (0 h), the expression of IL-1β and IL-6 proteins gradually increased, becoming statistically significant after 3 h (both *P* < 0.05). Analysis from the data suggests that inflammation peaks after 12 h (Figure 4a). In the 6-h cellular model of acute gout, the expression levels of IL-1β, IL-6, JAK2, STAT1/3 mRNA, and their respective proteins, including phosphorylated forms, were significantly higher in the model group compared to the blank control group (all *P* < 0.05) (Figure 4b). Additionally, when an IL-6 agonist was introduced to the model group, the expression of IL-1β, IL-6, JAK2, STAT1/3 mRNA, as well as IL-1β, IL-6, JAK2, STAT1/3, p-JAK2, and p-STAT1/3 proteins, showed significant elevation compared to the model group without agonist addition (all *P* < 0.05) (Figure 4c). These findings indicate that IL-6 agonists intensify the inflammatory response and amplify inflammation through the JAK2-STAT1/3 signaling pathway.

### IL-6 knockout mice (IL-6 KO) exhibit milder arthritis compared to wild-type B6 mice (WT)

**FIGURE 5:**
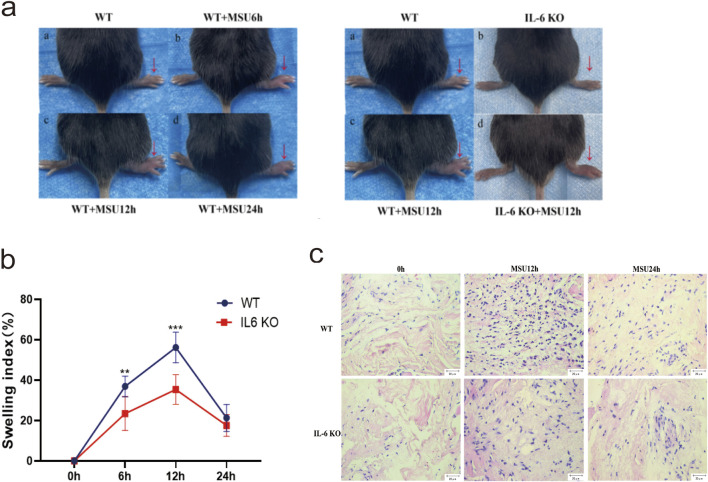
IL-6 KO Mouse and WT Mouse Gout Models. **(a)** 150 µL of MSU (80 mg/mL) was injected into the right foot pads of mice, and the thickness of the foot pads was measured at 0, 6, 12, and 24 h, with photographs taken at each time point. The WT and WT + MSU12 h panels are re-used for illustrative purposes. **(b)** Swelling index plots of the foot pads at 0, 6, 12, and 24 h. Data are expressed as mean ± SD from three independent experiments(n = 8). *p < 0.05, **p < 0.01, ***p < 0.001. **(c)** HE staining of synovial tissue in the foot pads at 0, 12, and 24 h (scale = 20 μm, magnification ×400). Blue staining indicates inflammatory cell infiltration.

MSU crystals were injected into the footpads of IL-6 KO and WT mice to establish an acute gouty arthritis model. The left panel of Figure 5a shows that the swelling index of footpads in WT mice significantly differed from the baseline (0 h) at 6, 12, and 24 h (*P* < 0.05), confirming the successful establishment of the gout model. The right panel of Figure 5a illustrates that, at 12 h, the footpads of WT mice were more visibly swollen and exhibited a higher swelling index than those of IL-6 KO mice (Figure 5a). Specifically, at 6 and 12 h post-injection, the swelling index of footpads in IL-6 KO mice was significantly lower than that in the WT control group(*P* < 0.05). The most substantial difference was observed at 12 h, prompting further experimental focus on this time point (Figure 5b). HE staining revealed more pronounced inflammatory cell infiltration in WT mice at 12 h and 24 h compared to IL-6 KO mice, with no significant difference observed at 0 h (Figure 5c). These results underscore that IL-6 knockout mitigates MSU-induced inflammation and arthritis in the experimental model.

### IL-6 KO mice avoid developing more severe gouty arthritis by impairing the JAK2-STAT1/3 signalling pathway

**FIGURE 6:**
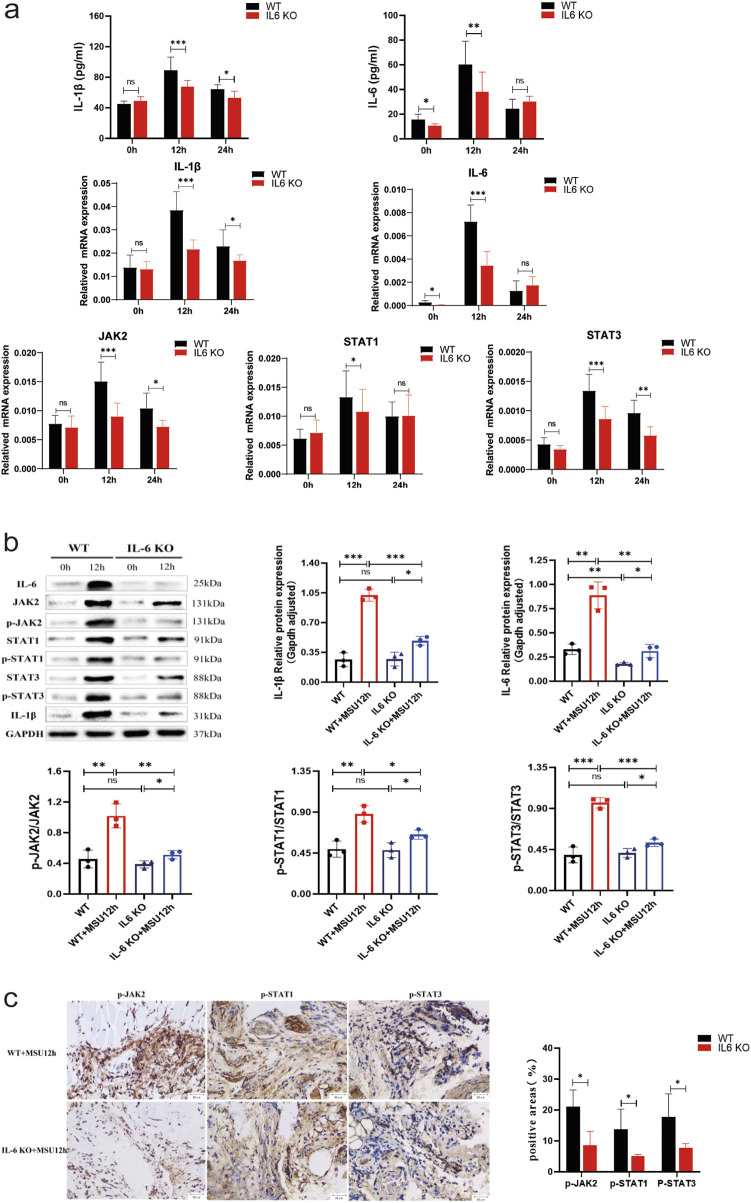
Observation of Potential Roles and Effects of IL-6 Knockout on the JAK2-STAT1/3 Signaling Pathway in a Mouse Model of Acute Gouty Arthritis. **(A)** ELISA results of IL-1β and IL-6 in tissue supernatants, and mRNA results of IL-1β, IL-6, JAK2, and STAT1/3. **(B)** Protein bands and expression histograms for IL-6, JAK2, STAT1/3, p-JAK2, p-STAT1/3, and IL-1β. **(C)** Images of IHC staining for p-JAK2 and p-STAT1/3 in the footpad synovium after 12 h of MSU stimulation, along with results of positive areas (scale = 20 μm, magnification ×400). Tan coloration indicates positive expression. Data are expressed as mean ± SD from three independent experiments. *p < 0.05, **p < 0.01, ***p < 0.001, ****p < 0.0001, ns p > 0.05.

In IL-6 KO mice, both mRNA and protein levels of IL-6 were significantly reduced compared to untreated WT mice (*P* < 0.05), while the transcription and translation of other genes remained comparable. This suggests that heterozygous IL-6 KO mice may have been utilized to generate the gout model (Figures 6a, b). In the acute gout model, the mRNA and corresponding protein(contain their phosphorylated proteins) levels of IL-1β, IL-6, JAK2, and STAT1/3 were significantly lower in IL-6 KO mice compared to WT mice at 12 h post-injection (*P* < 0.05) (Figures 6a, b); Additionally, IHC staining revealed a decrease in the positive expression of phosphorylated JAK2 and STAT1/3 in IL-6 KO mice (*P* < 0.05) (Figure 6c). At 24 h, while IL-6 mRNA and protein expression levels were similar to WT mice (*P* > 0.05), IL-1β mRNA and protein levels, as well as JAK2 and STAT3 mRNA, were significantly downregulated in IL-6 KO mice (*P* < 0.05). In contrast, STAT1 mRNA expression remained unchanged (*P* > 0.05) (Figures 6a, b). These findings suggest that IL-6 deletion not only reduces inflammation but also mitigates the severity of gouty arthritis by impairing the JAK2-STAT1/3 signaling pathway.

## Discussion

**FIGURE 7:**
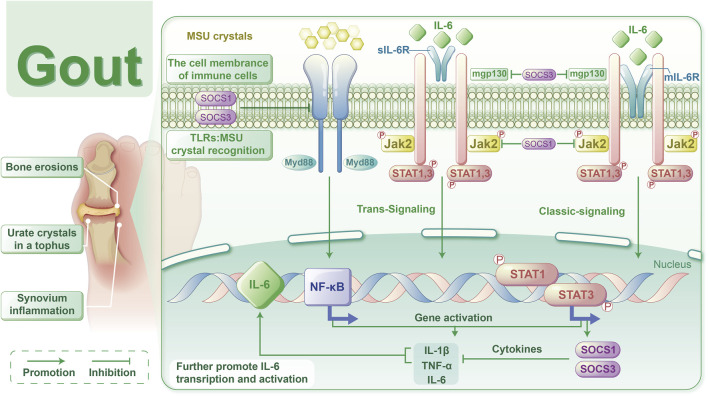
Schematic representation of the mechanism by which the IL-6-mediated JAK2-STAT1/3 signalling pathway affects gout.

GA is a clinical syndrome precipitated by a persistent increase in blood uric acid levels, resulting in the abnormal accumulation of MSU crystals in joints and tissues. This condition manifests as joint swelling, severe pain, and restricted movement, largely due to the release of inflammatory mediators, such as cytokines and chemokines, from cells within the affected joints. Consequently, managing inflammation is vital for preventing GA attacks. In our study, we noted elevated levels of IL-6, ESR, hsCRP,WBC, GR, and Mo in GA patients compared to the HC group. These markers were significantly more elevated in the AG group than in the IG group, indicating a pronounced increase in serum IL-6 and other inflammatory markers in gout patients during acute episodes (Table 3). Moreover, logistic regression analysis identified IL-6 as a significant risk factor for acute gout attacks (Table 4). The development of AGA is strongly associated with the production of IL-6 and IL-1 (Figure 7). Initially, MSU crystals trigger the MYD88-NFκB signaling pathway *via* Toll-like receptors (TLRs) on immune cell membranes, leading to the release of cytokines such as IL-6 and IL-1β. IL-6 interacts with either membrane-bound (mIL-6R) or soluble (sIL-6R) receptors, in conjunction with gp130, to activate the JAK2/STAT signaling pathway. This activation promotes the transcription and expression of downstream genes. Recent studies have shown (Kothari et al., 2021) that prolonged stimulation by IL-1 increases the phosphorylation of STAT proteins (STAT1/3/5) across various immune cells, with IL-1β-induced IL-6 leading to later activation and phosphorylation of STAT1/3. Consequently, we hypothesize that the IL-6-mediated JAK2-STAT1/3 pathway contributes to the progression of gout, possibly enhancing TLRs-mediated mechanisms. Previous research has mainly focused on downstream effects, but our study is the first to clinically validate IL-6 as a risk factor for gout. Moreover, IL-6 acts upstream of JAK2/STAT1/3, initiating an inflammatory cascade that, through positive feedback, produces more IL-6, thus exacerbating inflammation. In animal models, targeting IL-6 disrupted the IL6-JAK2/STAT1/3-IL6 feedback loop, offering a potential therapeutic approach for effectively treating gout. This strategy could lead to novel therapeutic methods or drugs that target the IL-6 signaling pathway in gout management.

To date, the TLR4 receptor is recognized as the primary sensor in gout, capable of recognizing pathogen-associated molecular patterns (PAMPs) and damage-associated molecular patterns (DAMPs) to initiate signaling. Subsequently, MyD88 mediates the translocation of NF-κB to the nucleus, enhancing the transcription of IL-6 and other pro-inflammatory cytokines, such as TNF-α and IL-1β, in monocytes (Silva et al., 2023). Furthermore, IL-6 mRNA transcription can be stimulated by signals from TNF or IL-1. Beyond monocytes and cytokines, stromal cells, certain immune cell subsets, lipid mediators, and adipokines also produce IL-6 in response to cellular stress induced by Toll-like receptor agonists (Millrine et al., 2022). IL-6 serves as a key immunomodulatory cytokine, influencing the pathogenesis of autoimmune diseases, chronic inflammatory conditions, cancers, and other disorders. It induces intracellular signaling through the JAK/STAT, Ras/MAPK, and PI3K pathways. Within the JAK/STAT pathway, dimerization of Gp130 results in the proximity of JAKs, leading to phosphorylation of tyrosine residues on the Gp130 cytoplasmic domain. Molecules containing the Src homology 2 (SH2) structural domain, STAT1/3 and protein tyrosine phosphatase 2 (SHP2) containing the SH2 structural domain are attracted to the tyrosine phosphorylation motif of gp130. This recruitment facilitates the phosphorylation of STAT1/3 by JAKs, which then translocate to the nucleus to activate transcriptional outputs and trigger the mitogen-activated protein kinase pathway *via* SHP2. Concurrently, STAT3 activation induces various IL-6 response genes, including acute phase proteins. STAT3 also induces SOCS1 and SOCS3, which bind to phosphorylated JAK and phosphorylated Gp130, respectively, forming a negative feedback loop to terminate IL-6 signal transduction (Figure 7). Multiple mechanisms regulate IL-6 expression; however, its abnormal expression plays a crucial role in the pathogenesis of various autoimmune and inflammatory diseases (Aliyu et al., 2022). This study revealed decreased mRNA levels of IL6-JAK2-STAT1/3 in the peripheral blood of gout patients compared to the HC group (Figure 1a), suggesting a potential negative feedback mechanism in humans. Additionally, the levels of IL-6 protein and JAK2 mRNA were positively correlated with certain inflammatory markers(Figure 2a), reflecting their association with gout’s clinical and laboratory activities. These findings align with Nara Gualberto Cavalcanti’s research (Cavalcanti et al., 2016), which also associated IL-6 with tophi presence and joint deformities in gout patients. Elevated IL-6 levels in children with hyperuricemia (Di Y et al., 2018) also correlated with disease activity, an interesting parallel. Subgroup analysis showed significant increases in IL-1β and IL6-JAK2-STAT1/3 proteins and their phosphorylated forms in the AGA group, while the IG group exhibited higher levels of IL-1β and total IL6-JAK2-STAT3 proteins, with decreased phosphorylated STAT1 and JAK2-STAT1/3 proteins (Figure 1b). This suggests a crucial role for phosphorylated proteins in gout’s inflammatory response and indicates involvement of the IL-6 and JAK2-STAT1/3 signaling pathways in gout pathogenesis. Further subgroup analysis indicated reduced expression of JAK2-STAT1/3 proteins and their phosphorylated forms in the IG group compared to the AG group (Figure 1b). This suggests a decrease in JAK2-STAT1/3 signaling pathway activation as gout transitions to the intercritical phase. The spontaneous resolution of acute gout attacks may relate to this pathway’s downregulation, paralleling findings by Jumpei Temmoku et al. (Temmoku et al., 2021). Contrarily, IL-6 protein levels in peripheral blood serum were significantly higher in the AG group than in the IG group, indicating rapid increases during acute gout attacks. This rise in IL-6 could potentially trigger JAK2 activation, thereby initiating the JAK2-STAT1/3 signaling pathway and intensifying the inflammatory response. Numerous studies have linked excessive or sustained IL-6 production with various inflammatory diseases (Narazaki and Kishimoto, 2018) supporting the hypothesis that IL-6 dysregulation plays a critical role in gout pathogenesis. Additionally, in the MSU-induced human blood *ex vivo* gout model over 0–12 h (Figure 3a), IL-1β and IL-6 protein expression levels increased at 1 h and peaked between 4 and 6 h compared to baseline. Furthermore, the 2-h human blood *ex vivo* gout inflammation model (Figures 3b, c) showed significantly elevated levels of IL-1β, IL-6, JAK2 mRNA, and their respective proteins, including phosphorylated JAK2 and STAT1/3, compared to both control groups. Collectively, these data indicate that the IL-6/JAK2/STAT1/3 signaling pathway may play a role in the activation of acute gout inflammation and its pathogenesis.

The JAK/STAT pathway is integral to signal transduction driven by extracellular cytokine-activated receptors, playing critical roles in cell proliferation, differentiation, apoptosis, organ development, and immune homeostasis (Xin et al., 2020). Biologic therapies highlight cytokines as key mediators of immune-driven diseases, with JAK inhibitors proving to be safe and effective for treating numerous autoimmune and inflammatory conditions (Schwartz et al., 2017). Research extensively shows that IL-6 regulates nuclear target genes *via* the JAK2-STAT1/3 pathway (Zeng et al., 2023; Deng et al., 2023). To determine whether IL-6 agonists exacerbate gouty arthritis *via* the JAK2-STAT1/3 signaling pathway, we conducted *in vitro* experiments. Therefore, THP-1 cells were treated with IL-6 agonists to establish an acute gouty inflammation model. In a gout model using THP-1 cells treated with MSU at different time points, varying degrees of upregulation in IL-1β and IL-6 proteins were observed over time (Figure 4a). In the 6-h acute gout cell model, expression levels of IL-1β, IL-6, JAK2, STAT1/3 mRNA, and their respective proteins—including phosphorylated JAK2 and STAT1/3—were significantly elevated in the model group compared to the blank control group. Moreover, treatment with an IL-6 agonist further increased these expression levels compared to the untreated model group, demonstrating a notable enhancement in inflammatory signaling (Figures 4b, c). These results suggest that the IL-6 agonist enhances the expression of the JAK2-STAT1/3 pathway, intensifying the inflammatory response and indicating that it amplifies inflammation *via* this signaling route. Supporting evidence indicates that IL-6 activates the JAK2/STAT3/SOCS3 pathway, playing a critical role as an inflammatory cytokine that promotes both pro-inflammatory and anti-inflammatory responses (Wang et al., 2013). Furthermore, activation of the JAK2/STAT3 pathway has been implicated in uric acid-induced kidney damage and the overproduction of inflammatory cytokines (Lin et al., 2021). It has also been reported that purine-induced interferon-γ activates STAT1 and, in synergy with interferon regulatory factor 1, upregulates xanthine oxidoreductase expression, promoting uric acid generation and inducing inflammation (Wang et al., 2022). Moreover, studies involving LPS-induced macrophages and adjuvant-induced arthritis in rats demonstrate that the production of pro-inflammatory cytokines such as IL-6, IL-1β, and TNF-α requires activation through the NF-κB, JAK1-STAT1/3, and MAPK signaling pathways to exert inflammatory effects (Luan et al., 2022). In summary, once inflammatory mechanisms are activated, IL-6 plays a crucial role in acutely amplifying its signaling pathways. IL-6 activation of the JAK2-STAT1/3 signaling pathway stimulates acute-phase protein production and induces leukocytosis, fever, and angiogenesis during the acute phase. In later stages, IL-6 promotes the transition to chronic inflammation by sustaining monocyte chemoattractant protein-1 secretion, vascular proliferation in T cells, and anti-apoptotic functions, facilitating mononuclear cell aggregation at the injury site. Overall, these findings highlight IL-6’s role and impact as a cytokine that promotes autoimmune phenomena and amplifies acute inflammation *via* the JAK2-STAT1/3 signaling pathway.

The JAK/STAT pathway is a principal signaling cascade regulated by cytokines, essential for initiating innate immunity, coordinating adaptive immune responses, and ultimately moderating inflammation. To explore whether IL-6 KO alleviates gouty arthritis through the JAK2-STAT1/3 pathway, and to confirm the role of IL-6 KO in the inflammatory response induced by MSU crystals, we conducted *in vivo* experiments in mice. MSU crystals were injected into the footpads of both WT and IL-6 KO mice to simulate human AGA. In this model, WT mice developed more severe arthritis compared to IL-6 KO mice. In this model, WT mice exhibited more severe arthritis and greater footpad swelling than IL-6 KO mice, as consistently documented (Figures 5a, b). Histological analysis with HE staining showed increased inflammatory cell infiltration in WT mice compared to IL-6 KO mice (Figure 5c). These observations suggest that genetic deletion of IL-6 mitigates MSU-induced inflammation and arthritis, highlighting the potential of targeting IL-6 as a therapeutic approach for managing MSU-induced arthritis. In IL-6 KO mice, basal transcription and translation levels of JAK2-STAT1/3 remained unaffected. However, during MSU-induced arthritis, these levels were significantly reduced (Figures 6a, b), indicating that targeting IL-6 can inhibit the activation and phosphorylation of the JAK2-STAT1/3 pathway, thereby alleviating arthritis inflammation. After establishing the acute gout mouse model with MSU (Figure 6), significant downregulation in the transcription and translation levels of IL-1β and IL-6-mediated JAK2-STAT1/3 signaling was observed in IL-6 KO mice compared to WT mice at 12 h. Moreover, at 24 h, there was a decrease in IL-1β mRNA and protein expression, along with reduced expression of JAK2 and STAT3 mRNA in IL-6 KO mice compared to WT mice. The combined trends of IL-1β and IL-6 suggest that IL-6 gene knockout attenuates the JAK2-STAT1/3 signaling pathway, inhibiting pro-inflammatory cytokine production and alleviating MSU-induced gouty arthritis. These findings corroborate that IL-6 gene knockout can downregulate inflammation through the JAK2-STAT1/3 pathway. Literature reviews, coupled with network pharmacology and bioinformatics predictions, have identified IL-6 and STAT1/3 as critical targets for anti-gout treatment (Yang et al., 2023; Liu et al., 2022). These targets modulate the IL-6/STAT1/STAT3 pathway, which has been shown to significantly prevent and treat gout and arthritis. Similar therapeutic outcomes and mechanisms have been observed with the use of extracts from Ephedra sinica (Han et al., 2016) and Simiao Wan(Shi et al., 2021) in managing gouty arthritis. Evidence suggests (Yen et al., 2015; Jaramillo et al., 2004) that targeting the JAK2 or JAK2/STAT1α pathways induced by MSU crystals in macrophages can release anti-inflammatory mediators, counteracting the formation of pro-inflammatory cytokines. Previous studies have implicated the JAK2/STAT3 signaling pathway and downstream IL-6 in uric acid-induced kidney injury, highlighting potential strategies for preventing and treating hyperuricemia-associated kidney damage. Extracts of Cortex Phellodendri (Pan et al., 2021) and berberine (Lin et al., 2021) reportedly reduce the invasion of inflammatory factors and uric acid accumulation in the kidneys by inhibiting STAT3 expression or activating the JAK2/STAT3 signaling pathway, thereby alleviating hyperuricemic nephropathy progression. Based on the above, inhibiting or reducing IL-6 expression and the IL-6-mediated JAK2-STAT1/3 signaling pathway in AGA can alleviate the severity of MSU crystal-induced arthritis and inflammation. This evidence could serve as a foundation for developing new therapeutic approaches and medications for treating gout.

## Conclusion

Previous studies have largely concentrated on the JAK2/STAT3 or JAK2/STAT1α signaling pathways and their downstream mediator, IL-6. Our study enriches this field by demonstrating that IL-6 acts as an upstream regulator of the JAK2-STAT1/3 signaling pathway. For the first time, our research identifies IL-6 as a risk factor for acute gout attacks, elucidating that the IL-6-mediated JAK2-STAT1/3 signaling pathway participates in the inflammation and pathogenesis of acute gout through positive feedback mechanisms. Overall, targeting IL-6 signaling could be an effective therapeutic strategy for treating gout or managing gout attacks.

## Data Availability

The original contributions presented in the study are included in the article/Supplementary Material, further inquiries can be directed to the corresponding authors.
