# Supplementary figures and images for: The role and impact of the IL-6 mediated JAK2-STAT1/3 signaling pathway in the pathogenesis of gout (part 1 of 2)
Source: Front Pharmacol. 2025 Mar 18;16:1480844. doi: 10.3389/fphar.2025.1480844 (PMC11959054; doi:10.3389/fphar.2025.1480844)

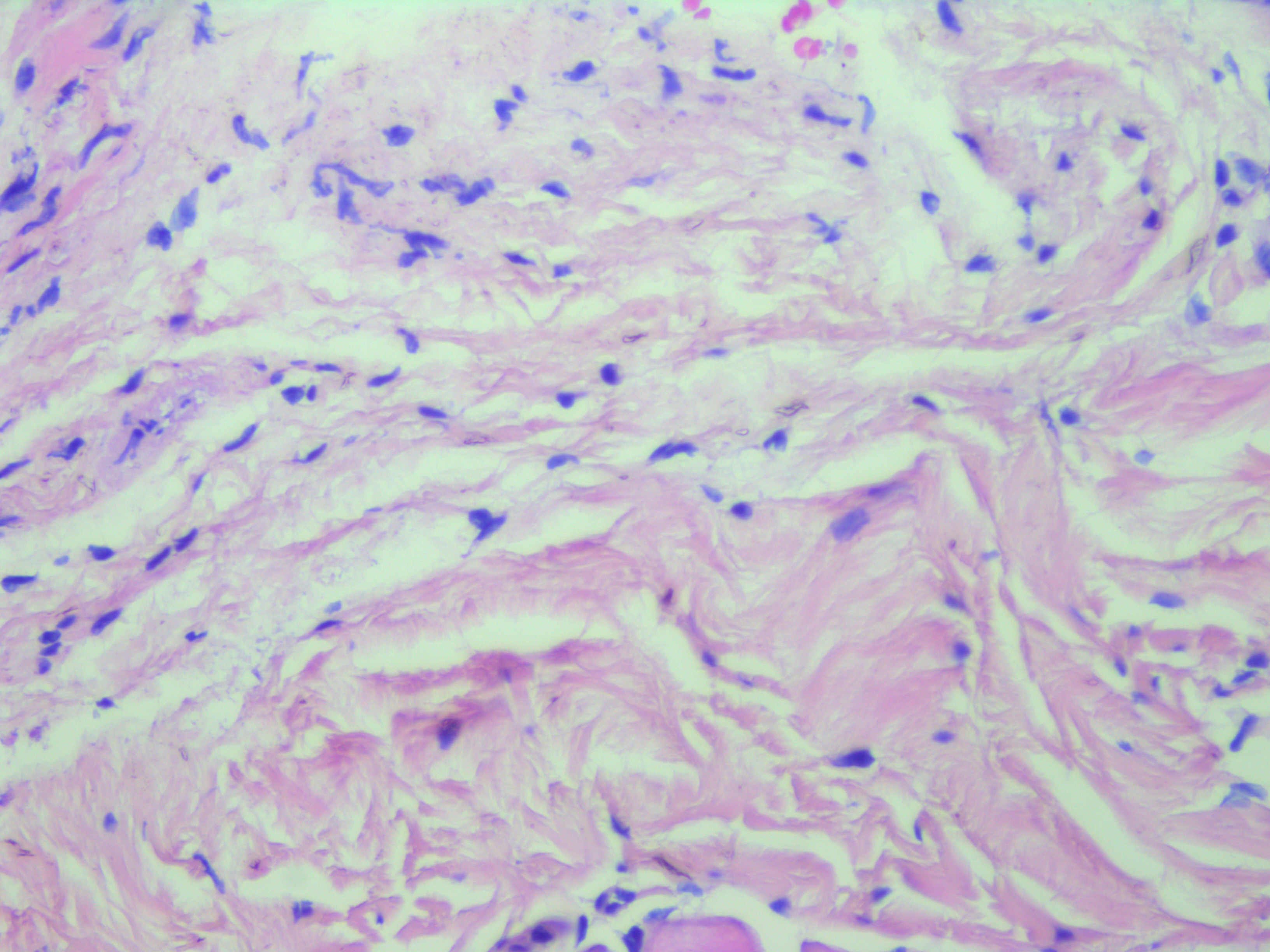

Supplement: Supplementary file 2 [file DataSheet3.zip › WT House HE staining/WT 0h.jpg]

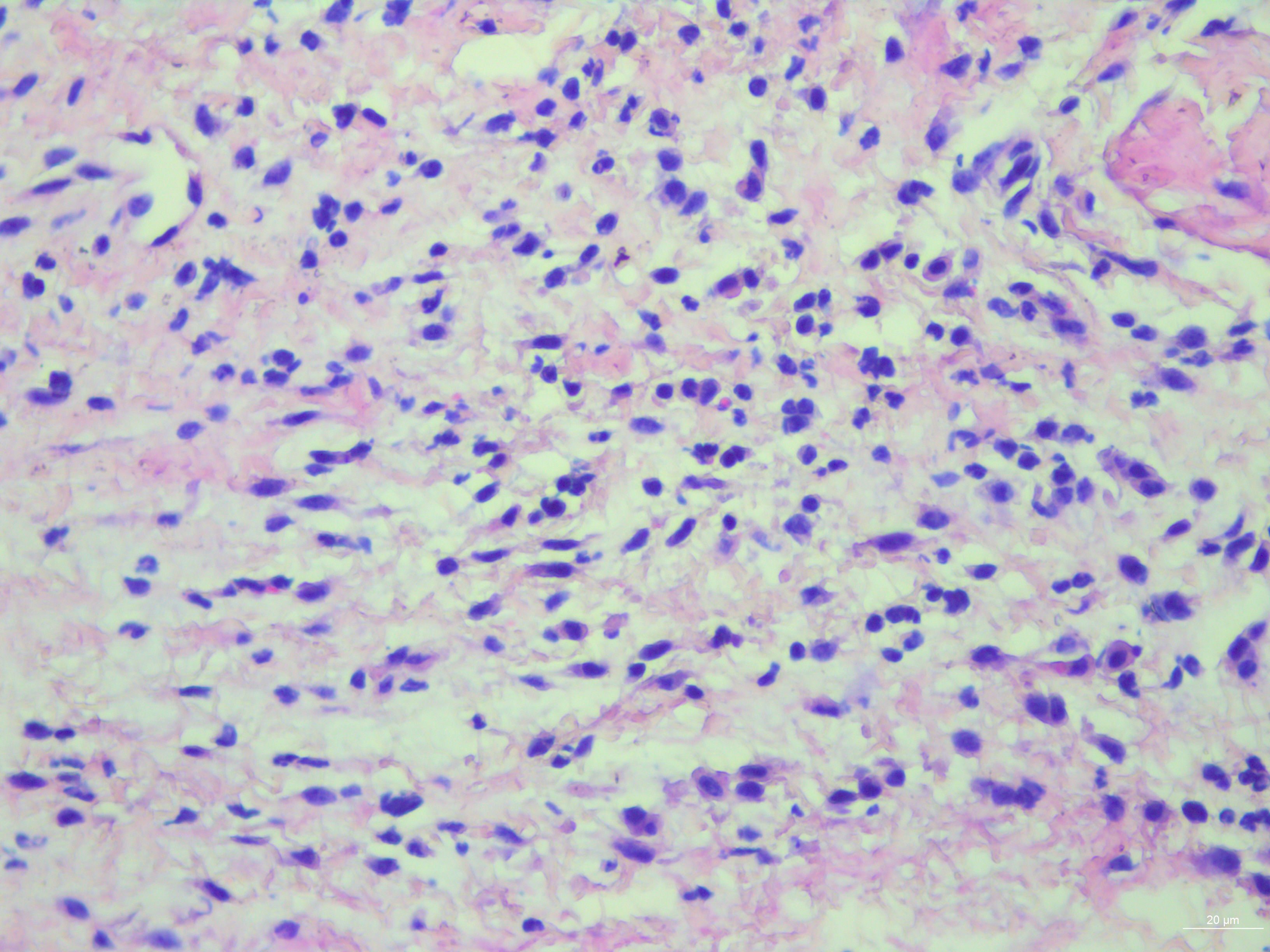

Supplement: Supplementary file 2 [file DataSheet3.zip › WT House HE staining/WT MSU12h.jpg]

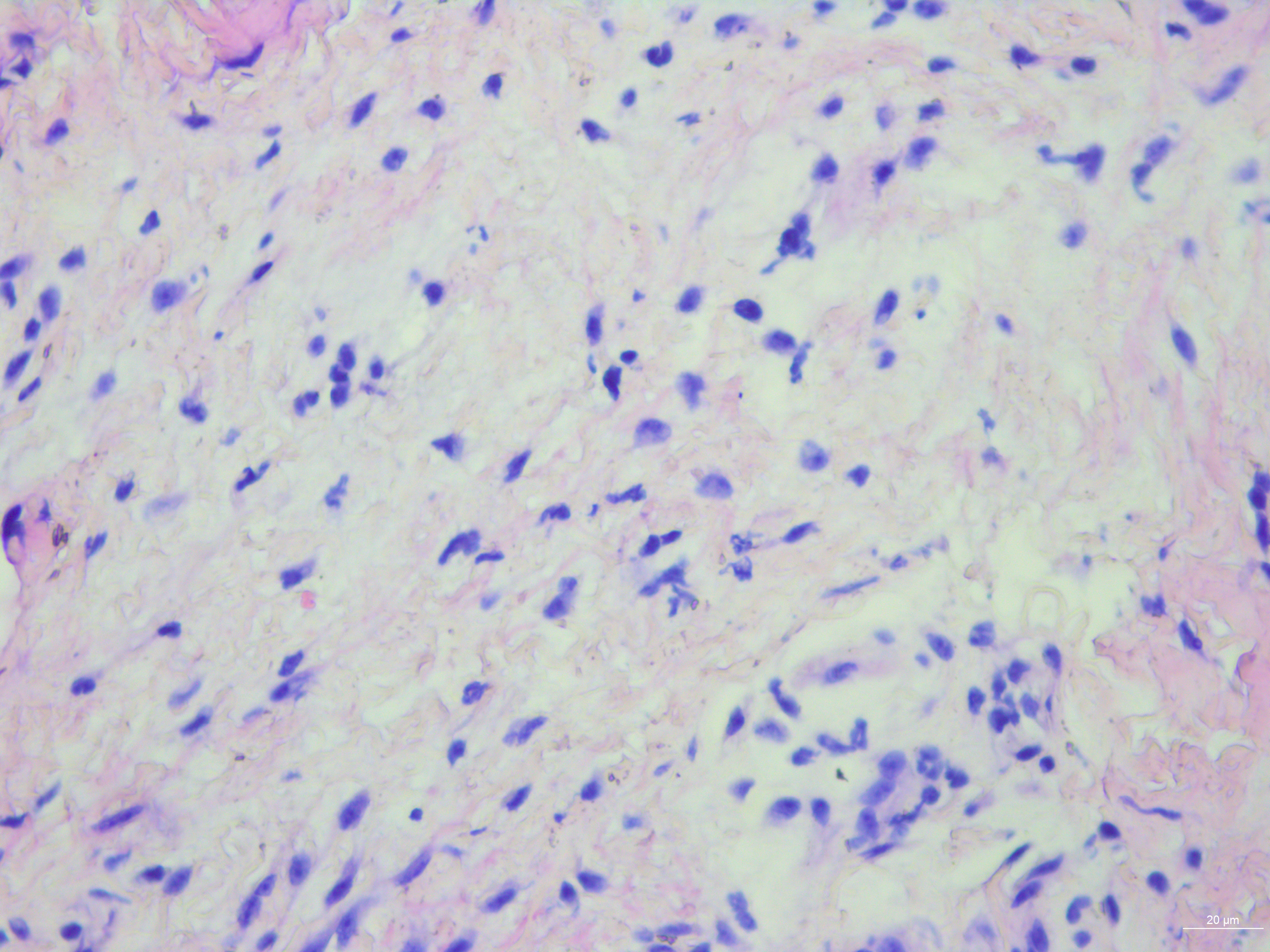

Supplement: Supplementary file 2 [file DataSheet3.zip › WT House HE staining/WT MSU24h.jpg]

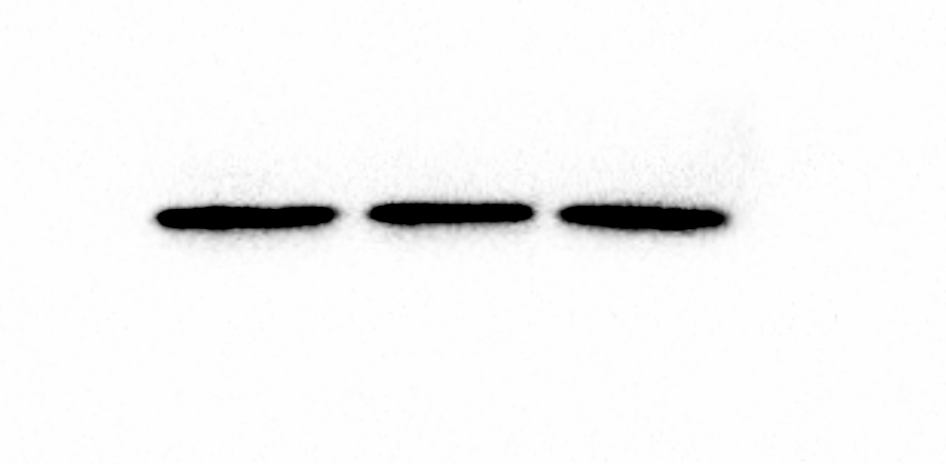

Supplement: Supplementary file 3 [file DataSheet8.zip › WB bands for each gene listed in the article/1、AGIGHC/GAPDH-1.tif]

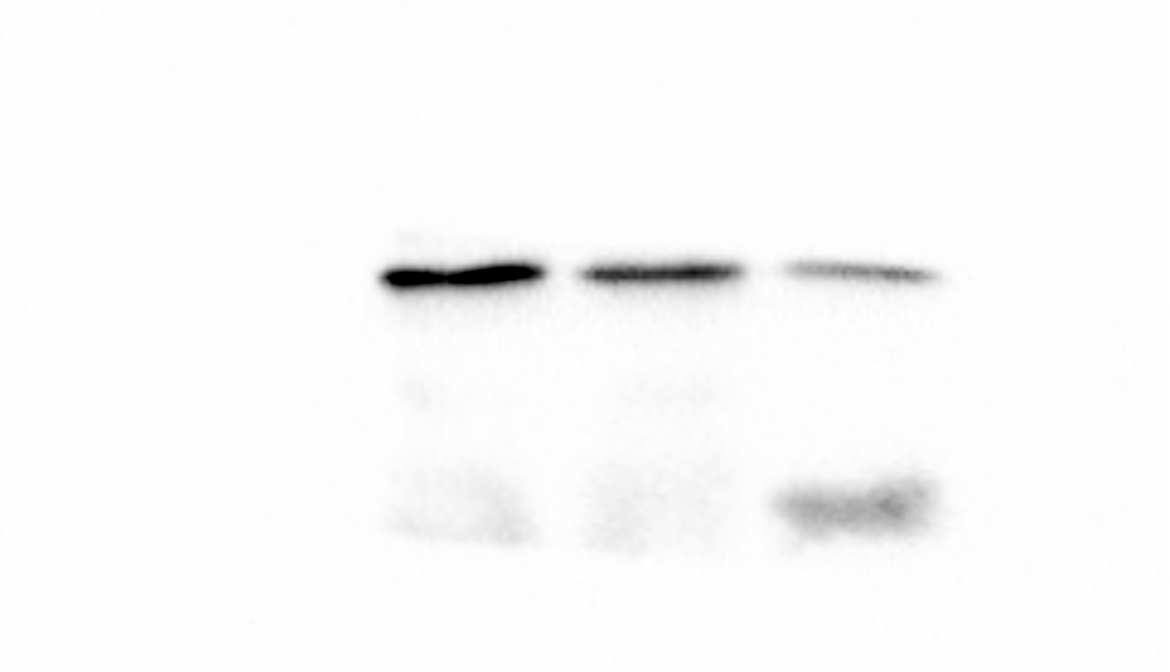

Supplement: Supplementary file 3 [file DataSheet8.zip › WB bands for each gene listed in the article/1、AGIGHC/IL-1β-1.tif]

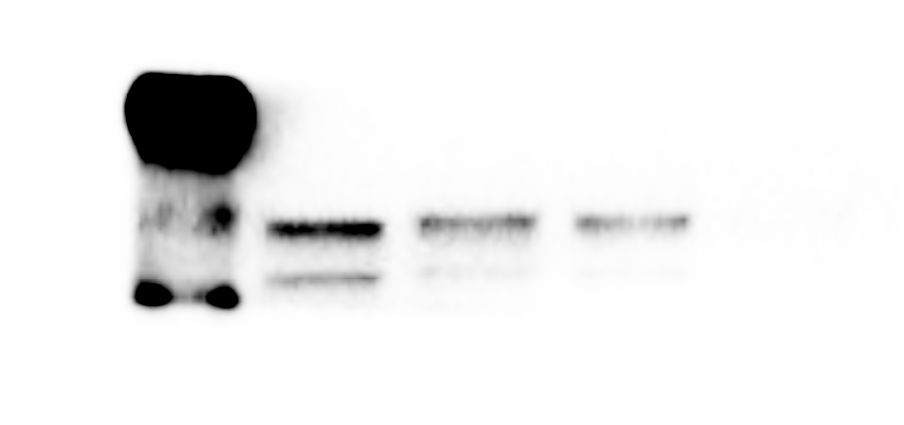

Supplement: Supplementary file 3 [file DataSheet8.zip › WB bands for each gene listed in the article/1、AGIGHC/JAK2-1.tif]

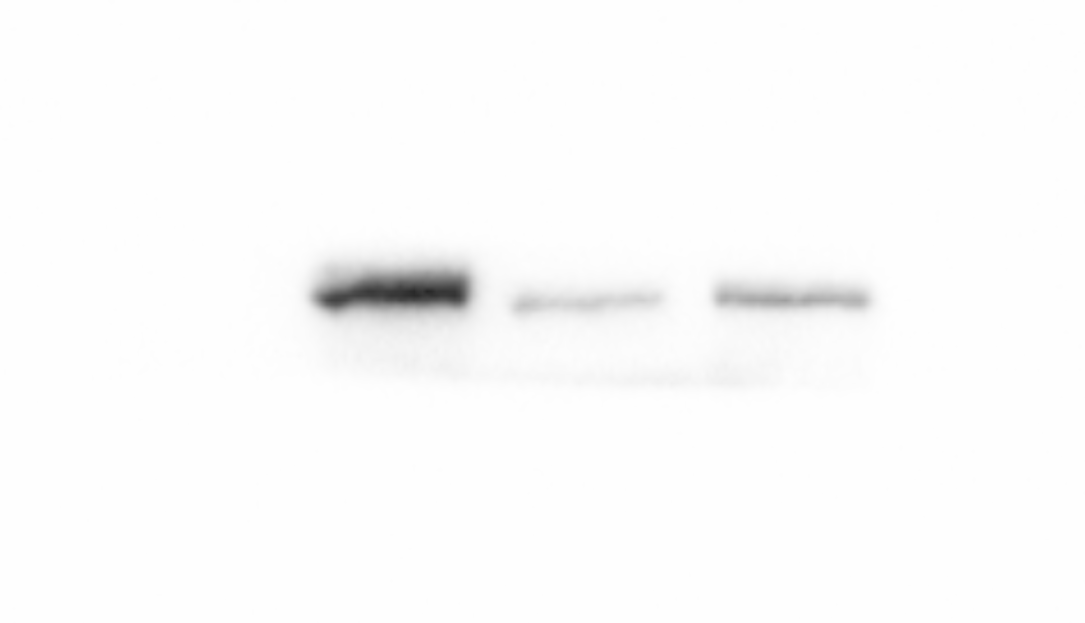

Supplement: Supplementary file 3 [file DataSheet8.zip › WB bands for each gene listed in the article/1、AGIGHC/STAT1-1.tif]

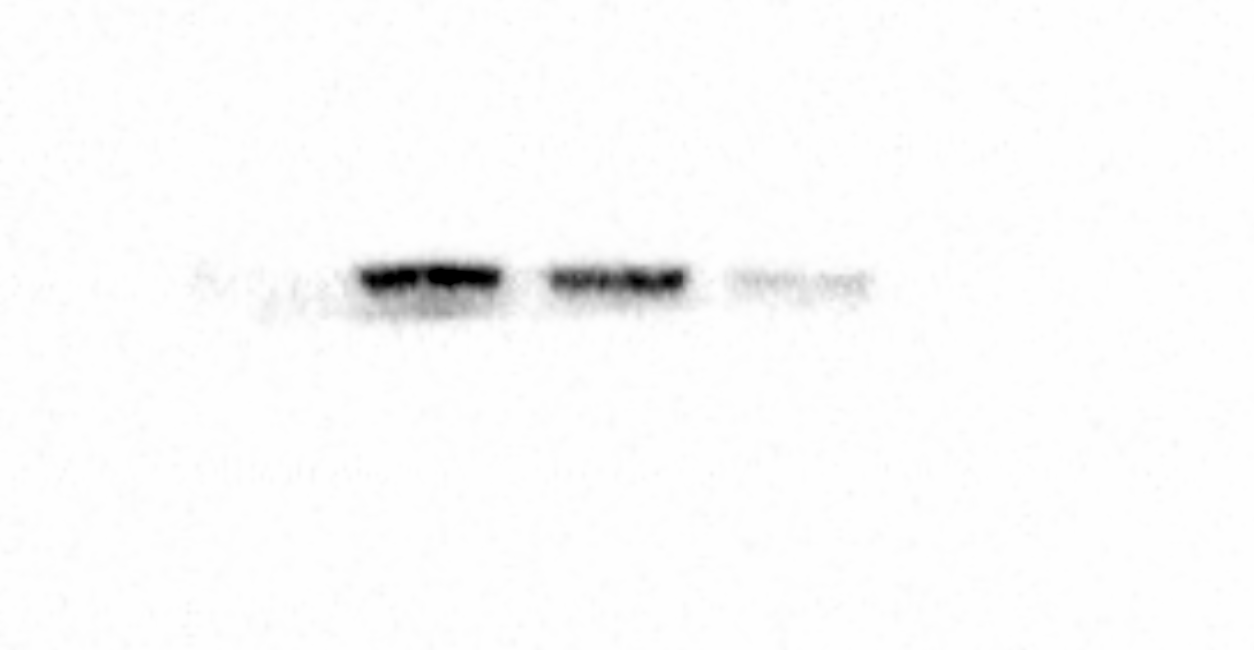

Supplement: Supplementary file 3 [file DataSheet8.zip › WB bands for each gene listed in the article/1、AGIGHC/STAT3-1.tif]

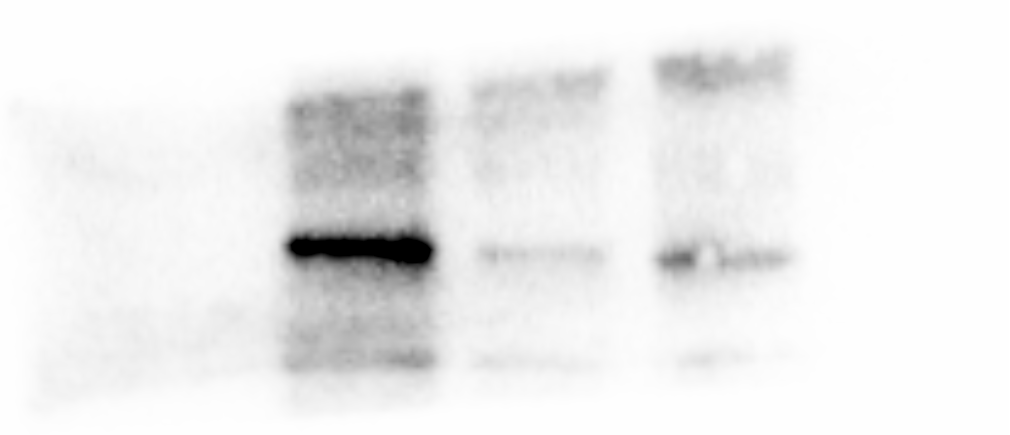

Supplement: Supplementary file 3 [file DataSheet8.zip › WB bands for each gene listed in the article/1、AGIGHC/p-JAK2-1.tif]

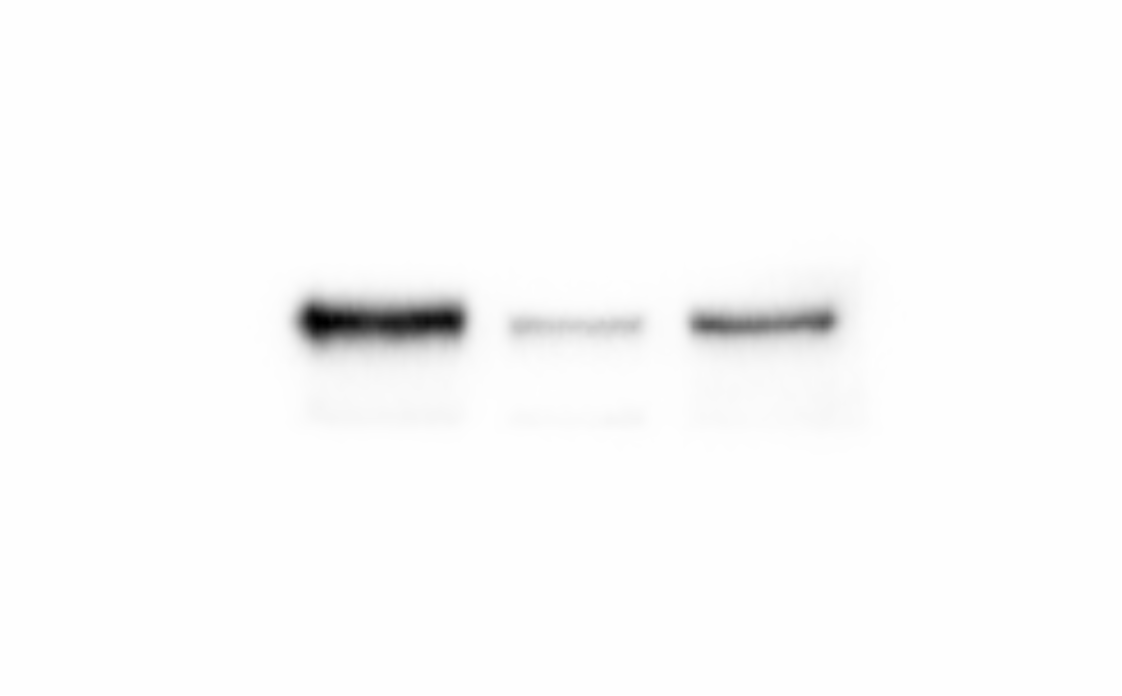

Supplement: Supplementary file 3 [file DataSheet8.zip › WB bands for each gene listed in the article/1、AGIGHC/p-STAT1-1.tif]

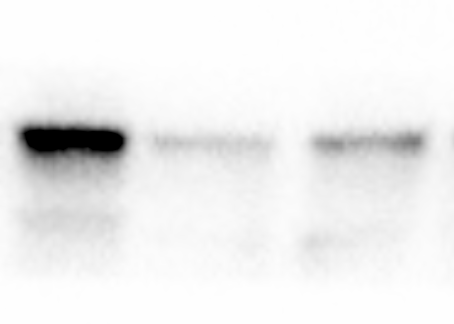

Supplement: Supplementary file 3 [file DataSheet8.zip › WB bands for each gene listed in the article/1、AGIGHC/p-STAT3-1.tif]

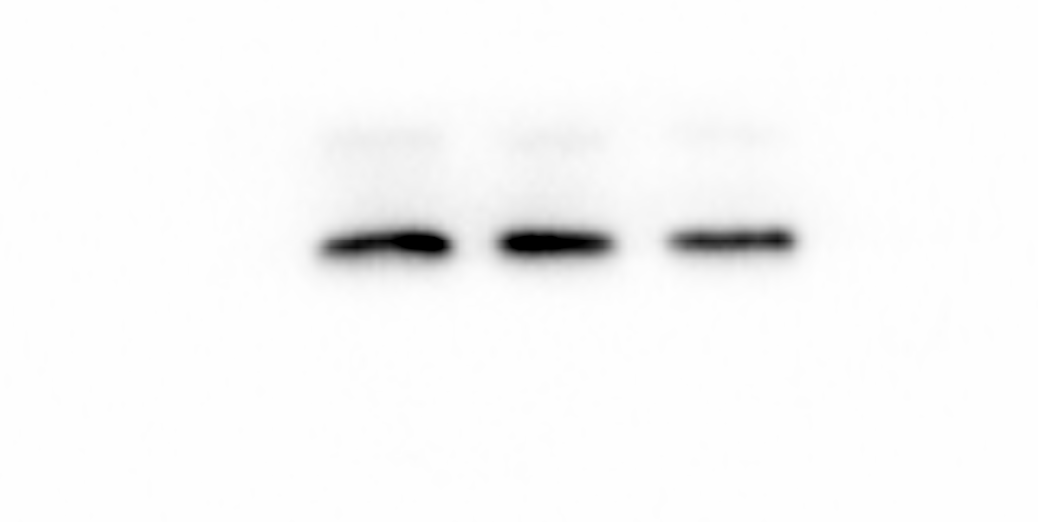

Supplement: Supplementary file 3 [file DataSheet8.zip › WB bands for each gene listed in the article/2、2h WB bands in an in vitro inflammatory model of human blood gout/GAPDH-1.tif]

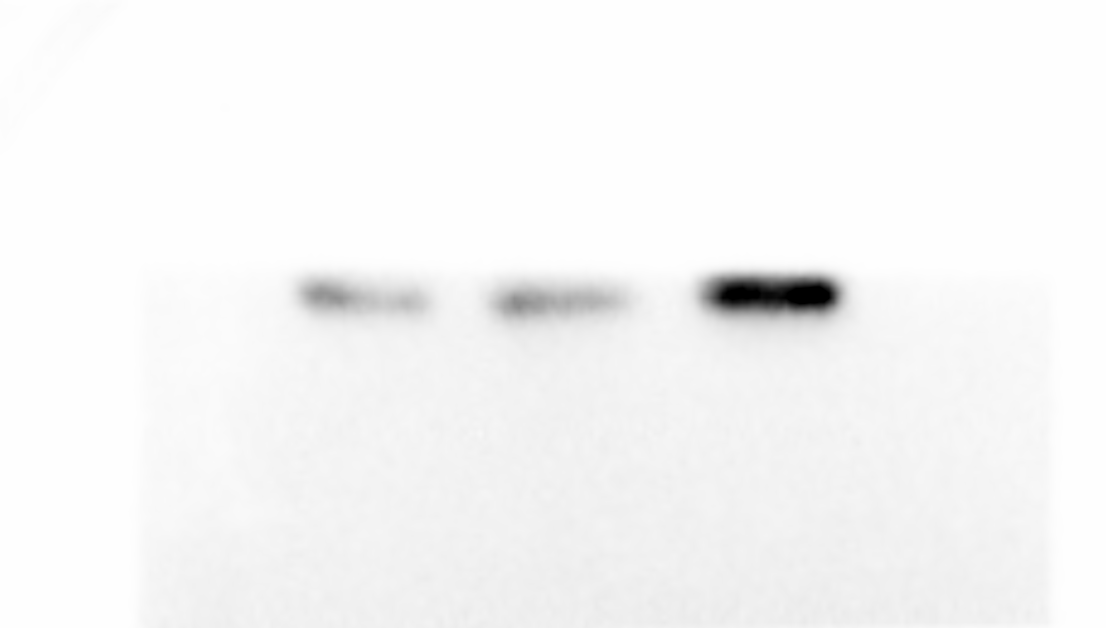

Supplement: Supplementary file 3 [file DataSheet8.zip › WB bands for each gene listed in the article/2、2h WB bands in an in vitro inflammatory model of human blood gout/IL-1β-1.tif]

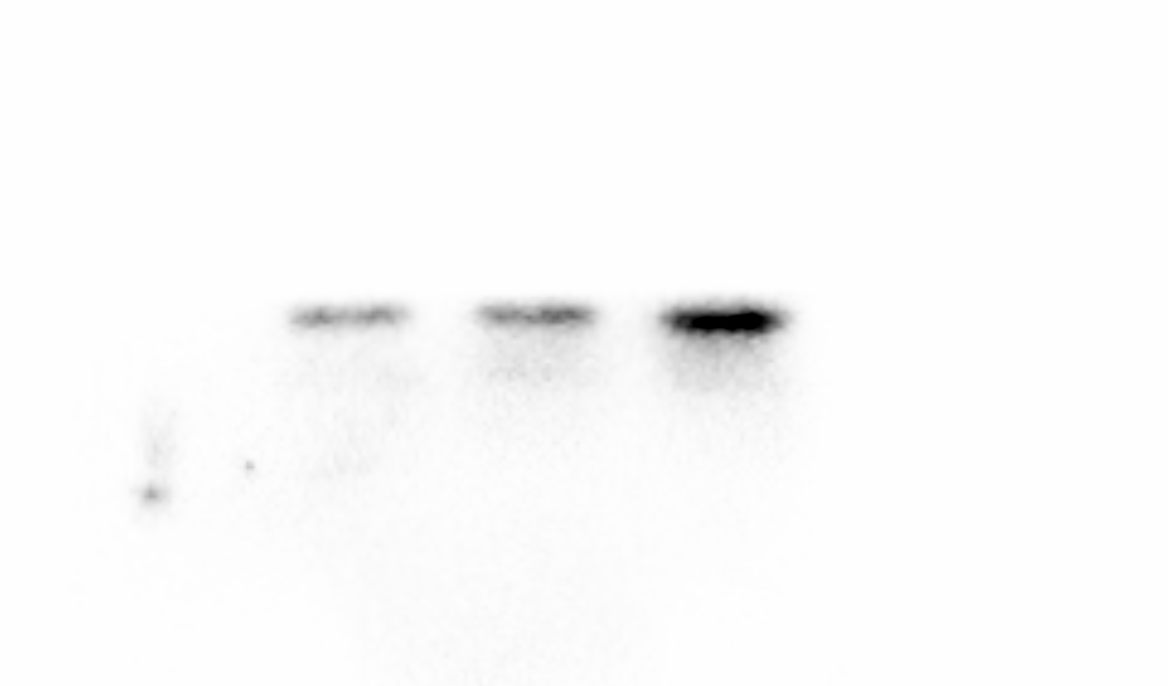

Supplement: Supplementary file 3 [file DataSheet8.zip › WB bands for each gene listed in the article/2、2h WB bands in an in vitro inflammatory model of human blood gout/IL6-1.tif]

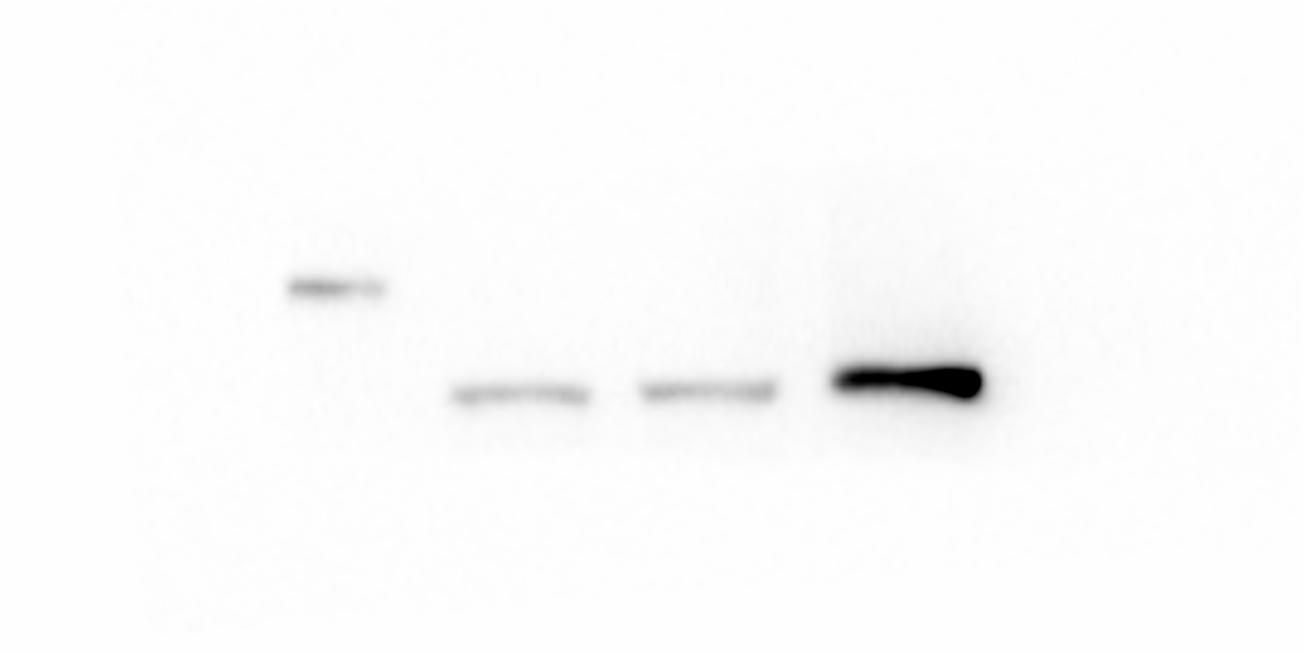

Supplement: Supplementary file 3 [file DataSheet8.zip › WB bands for each gene listed in the article/2、2h WB bands in an in vitro inflammatory model of human blood gout/JAK2-1.tif]

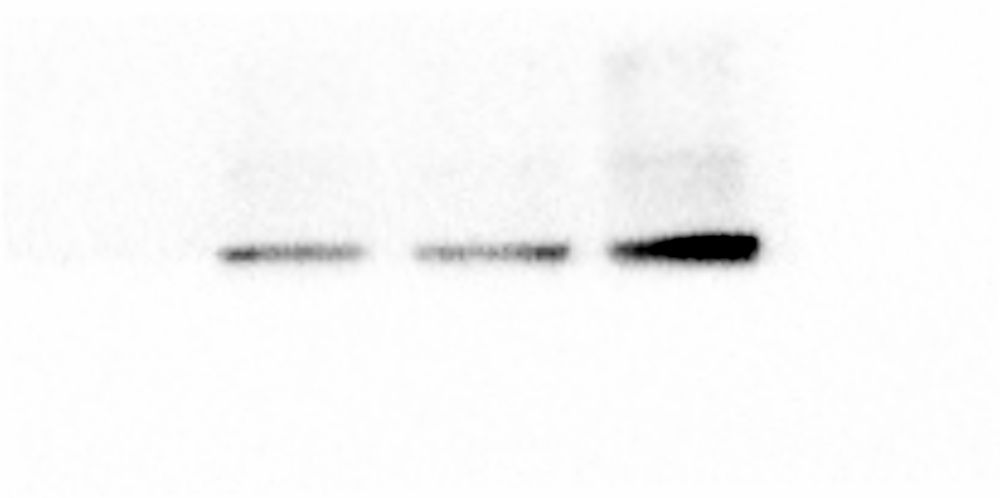

Supplement: Supplementary file 3 [file DataSheet8.zip › WB bands for each gene listed in the article/2、2h WB bands in an in vitro inflammatory model of human blood gout/STAT1-1.tif]

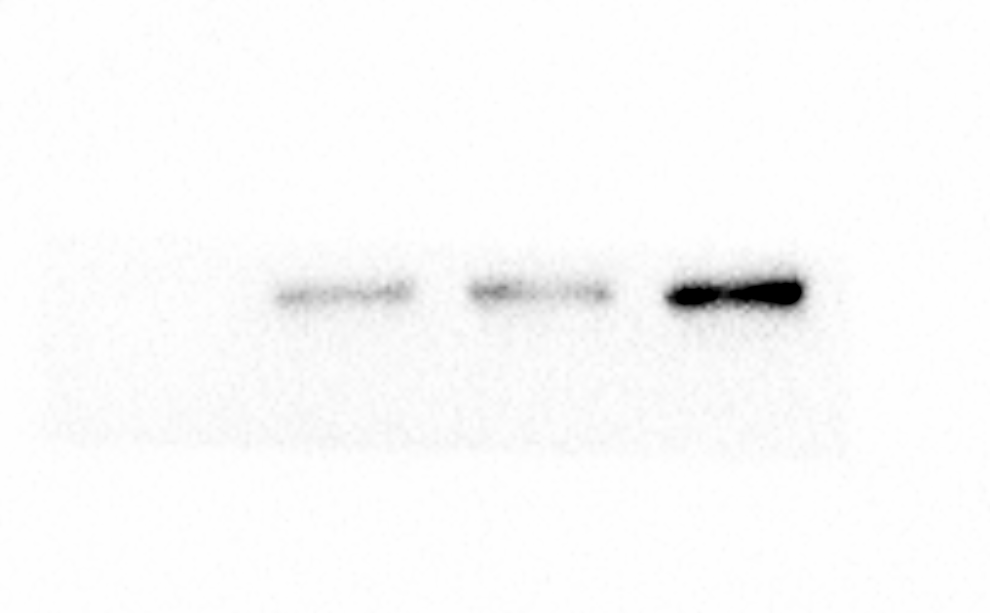

Supplement: Supplementary file 3 [file DataSheet8.zip › WB bands for each gene listed in the article/2、2h WB bands in an in vitro inflammatory model of human blood gout/STAT3-1.tif]

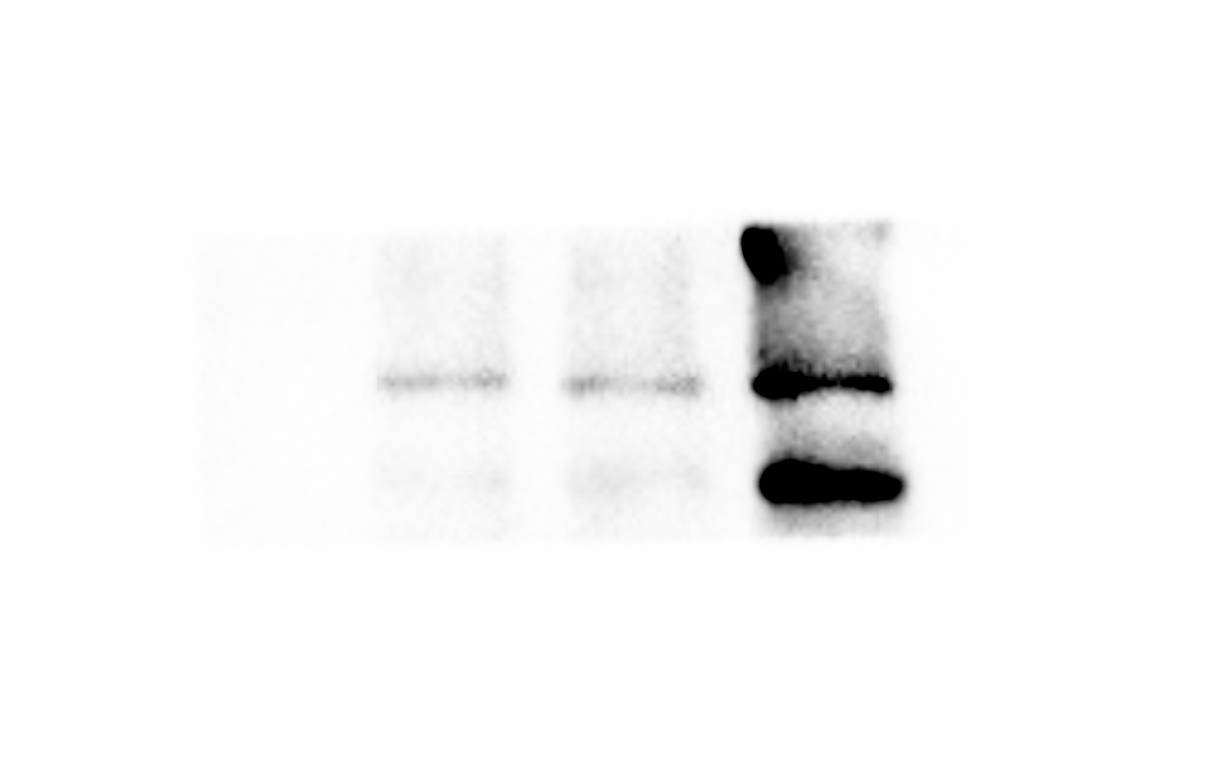

Supplement: Supplementary file 3 [file DataSheet8.zip › WB bands for each gene listed in the article/2、2h WB bands in an in vitro inflammatory model of human blood gout/p-JAK2-1.tif]

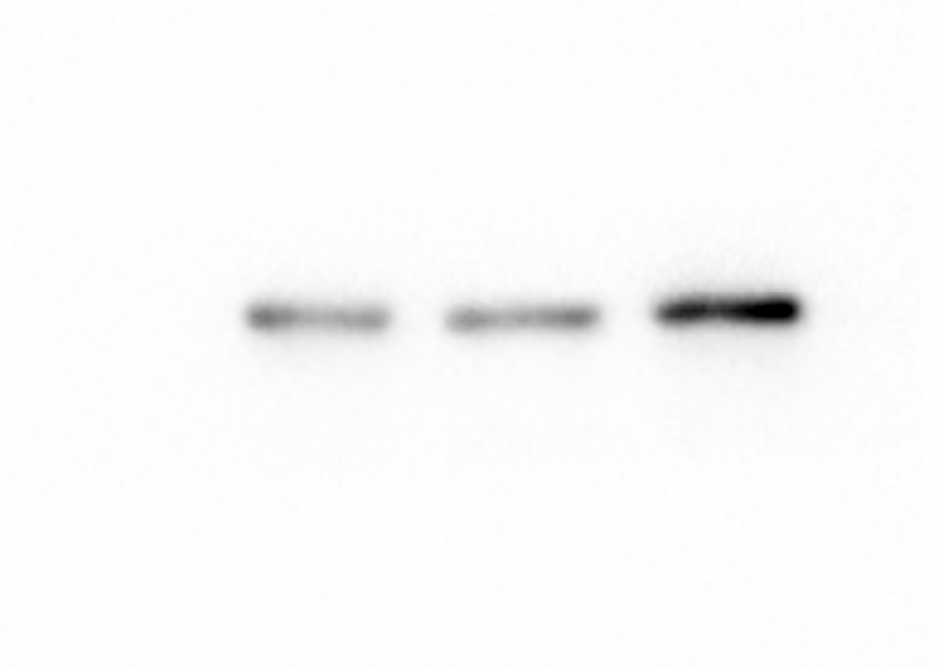

Supplement: Supplementary file 3 [file DataSheet8.zip › WB bands for each gene listed in the article/2、2h WB bands in an in vitro inflammatory model of human blood gout/p-STAT1-1.tif]

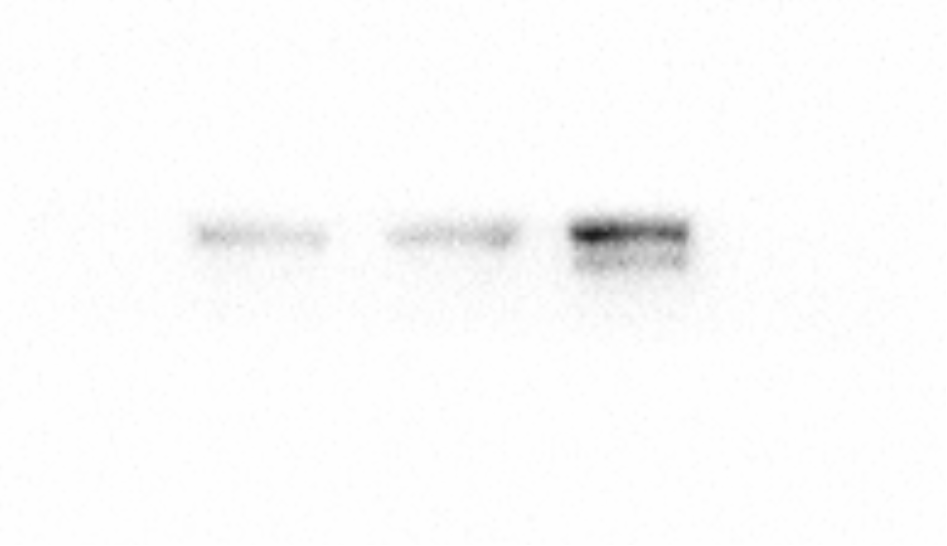

Supplement: Supplementary file 3 [file DataSheet8.zip › WB bands for each gene listed in the article/2、2h WB bands in an in vitro inflammatory model of human blood gout/p-STAT3-1.tif]

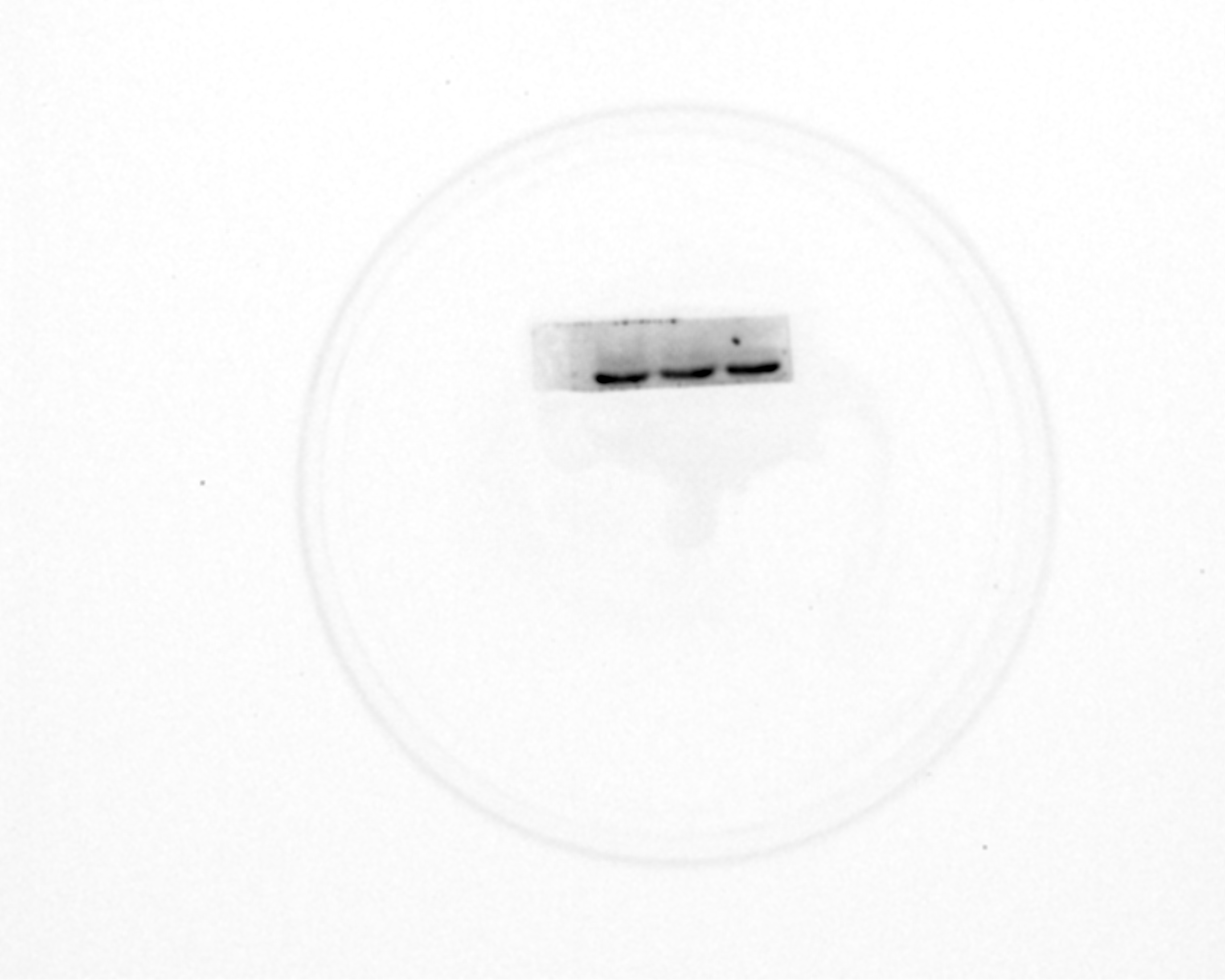

Supplement: Supplementary file 3 [file DataSheet8.zip › WB bands for each gene listed in the article/3、WB bands from THP-1 cell gout model/GAPDH-1-1.tif]

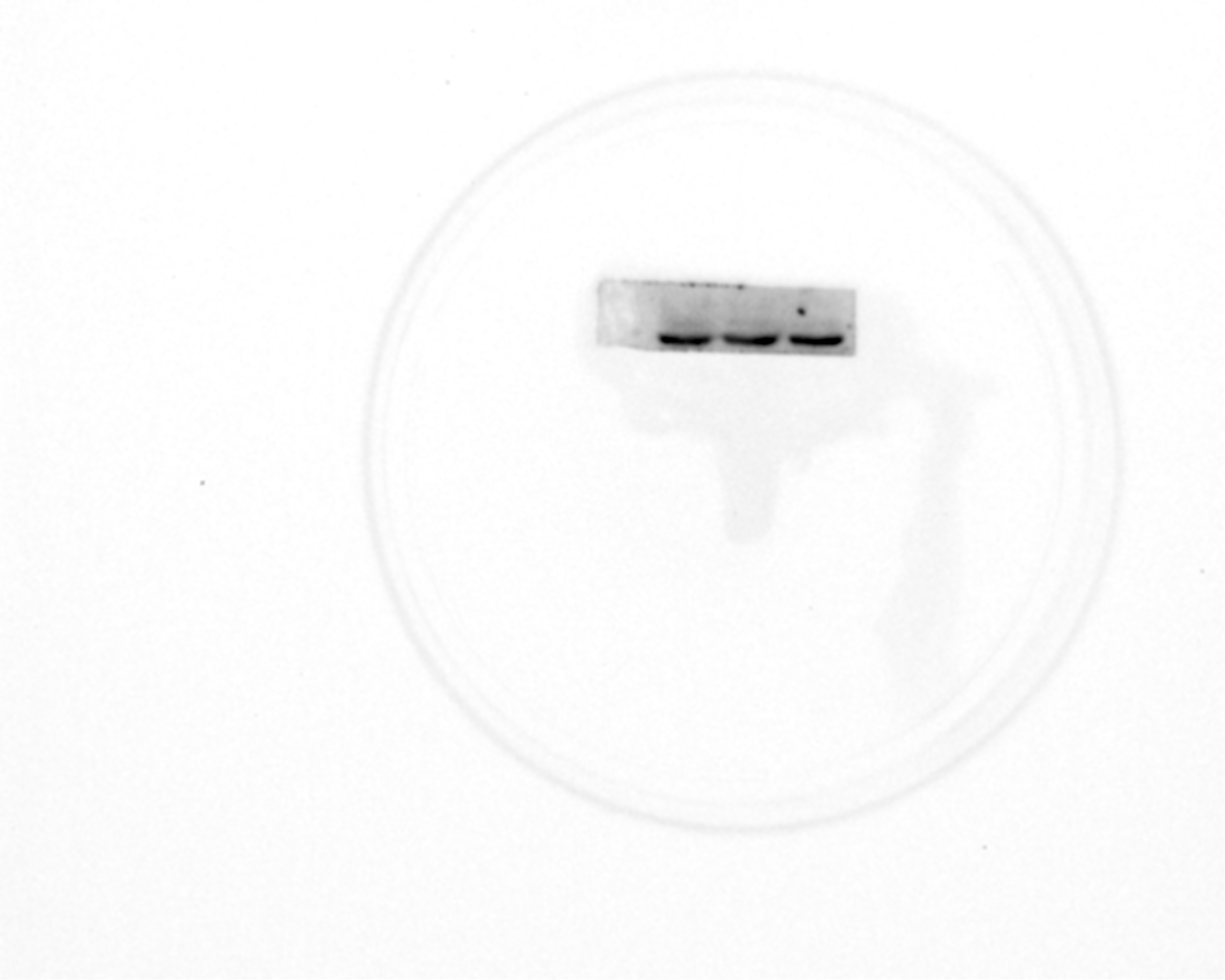

Supplement: Supplementary file 3 [file DataSheet8.zip › WB bands for each gene listed in the article/3、WB bands from THP-1 cell gout model/GAPDH-1.tif]

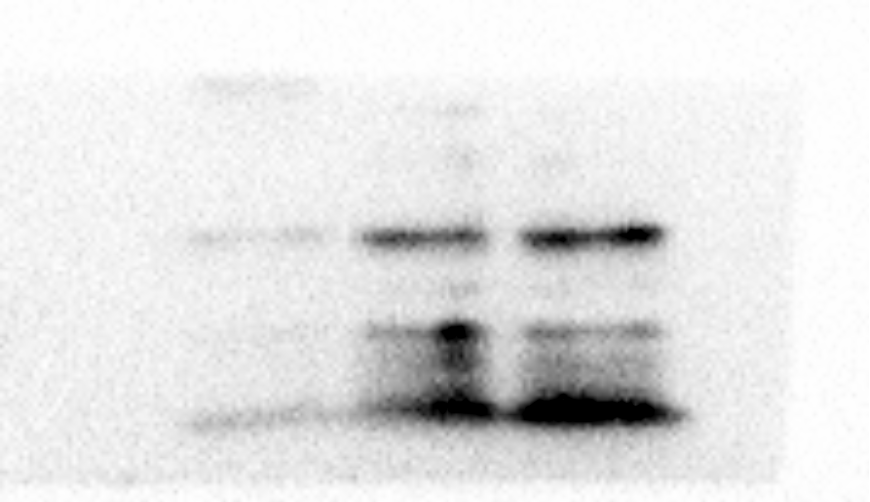

Supplement: Supplementary file 3 [file DataSheet8.zip › WB bands for each gene listed in the article/3、WB bands from THP-1 cell gout model/IL-1β-1.tif]

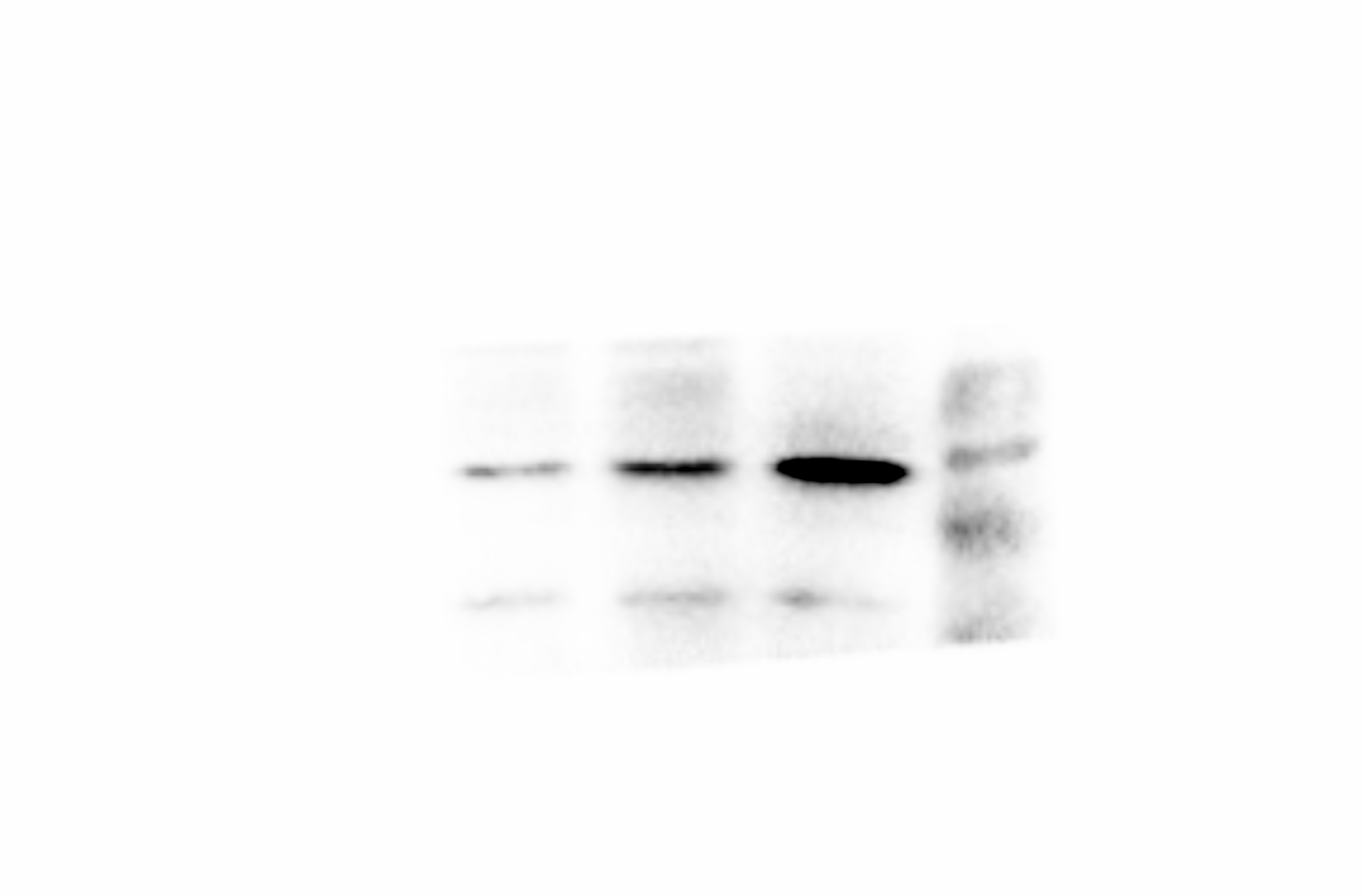

Supplement: Supplementary file 3 [file DataSheet8.zip › WB bands for each gene listed in the article/3、WB bands from THP-1 cell gout model/IL6-1-1.tif]

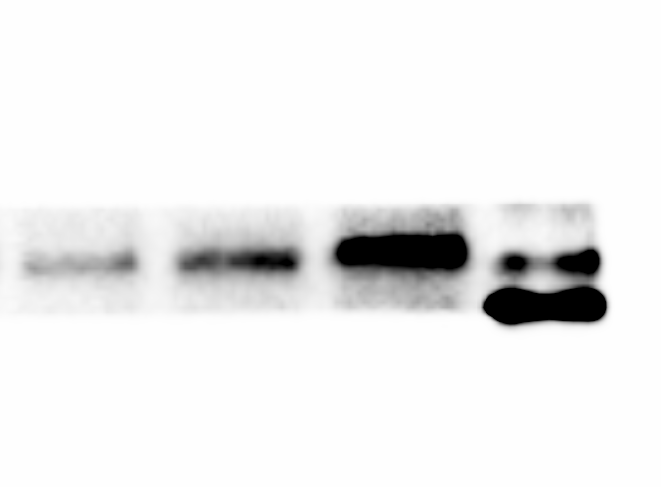

Supplement: Supplementary file 3 [file DataSheet8.zip › WB bands for each gene listed in the article/3、WB bands from THP-1 cell gout model/JAK2-1.tif]

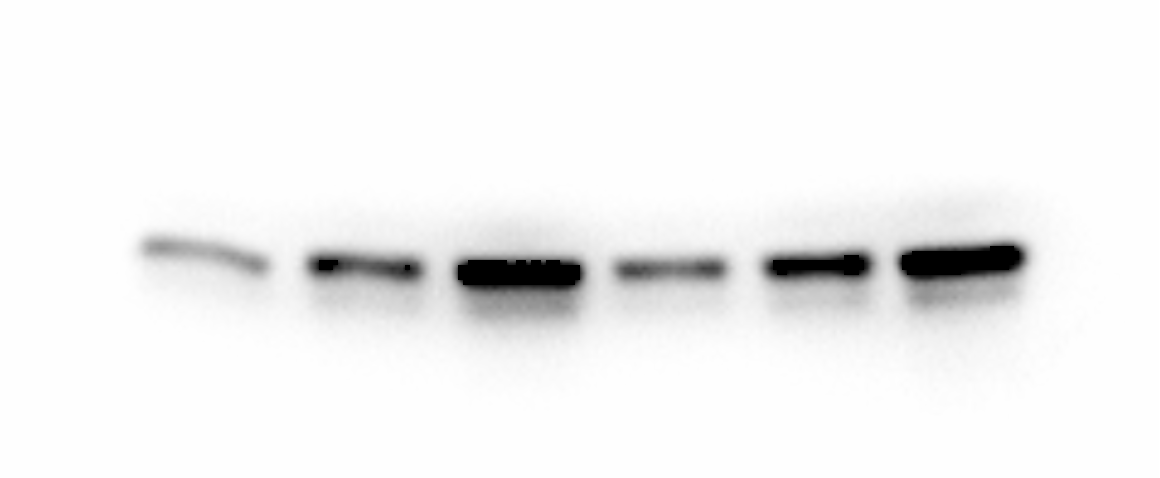

Supplement: Supplementary file 3 [file DataSheet8.zip › WB bands for each gene listed in the article/3、WB bands from THP-1 cell gout model/STAT1-1.tif]

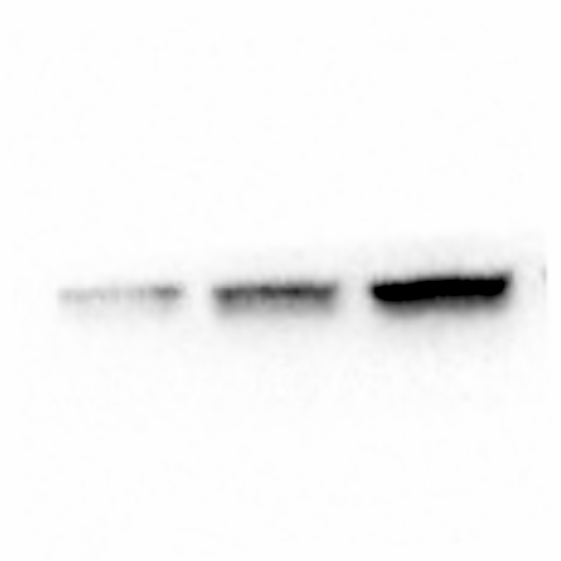

Supplement: Supplementary file 3 [file DataSheet8.zip › WB bands for each gene listed in the article/3、WB bands from THP-1 cell gout model/STAT3-1.tif]

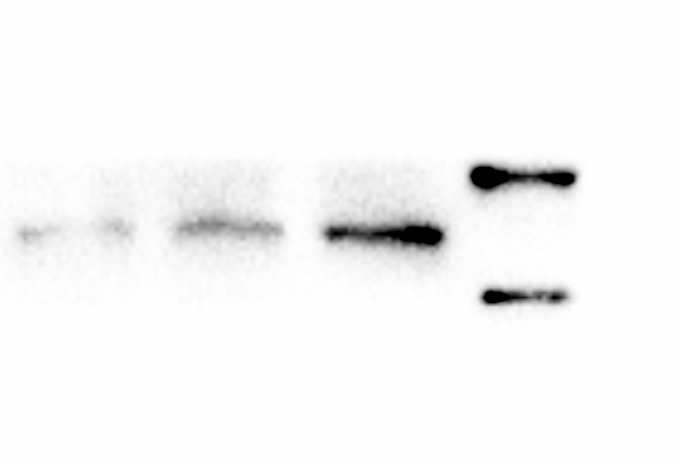

Supplement: Supplementary file 3 [file DataSheet8.zip › WB bands for each gene listed in the article/3、WB bands from THP-1 cell gout model/p-JAK2-1.tif]

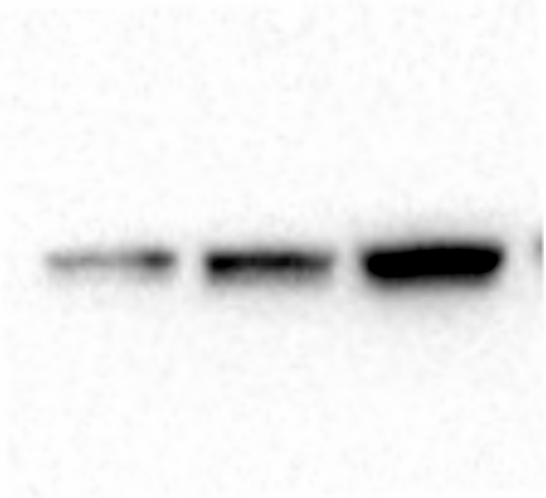

Supplement: Supplementary file 3 [file DataSheet8.zip › WB bands for each gene listed in the article/3、WB bands from THP-1 cell gout model/p-STAT1-1.tif]

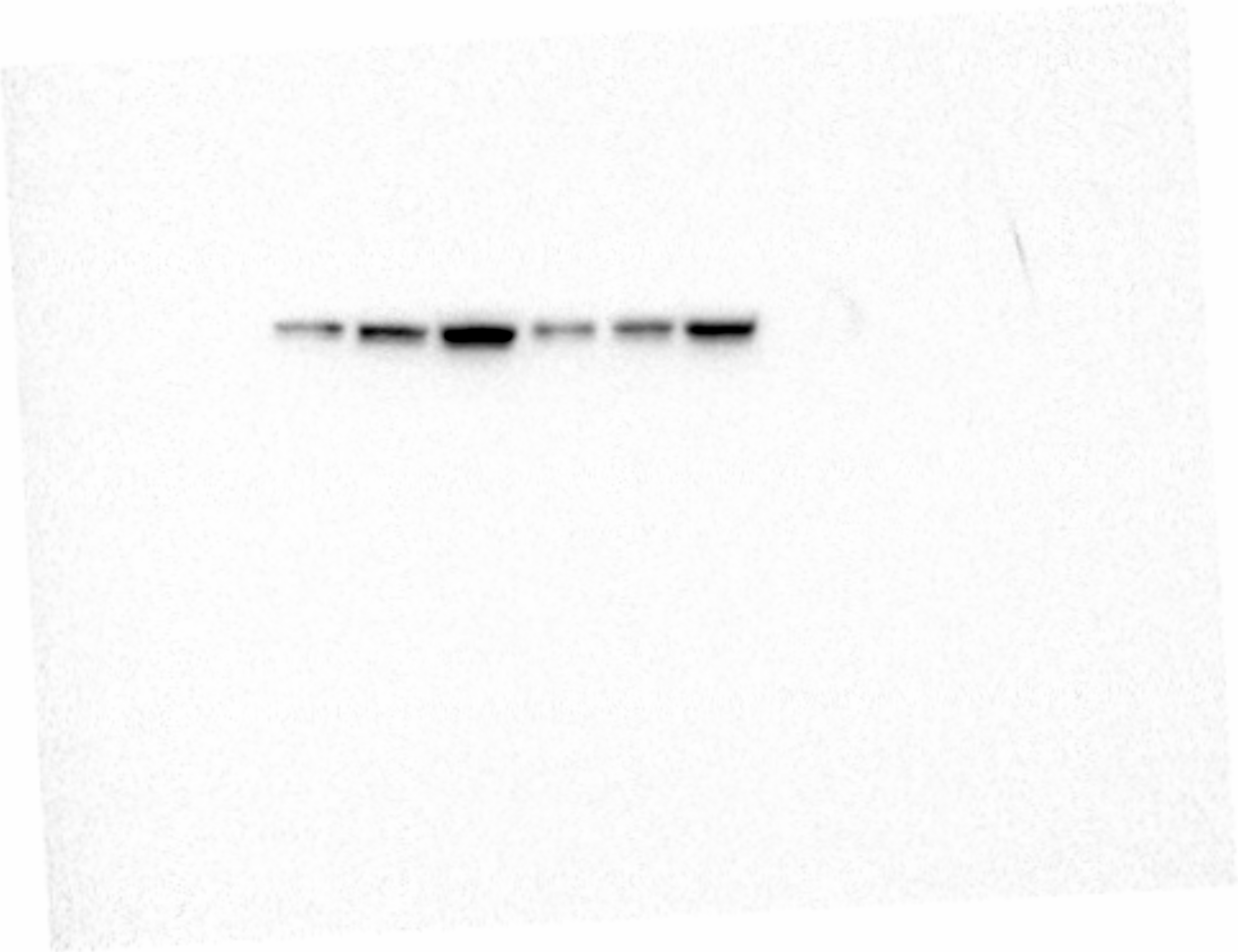

Supplement: Supplementary file 3 [file DataSheet8.zip › WB bands for each gene listed in the article/3、WB bands from THP-1 cell gout model/p-STAT3-1.tif]

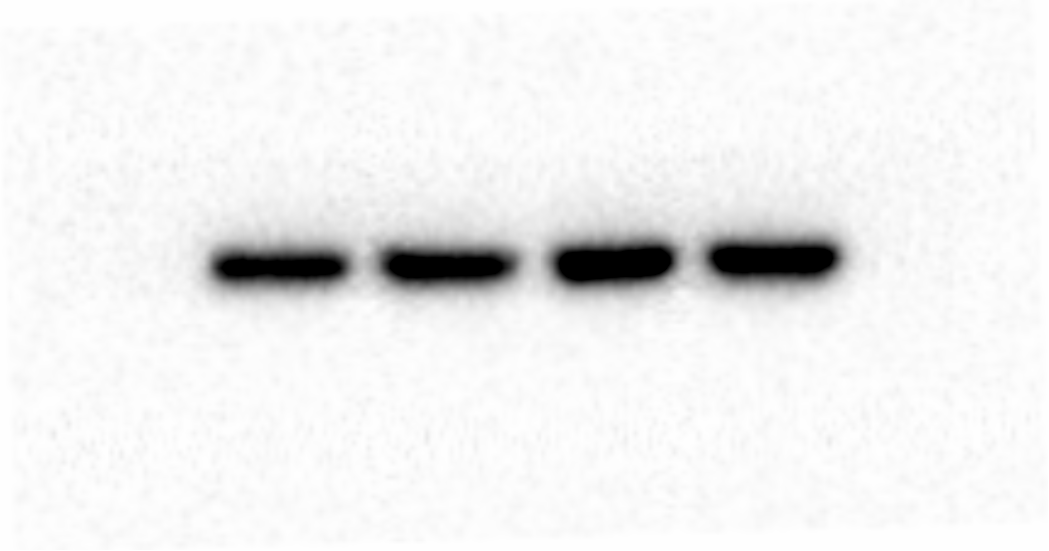

Supplement: Supplementary file 3 [file DataSheet8.zip › WB bands for each gene listed in the article/4、IL-6 knockout mouse and WT mouse WB strips/GAPDH-1.tif]

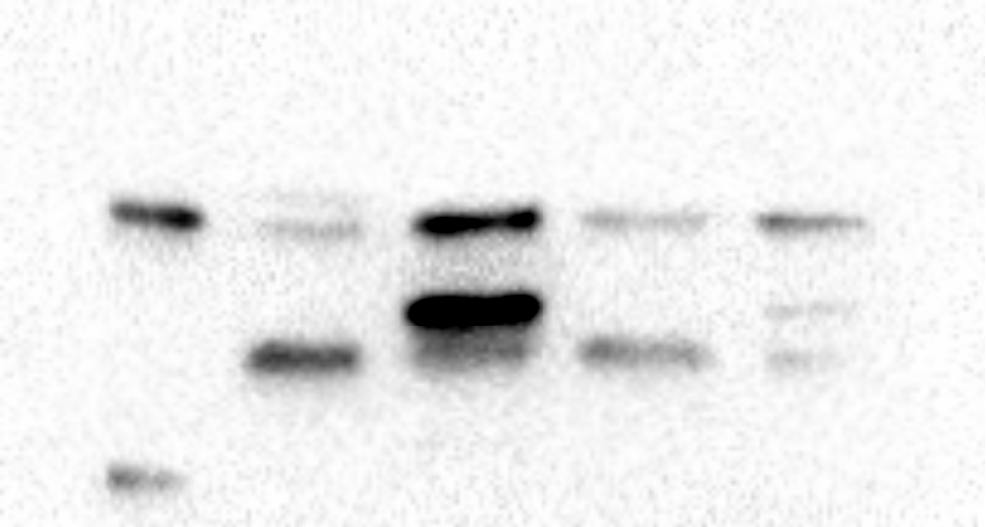

Supplement: Supplementary file 3 [file DataSheet8.zip › WB bands for each gene listed in the article/4、IL-6 knockout mouse and WT mouse WB strips/IL-1β-1.tif]

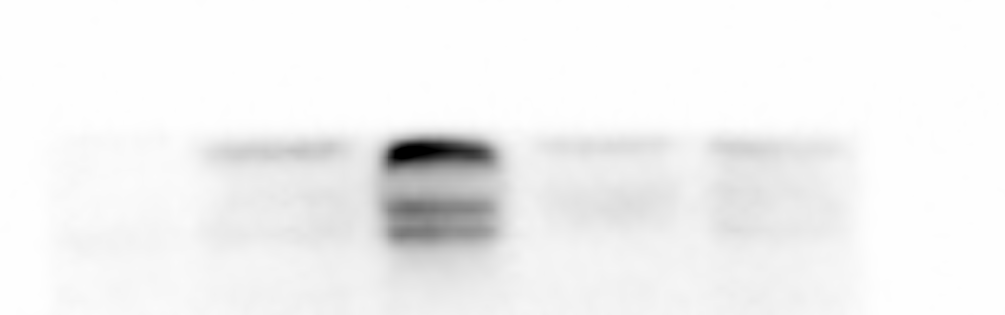

Supplement: Supplementary file 3 [file DataSheet8.zip › WB bands for each gene listed in the article/4、IL-6 knockout mouse and WT mouse WB strips/IL6-1.tif]

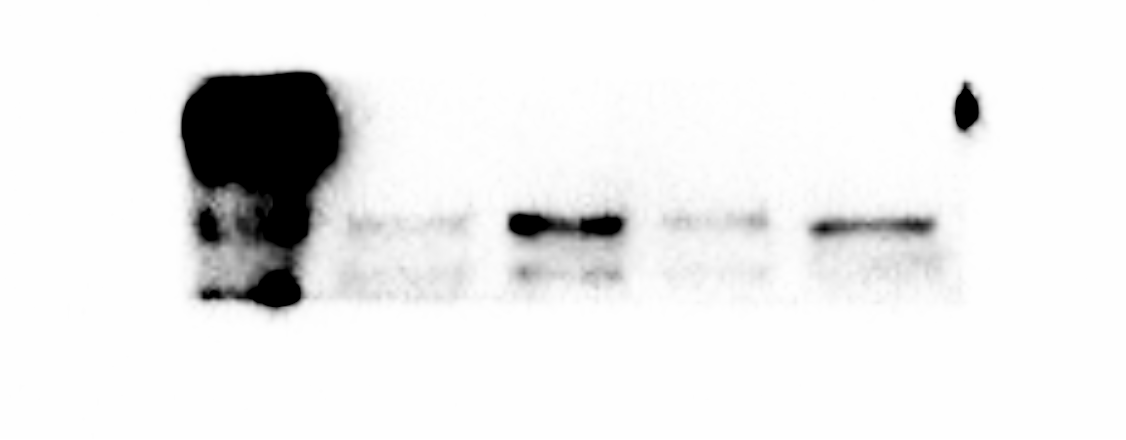

Supplement: Supplementary file 3 [file DataSheet8.zip › WB bands for each gene listed in the article/4、IL-6 knockout mouse and WT mouse WB strips/JAK2-1.tif]

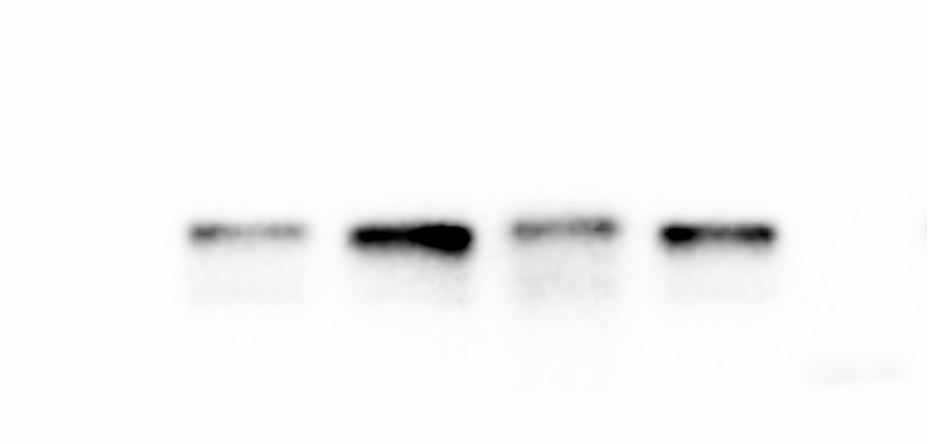

Supplement: Supplementary file 3 [file DataSheet8.zip › WB bands for each gene listed in the article/4、IL-6 knockout mouse and WT mouse WB strips/STAT1-1.tif]

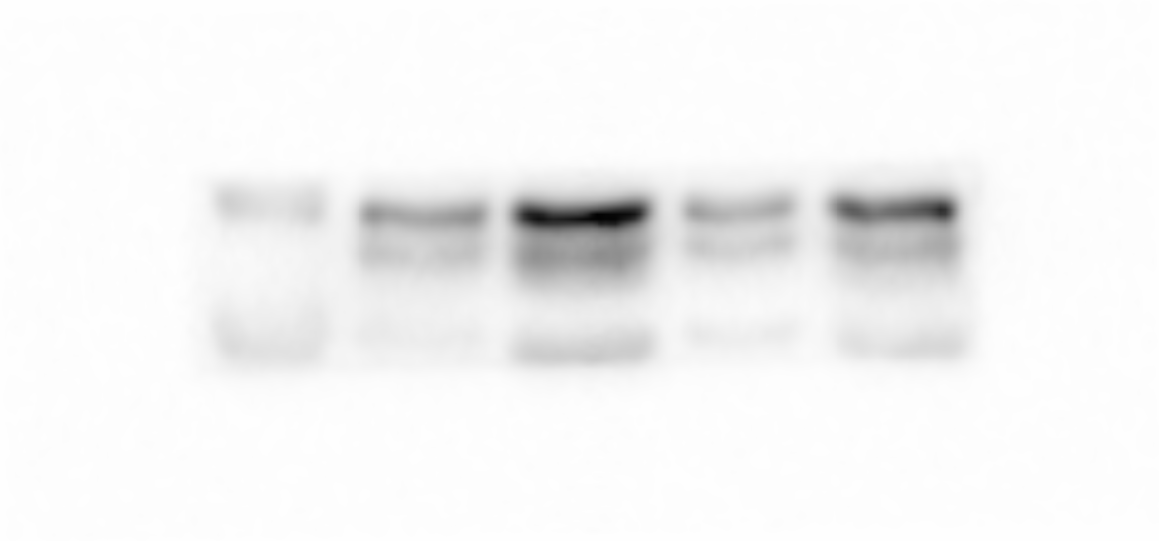

Supplement: Supplementary file 3 [file DataSheet8.zip › WB bands for each gene listed in the article/4、IL-6 knockout mouse and WT mouse WB strips/STAT3-1.tif]

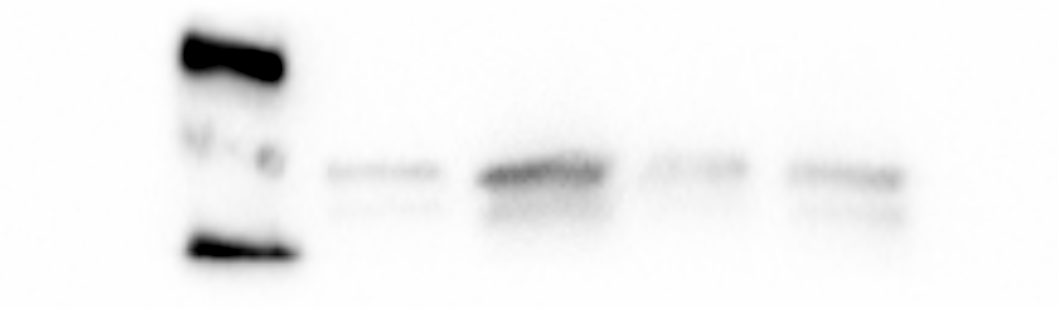

Supplement: Supplementary file 3 [file DataSheet8.zip › WB bands for each gene listed in the article/4、IL-6 knockout mouse and WT mouse WB strips/p-JAK2-1.tif]

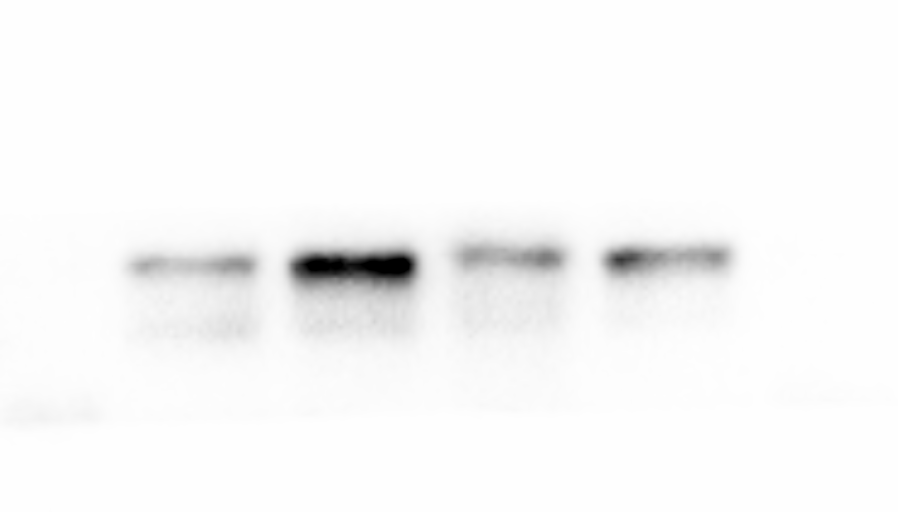

Supplement: Supplementary file 3 [file DataSheet8.zip › WB bands for each gene listed in the article/4、IL-6 knockout mouse and WT mouse WB strips/p-STAT1-1.tif]

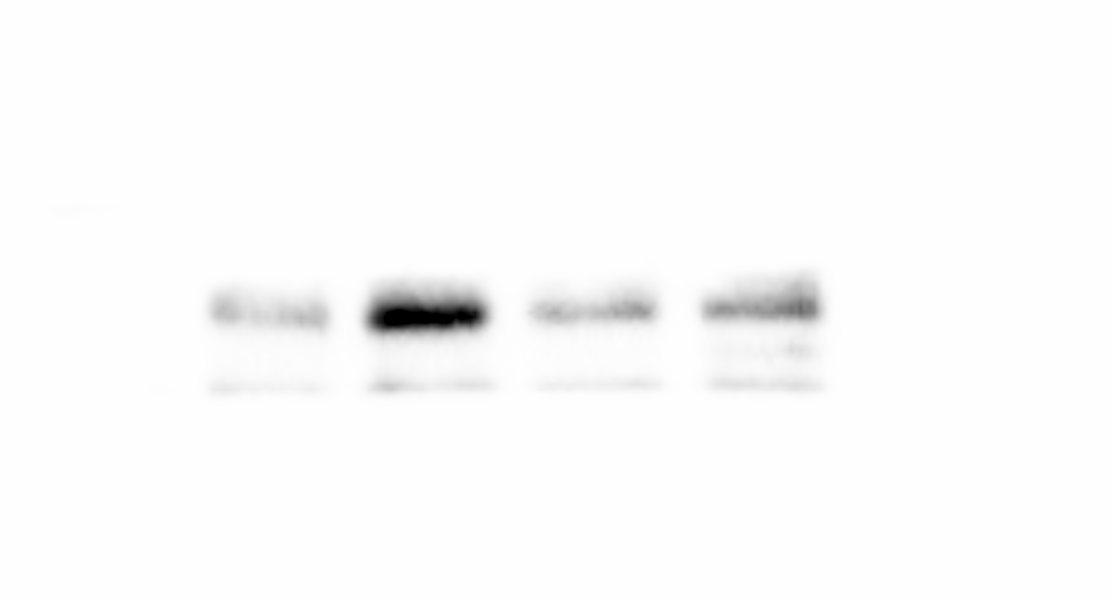

Supplement: Supplementary file 3 [file DataSheet8.zip › WB bands for each gene listed in the article/4、IL-6 knockout mouse and WT mouse WB strips/p-STAT3-1.tif]

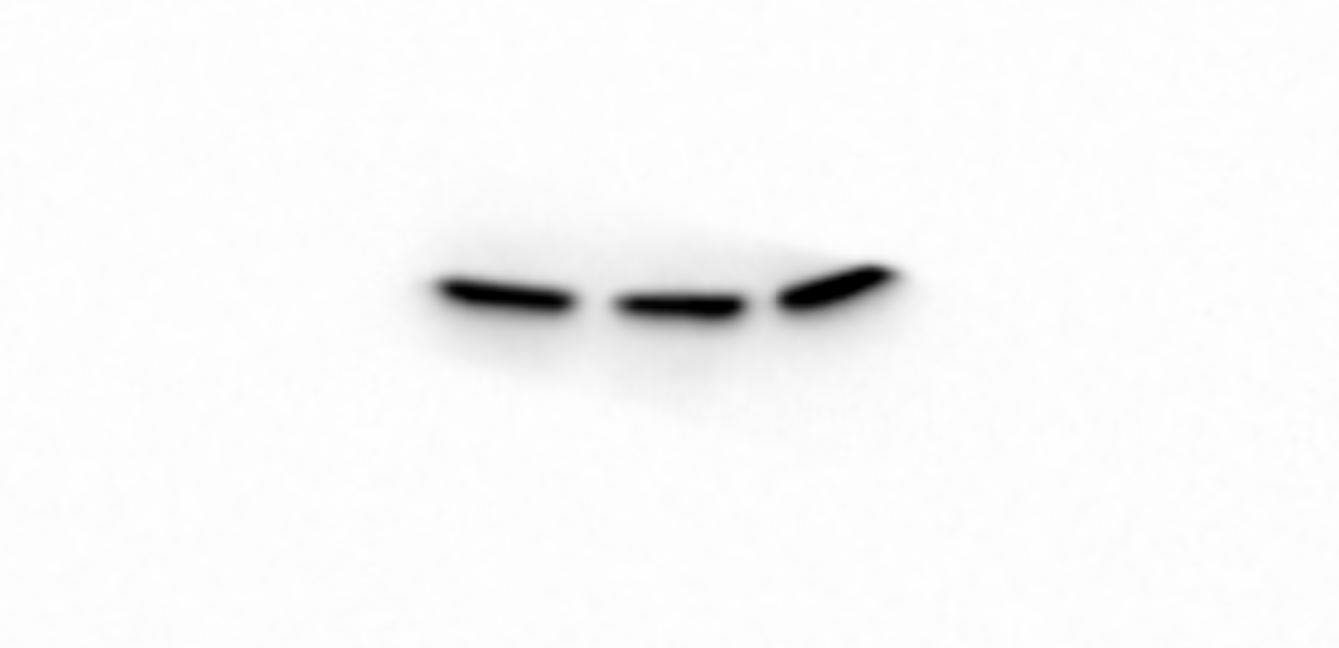

Supplement: Supplementary file 4 [file DataSheet4.zip › 2、 2h WB bands in an in vitro inflammatory model of human blood gout/GAPDH-2.tif]

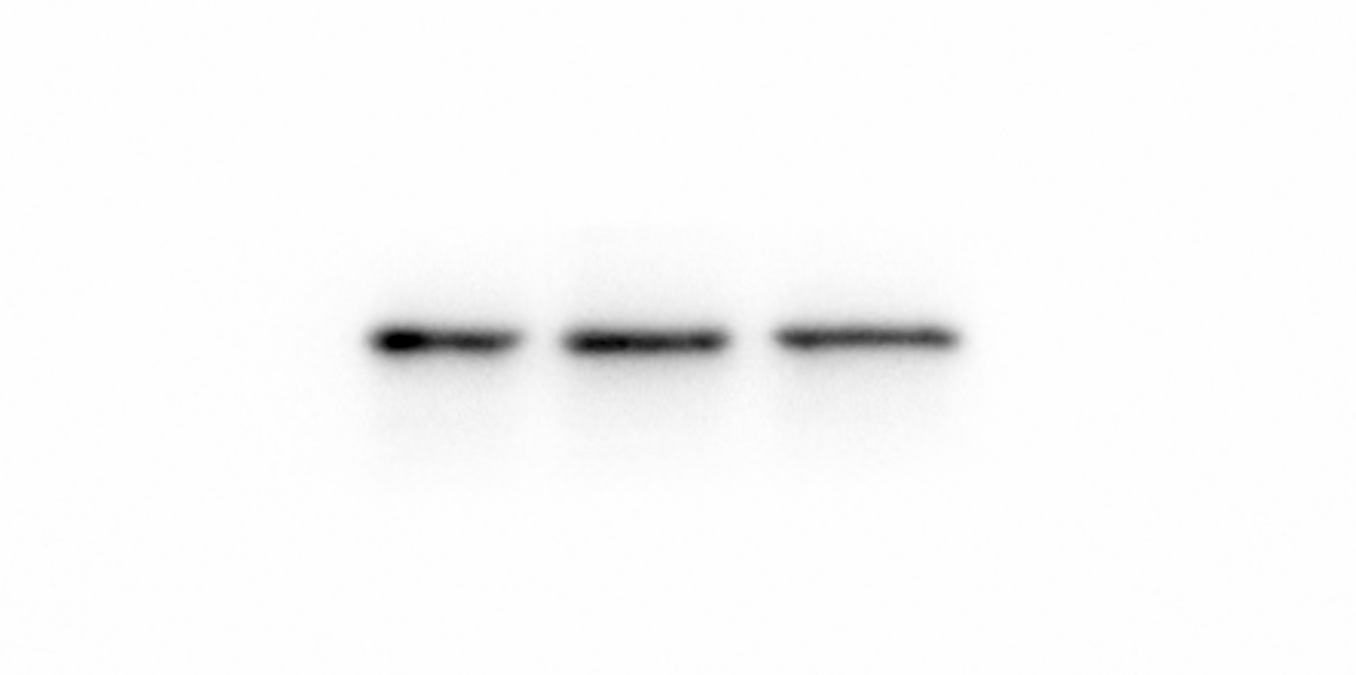

Supplement: Supplementary file 4 [file DataSheet4.zip › 2、 2h WB bands in an in vitro inflammatory model of human blood gout/GAPDH-3.tif]

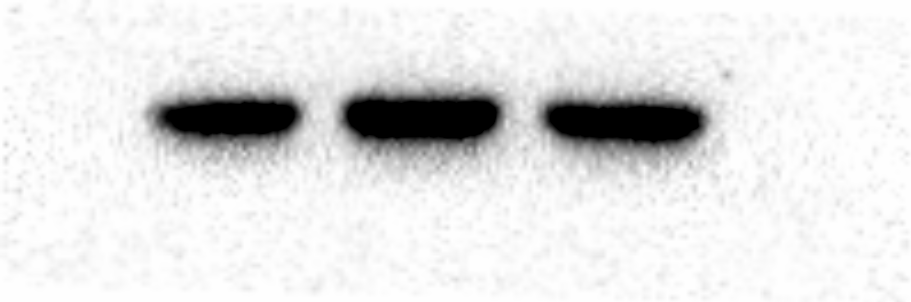

Supplement: Supplementary file 4 [file DataSheet4.zip › 2、 2h WB bands in an in vitro inflammatory model of human blood gout/GAPDH-4.tif]

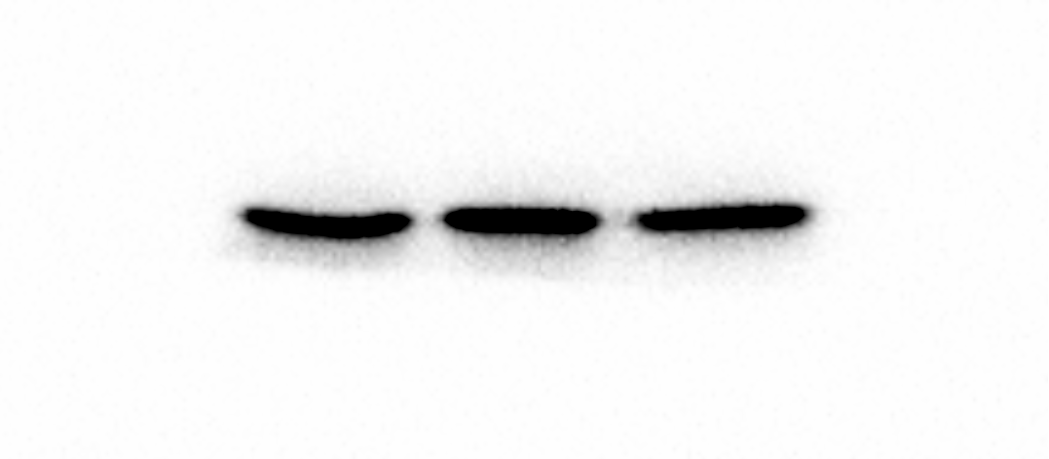

Supplement: Supplementary file 4 [file DataSheet4.zip › 2、 2h WB bands in an in vitro inflammatory model of human blood gout/GAPDH-5.tif]

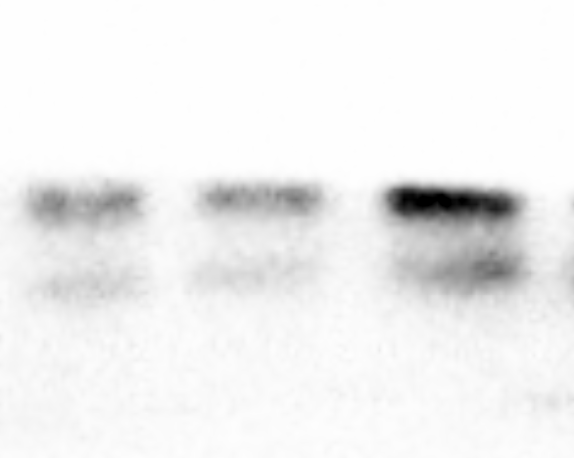

Supplement: Supplementary file 4 [file DataSheet4.zip › 2、 2h WB bands in an in vitro inflammatory model of human blood gout/IL-1β-2.tif]

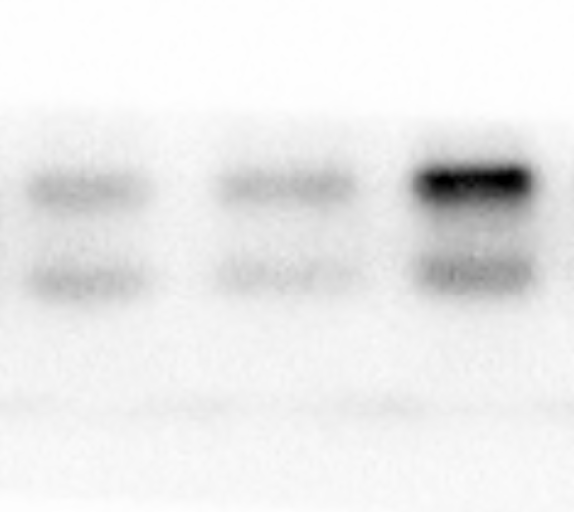

Supplement: Supplementary file 4 [file DataSheet4.zip › 2、 2h WB bands in an in vitro inflammatory model of human blood gout/IL-1β-3.tif]

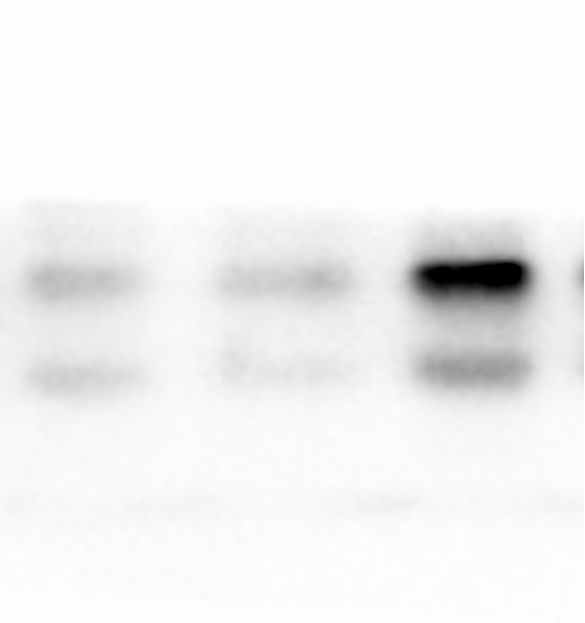

Supplement: Supplementary file 4 [file DataSheet4.zip › 2、 2h WB bands in an in vitro inflammatory model of human blood gout/IL-1β-4.tif]

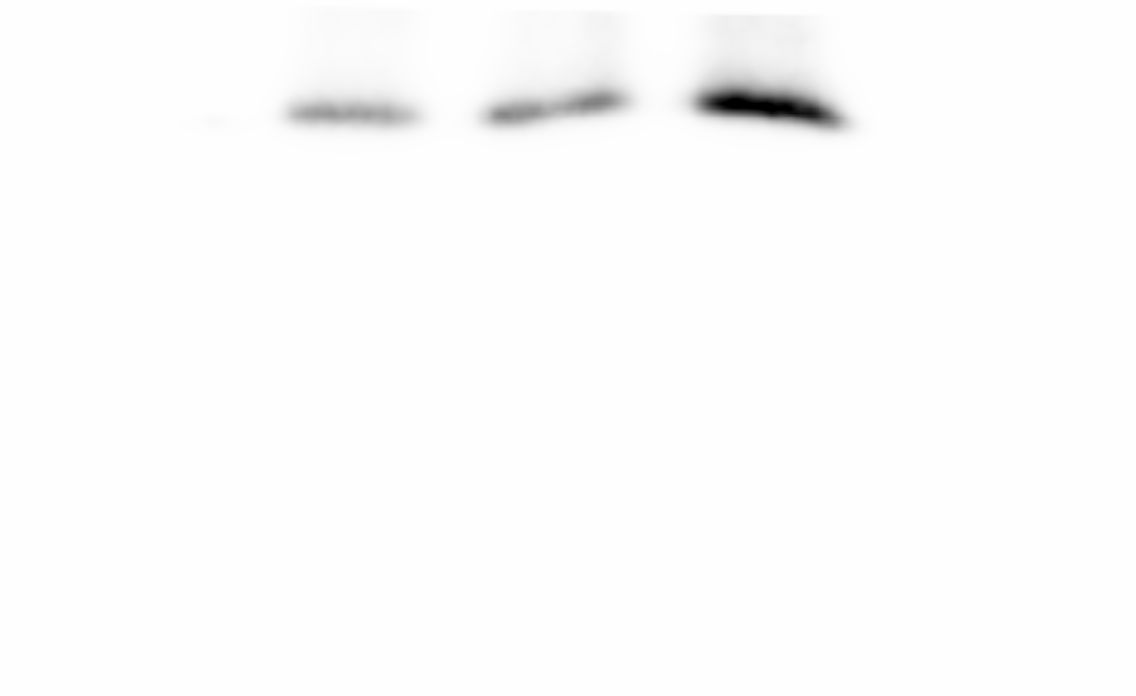

Supplement: Supplementary file 4 [file DataSheet4.zip › 2、 2h WB bands in an in vitro inflammatory model of human blood gout/IL6-2.tif]

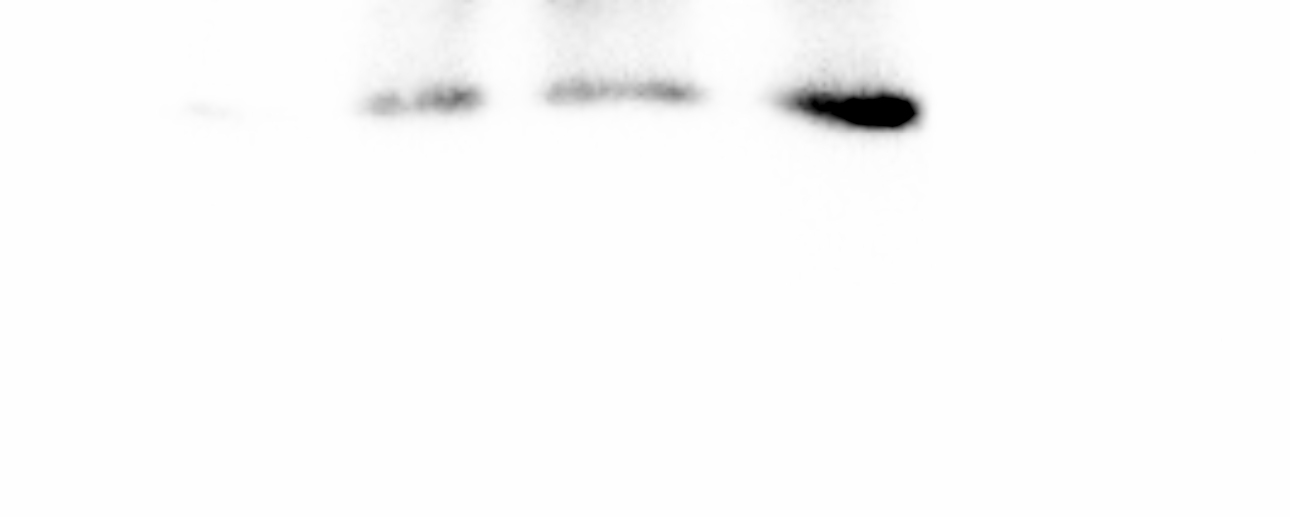

Supplement: Supplementary file 4 [file DataSheet4.zip › 2、 2h WB bands in an in vitro inflammatory model of human blood gout/IL6-3.tif]

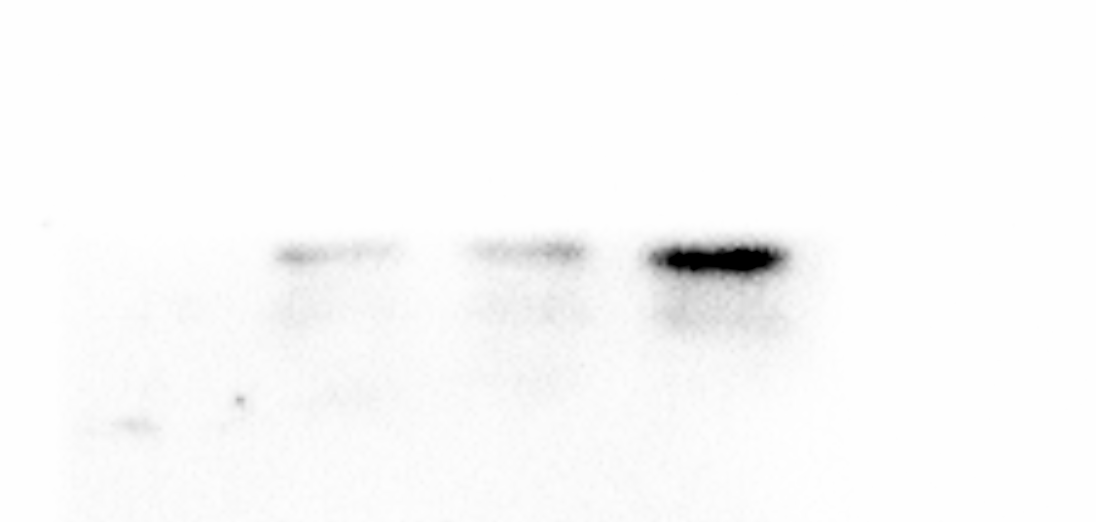

Supplement: Supplementary file 4 [file DataSheet4.zip › 2、 2h WB bands in an in vitro inflammatory model of human blood gout/IL6-4.tif]

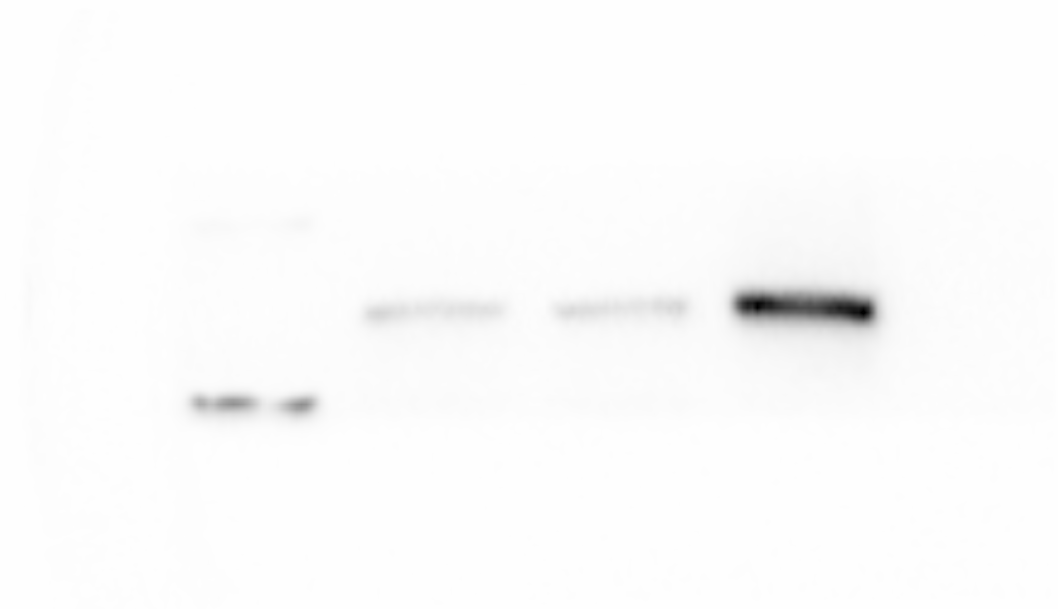

Supplement: Supplementary file 4 [file DataSheet4.zip › 2、 2h WB bands in an in vitro inflammatory model of human blood gout/JAK2-2.tif]

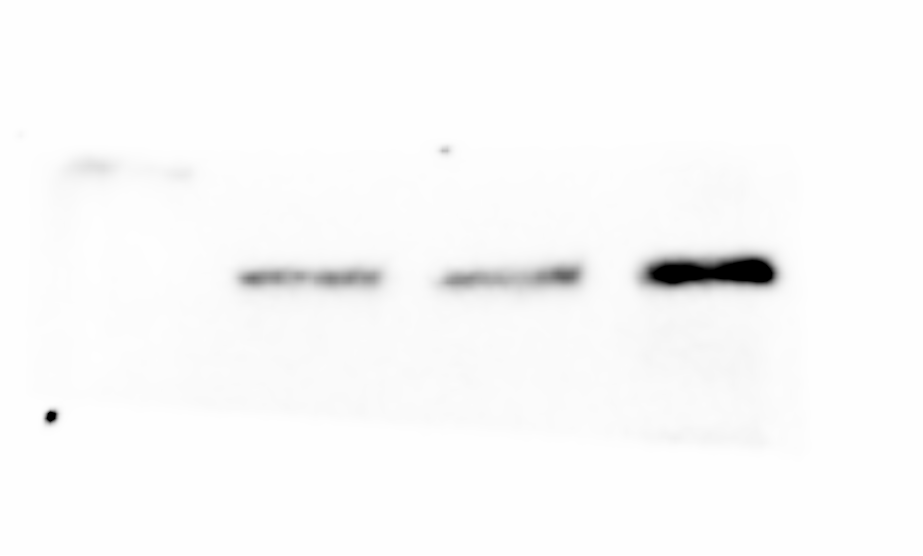

Supplement: Supplementary file 4 [file DataSheet4.zip › 2、 2h WB bands in an in vitro inflammatory model of human blood gout/JAK2-3.tif]

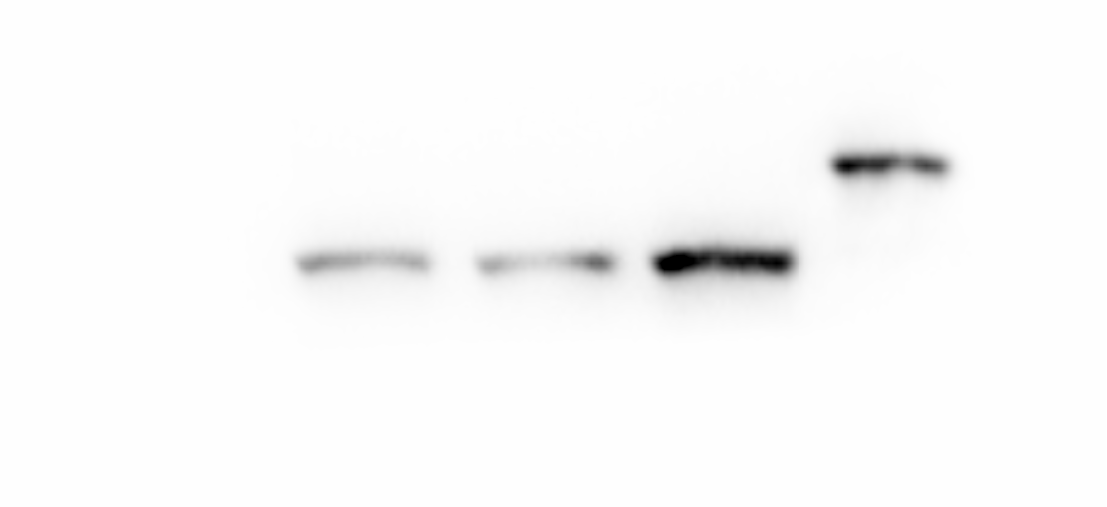

Supplement: Supplementary file 4 [file DataSheet4.zip › 2、 2h WB bands in an in vitro inflammatory model of human blood gout/JAK2-4.tif]

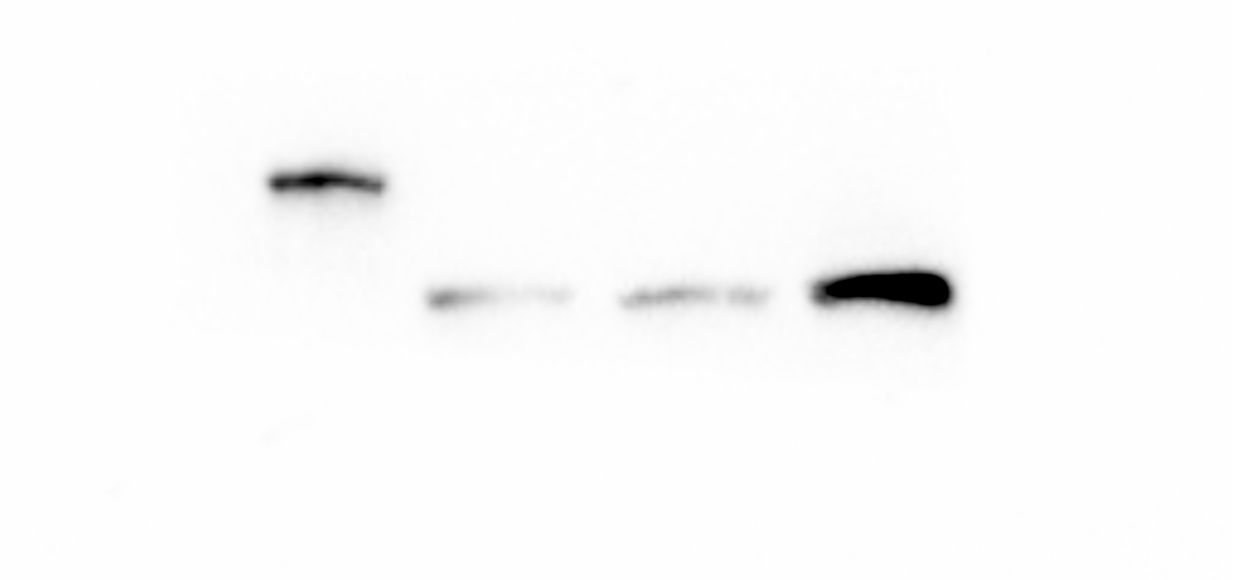

Supplement: Supplementary file 4 [file DataSheet4.zip › 2、 2h WB bands in an in vitro inflammatory model of human blood gout/JAK2-5.tif]

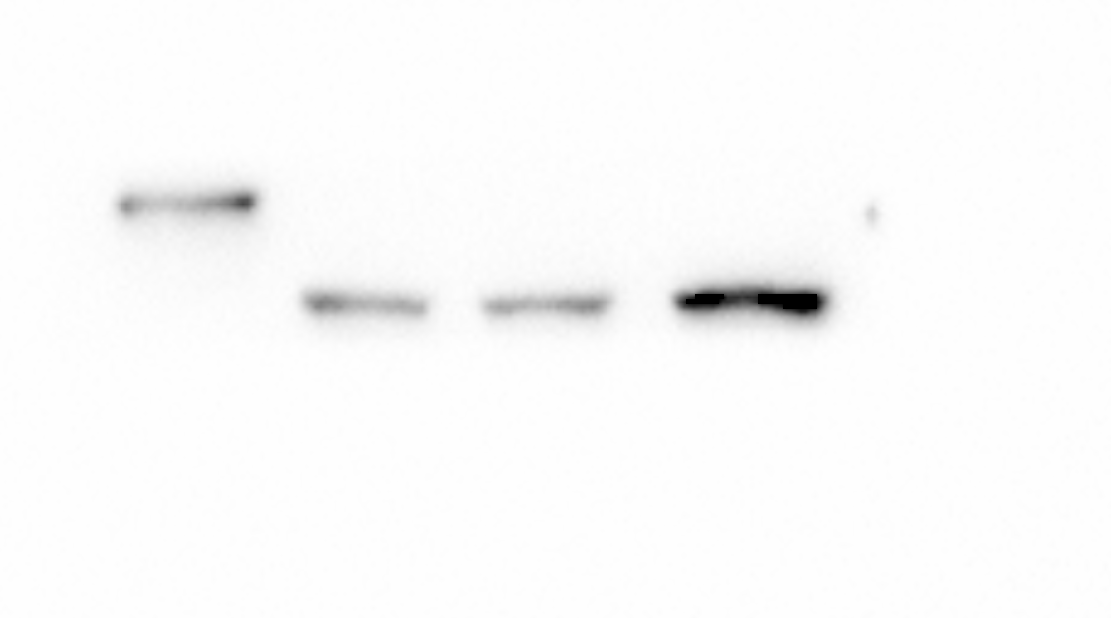

Supplement: Supplementary file 4 [file DataSheet4.zip › 2、 2h WB bands in an in vitro inflammatory model of human blood gout/JAK2-6.tif]

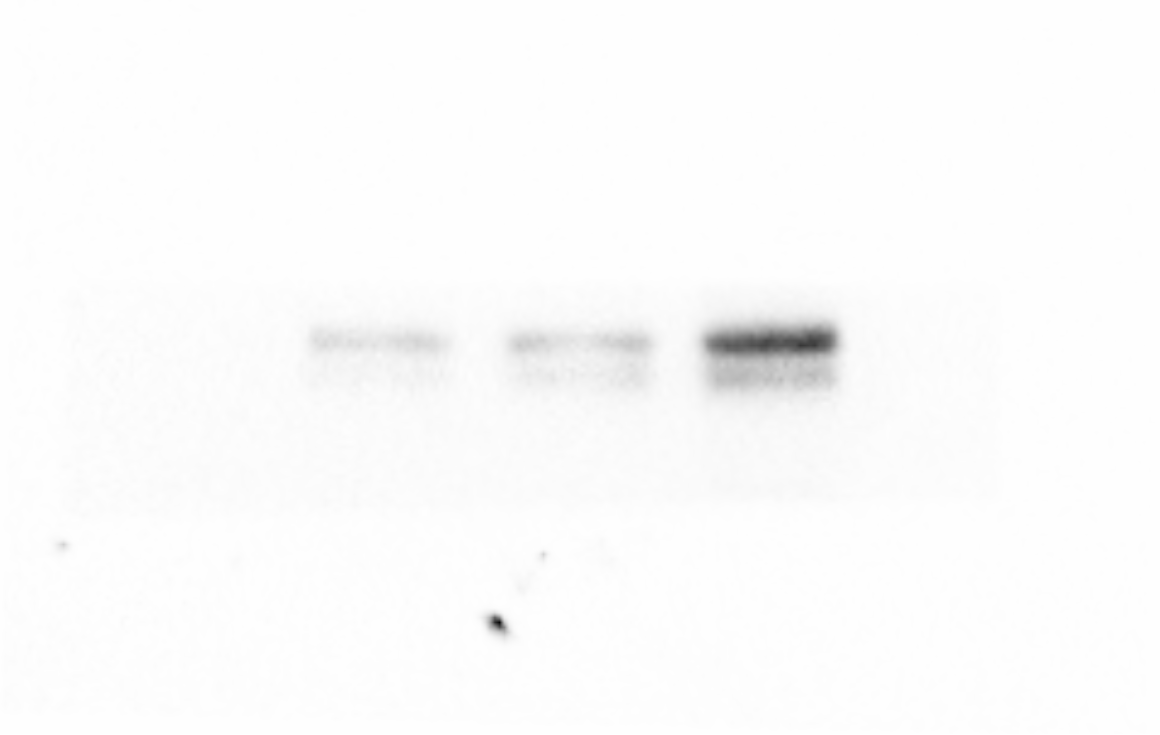

Supplement: Supplementary file 4 [file DataSheet4.zip › 2、 2h WB bands in an in vitro inflammatory model of human blood gout/STAT1-2.tif]

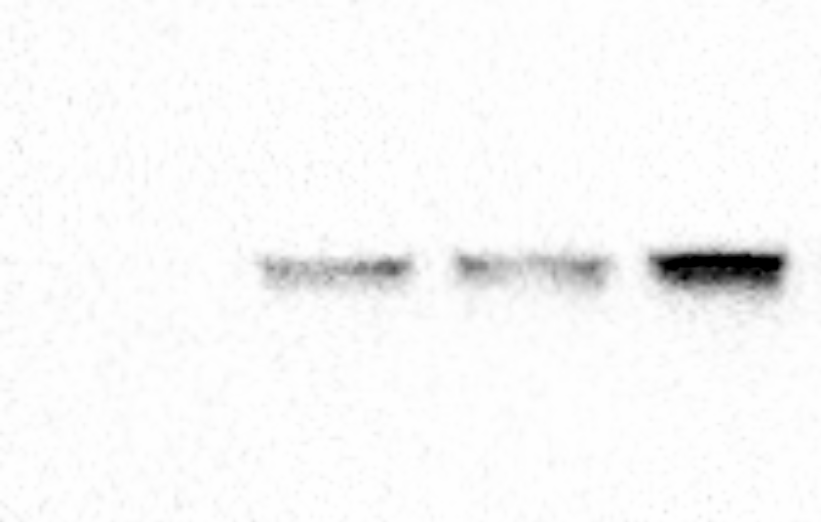

Supplement: Supplementary file 4 [file DataSheet4.zip › 2、 2h WB bands in an in vitro inflammatory model of human blood gout/STAT1-3.tif]

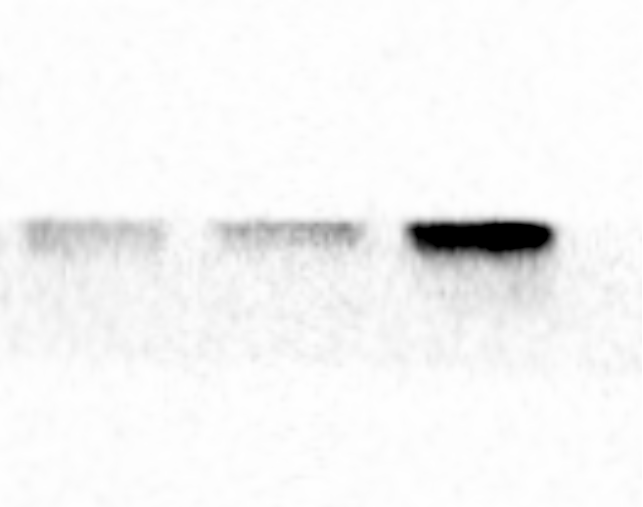

Supplement: Supplementary file 4 [file DataSheet4.zip › 2、 2h WB bands in an in vitro inflammatory model of human blood gout/STAT1-4.tif]

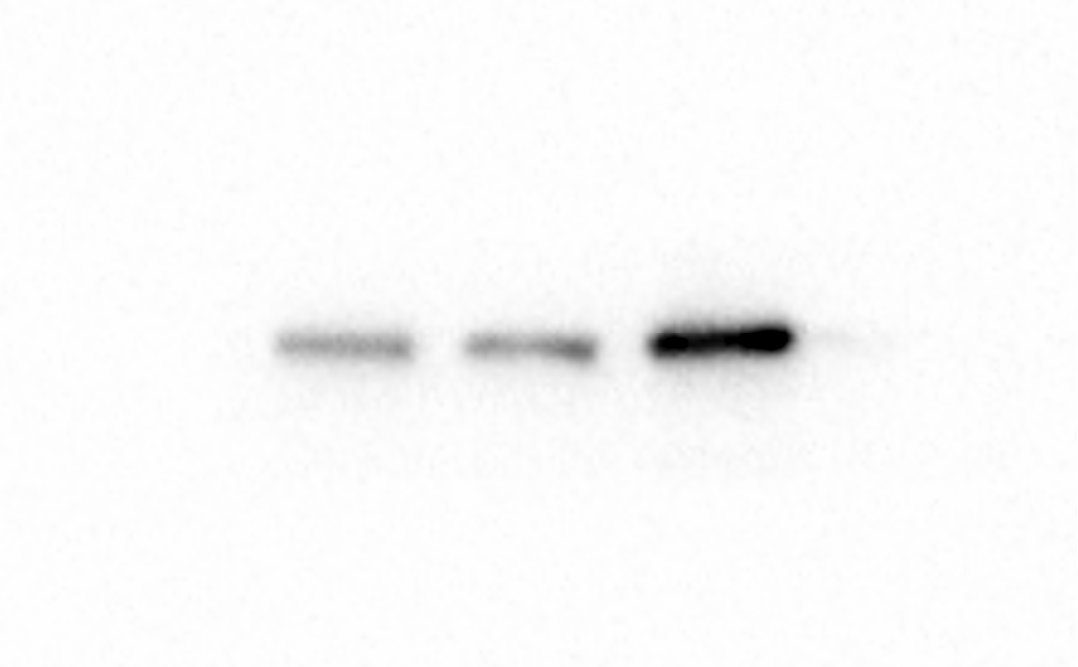

Supplement: Supplementary file 4 [file DataSheet4.zip › 2、 2h WB bands in an in vitro inflammatory model of human blood gout/STAT3-2.tif]

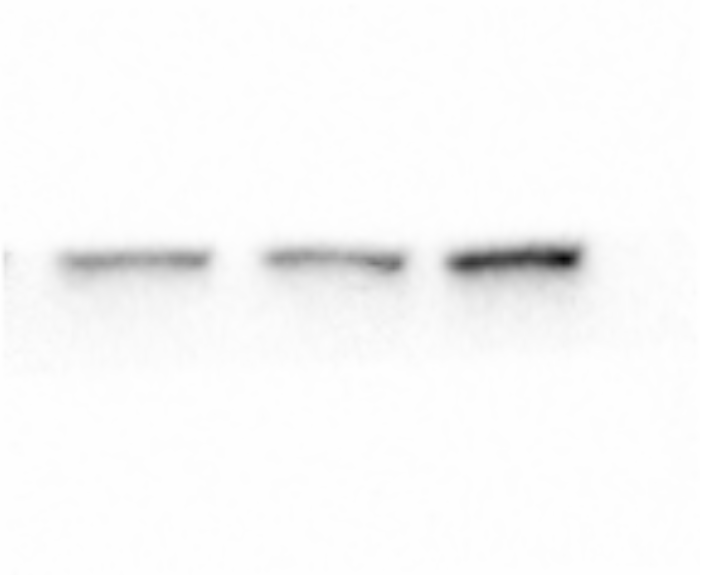

Supplement: Supplementary file 4 [file DataSheet4.zip › 2、 2h WB bands in an in vitro inflammatory model of human blood gout/STAT3-3.tif]

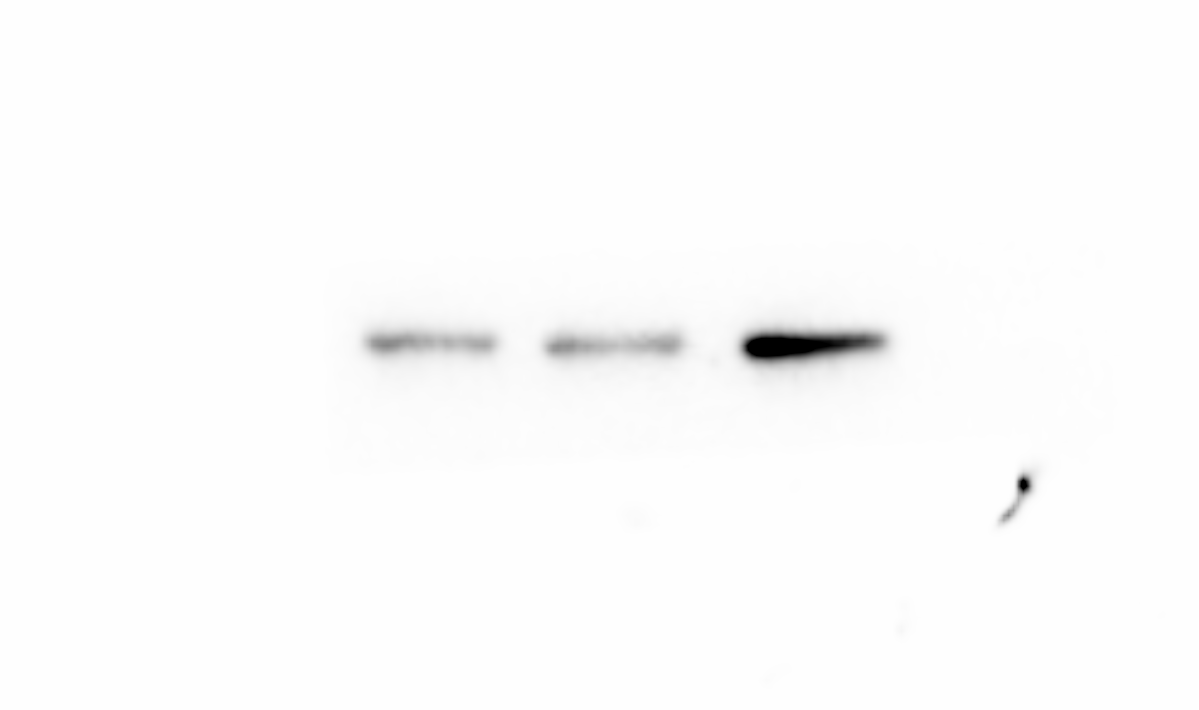

Supplement: Supplementary file 4 [file DataSheet4.zip › 2、 2h WB bands in an in vitro inflammatory model of human blood gout/STAT3-4.tif]

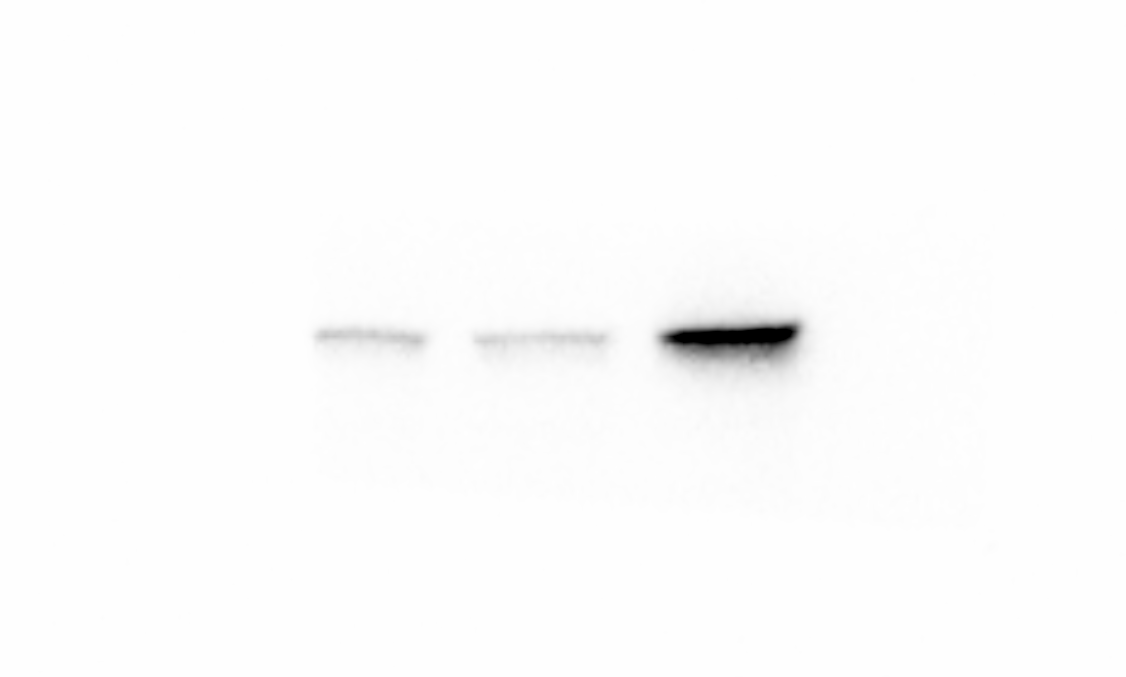

Supplement: Supplementary file 4 [file DataSheet4.zip › 2、 2h WB bands in an in vitro inflammatory model of human blood gout/p-JAK2-3.tif]

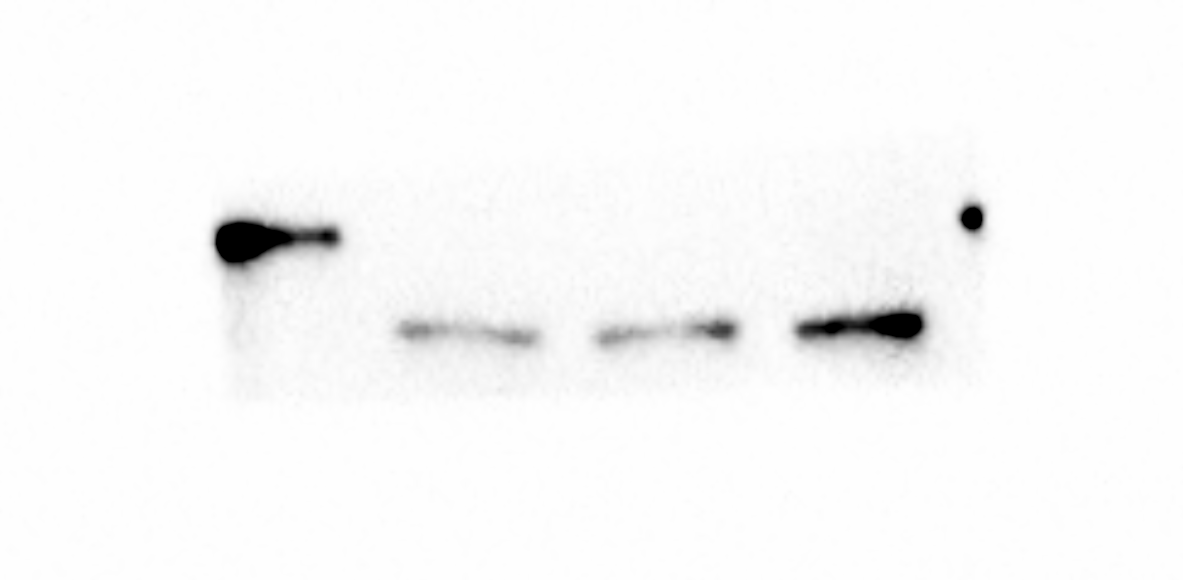

Supplement: Supplementary file 4 [file DataSheet4.zip › 2、 2h WB bands in an in vitro inflammatory model of human blood gout/p-JAK2-4.tif]

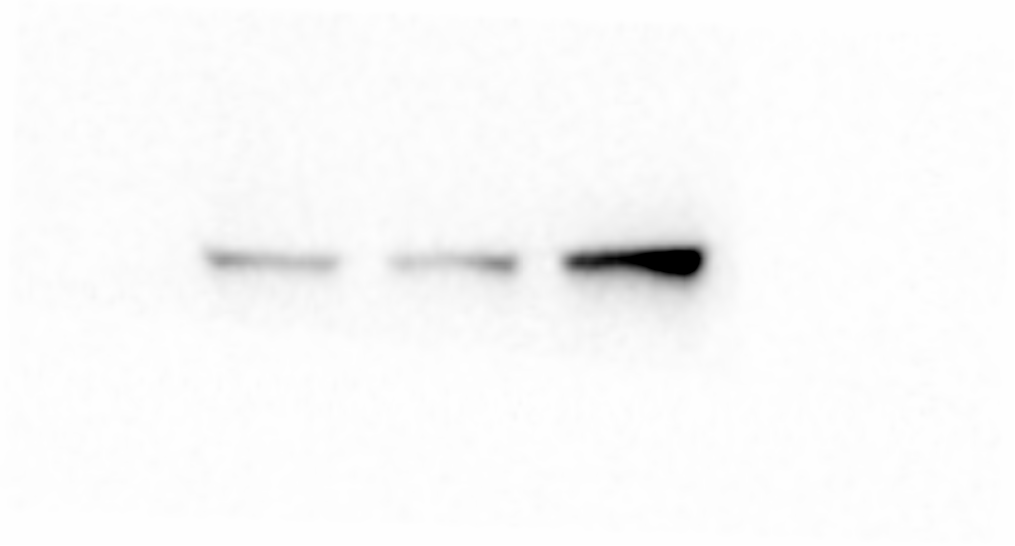

Supplement: Supplementary file 4 [file DataSheet4.zip › 2、 2h WB bands in an in vitro inflammatory model of human blood gout/p-STAT1-3.tif]

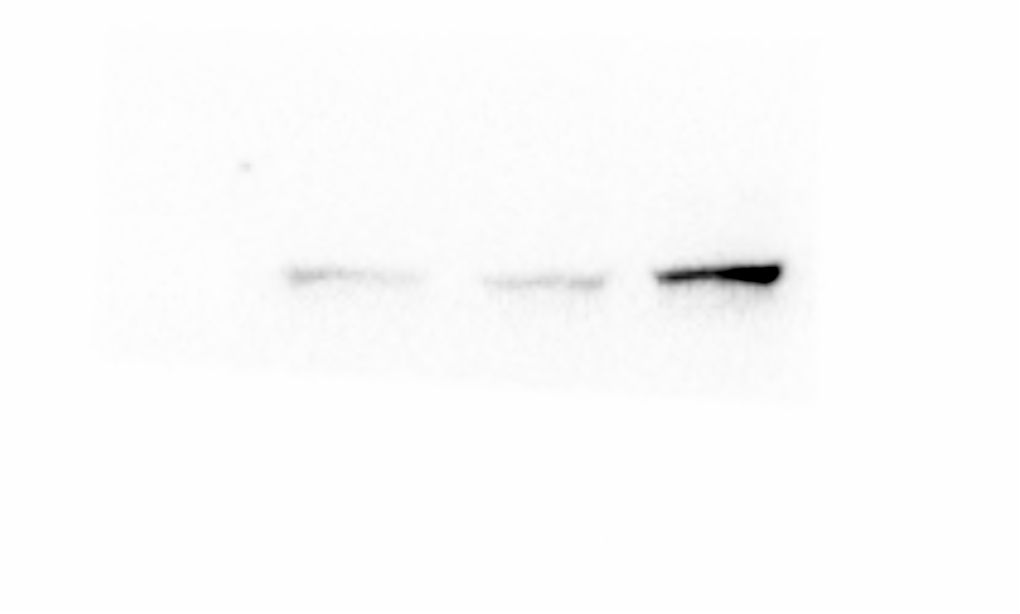

Supplement: Supplementary file 4 [file DataSheet4.zip › 2、 2h WB bands in an in vitro inflammatory model of human blood gout/p-STAT1-4.tif]

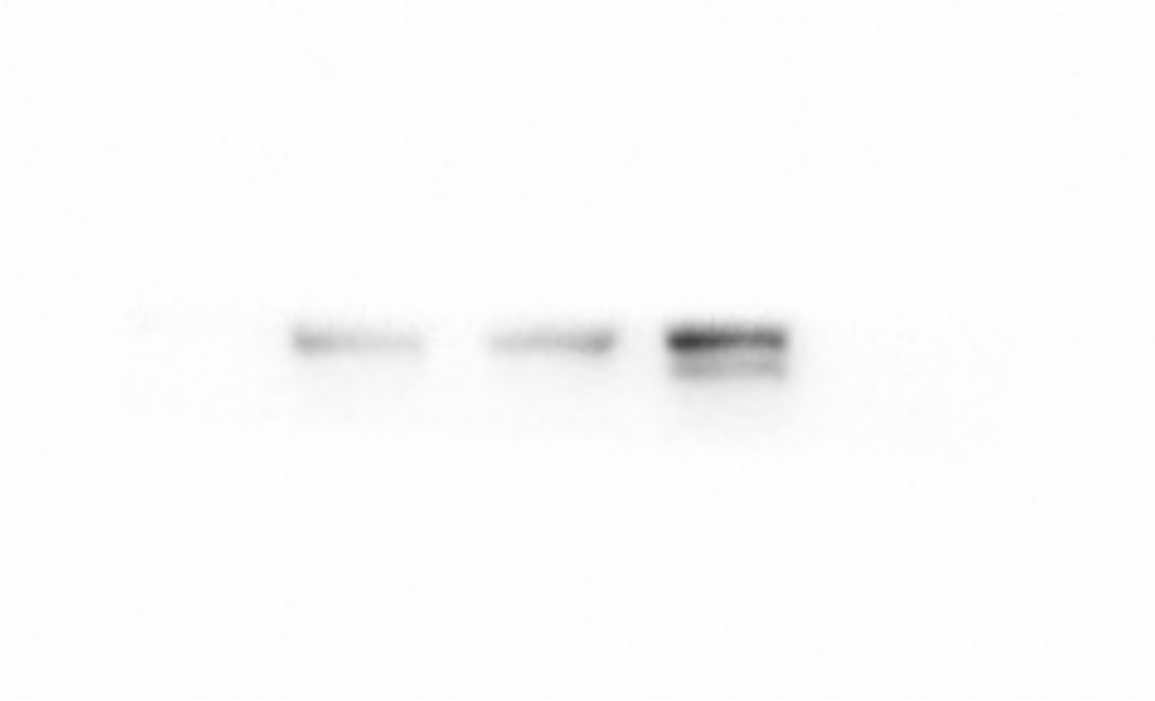

Supplement: Supplementary file 4 [file DataSheet4.zip › 2、 2h WB bands in an in vitro inflammatory model of human blood gout/p-STAT3-2.tif]

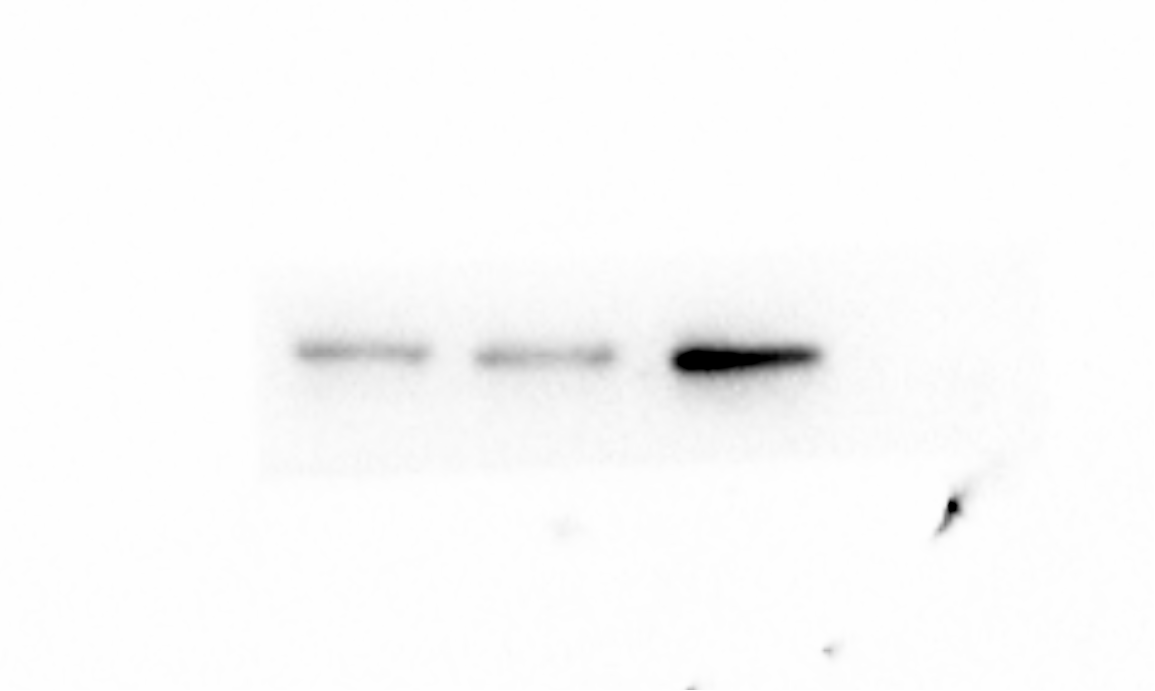

Supplement: Supplementary file 4 [file DataSheet4.zip › 2、 2h WB bands in an in vitro inflammatory model of human blood gout/p-STAT3-3.tif]

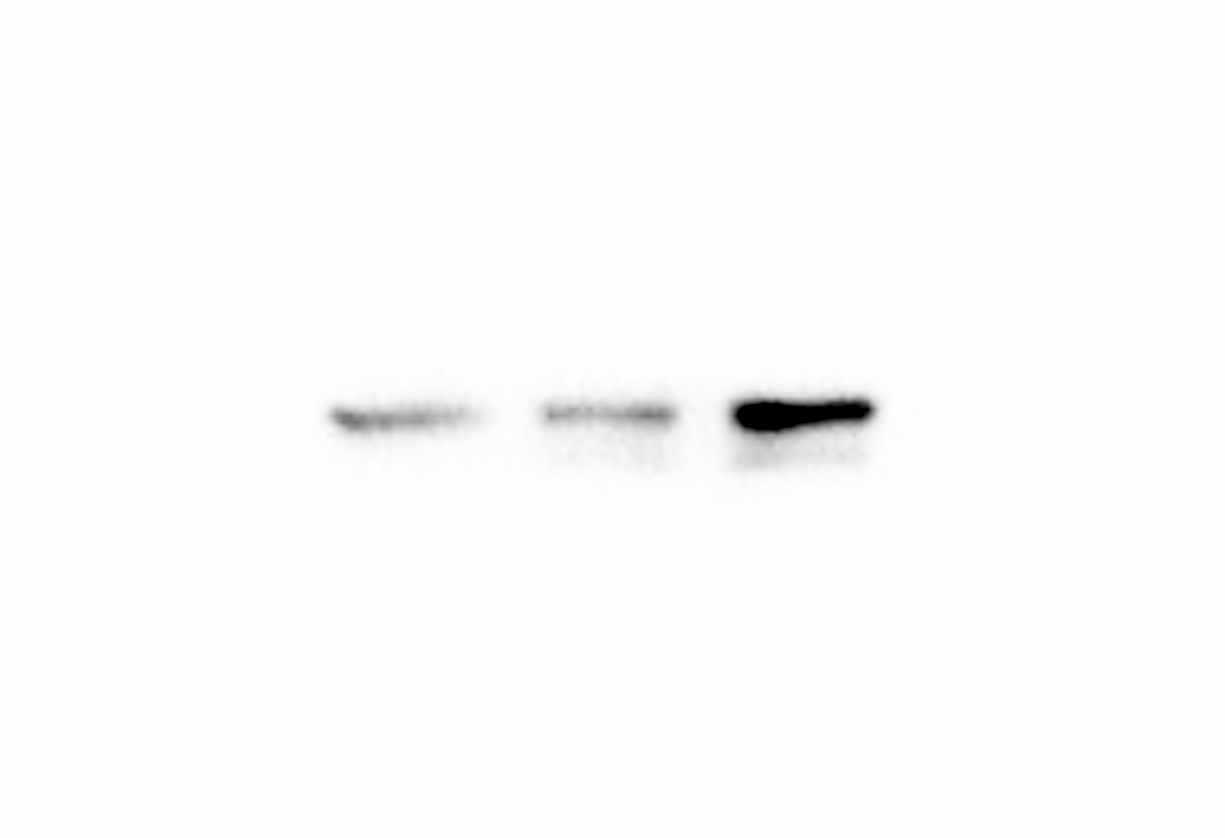

Supplement: Supplementary file 4 [file DataSheet4.zip › 2、 2h WB bands in an in vitro inflammatory model of human blood gout/p-STAT3-4.tif]

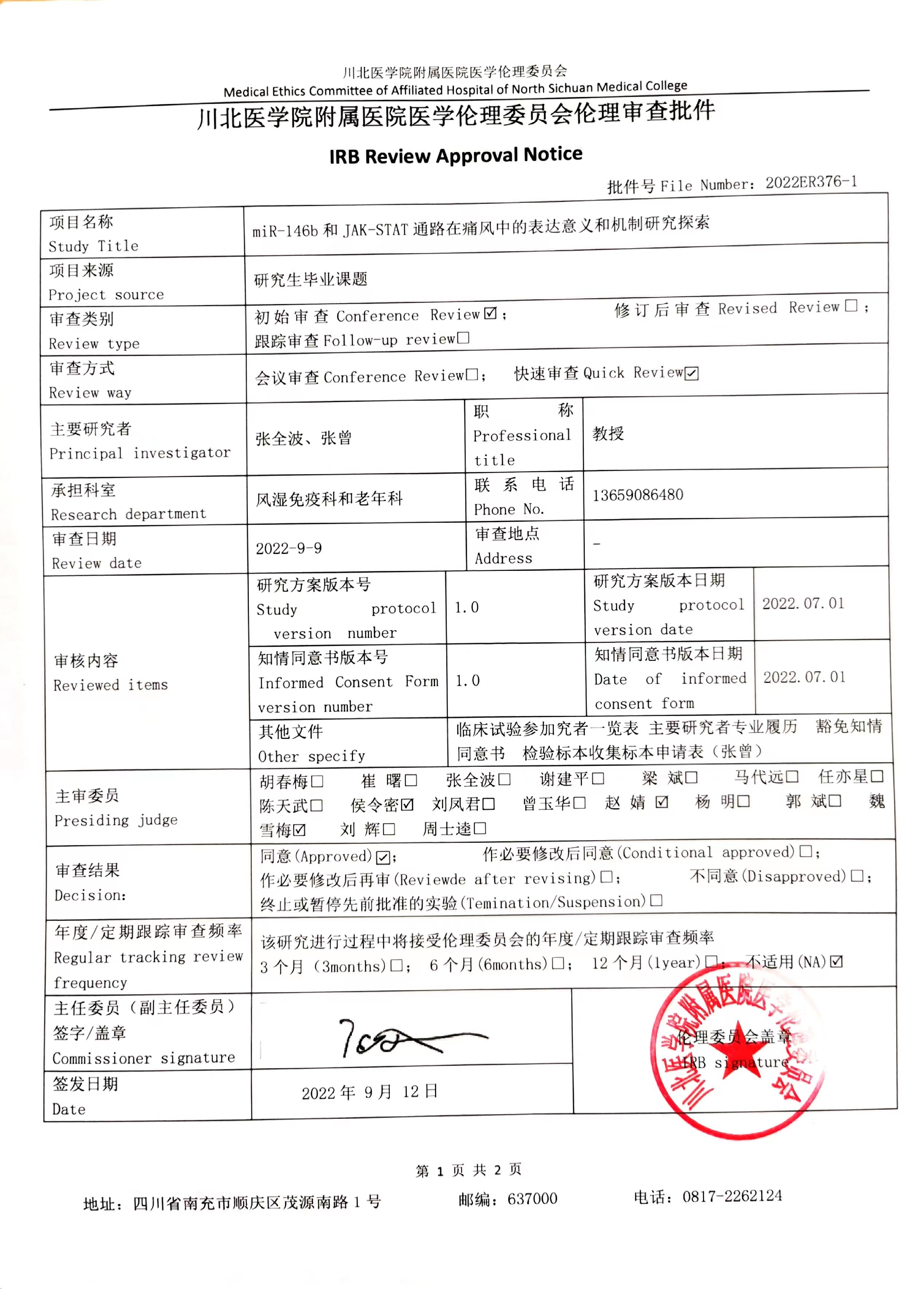

Supplement: Supplementary file 5 [file Image1.jpeg]

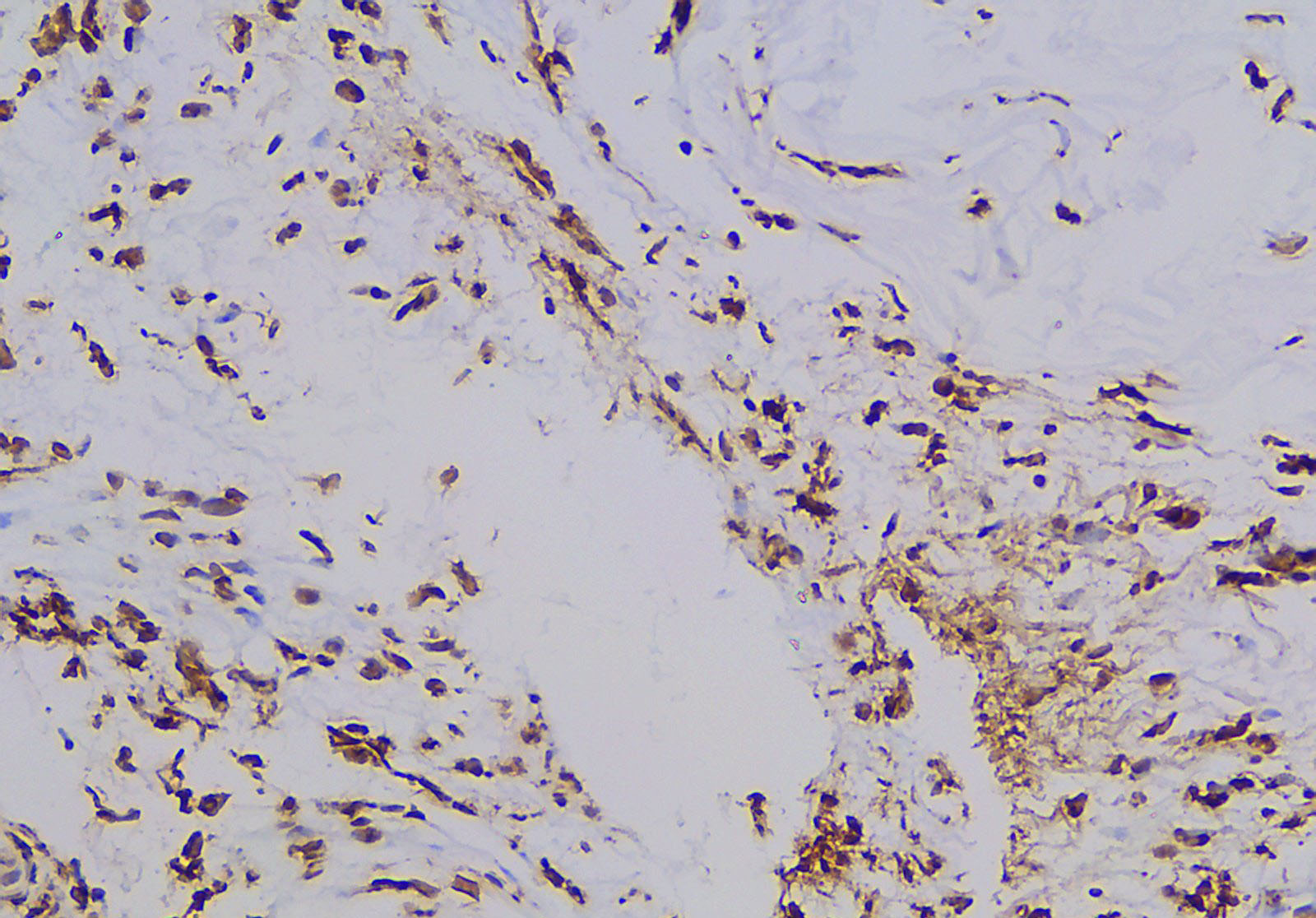

Supplement: Supplementary file 6 [file DataSheet1.zip › IHC/IL6 KO MSU12h pJAK2.jpg]

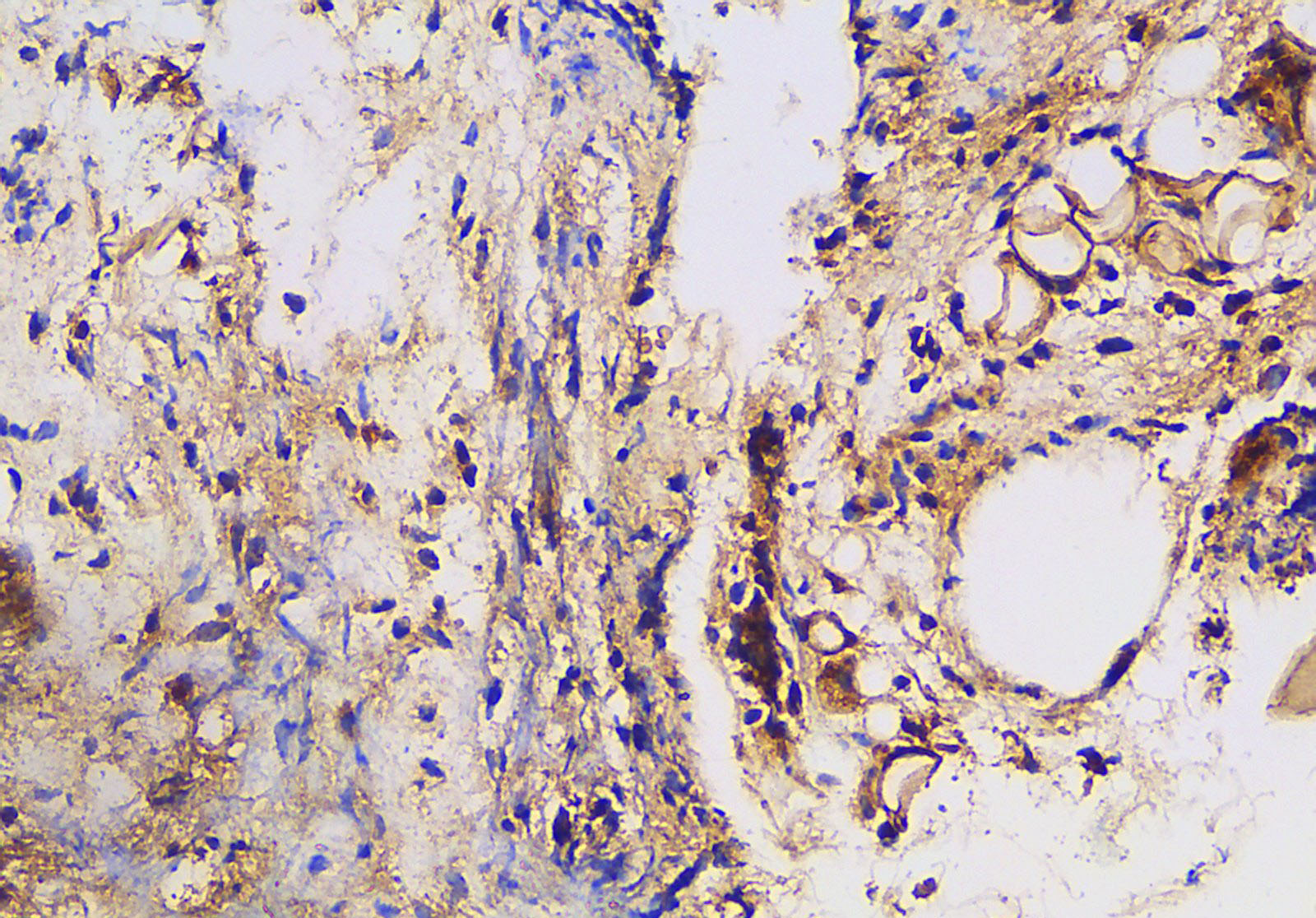

Supplement: Supplementary file 6 [file DataSheet1.zip › IHC/IL6 KO MSU12h pSTAT1.jpg]

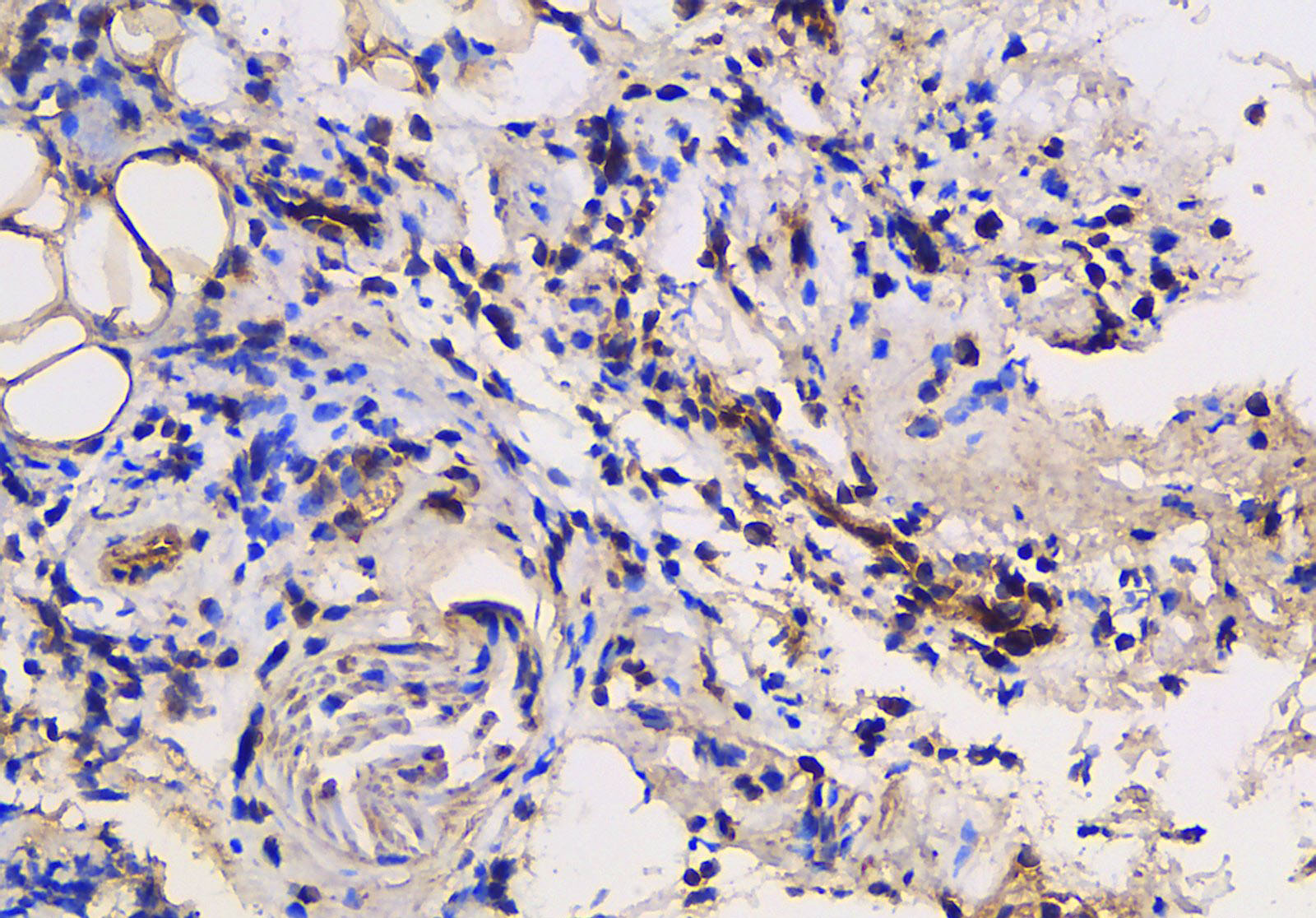

Supplement: Supplementary file 6 [file DataSheet1.zip › IHC/IL6 KO MSU12h pSTAT3.jpg]

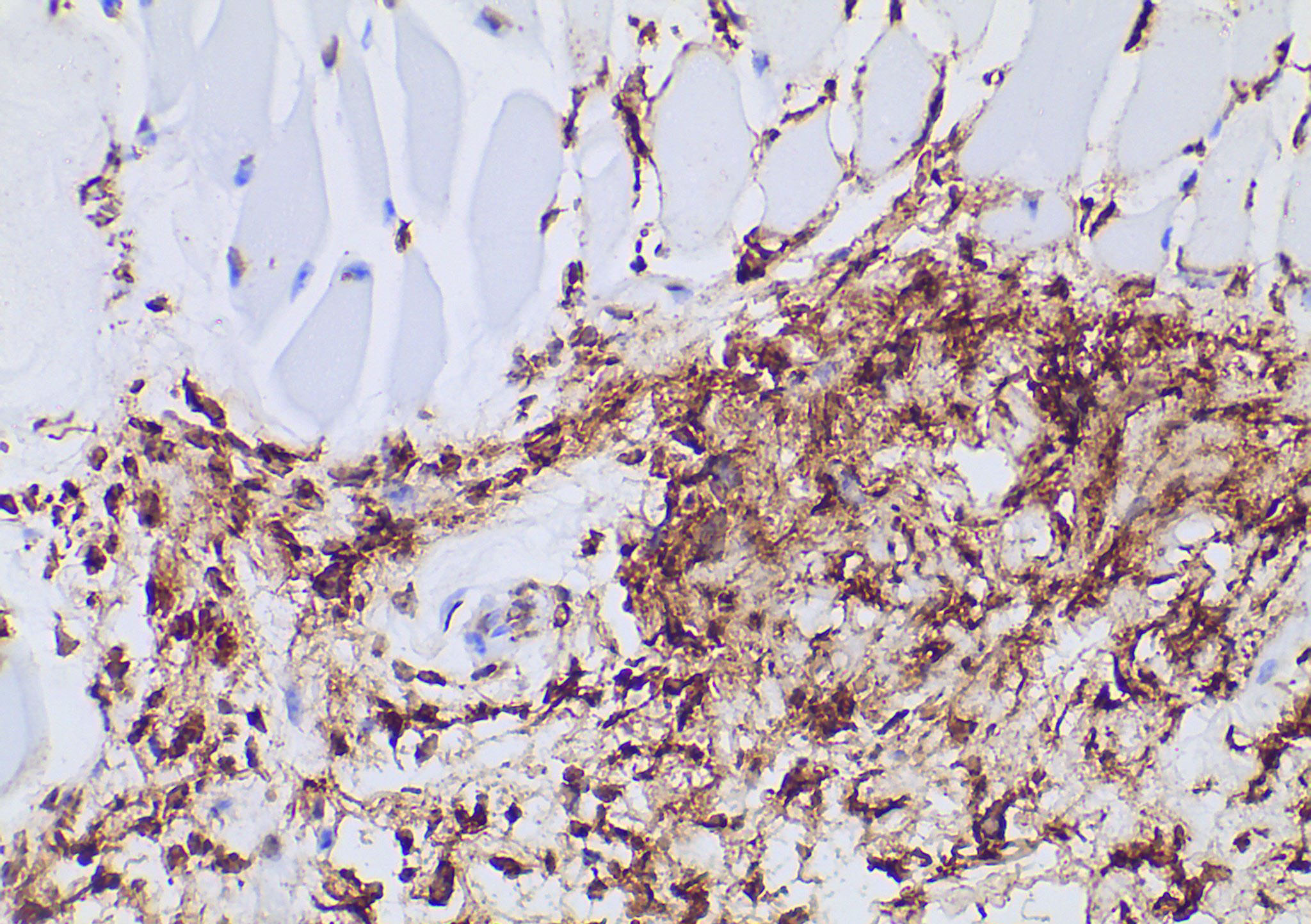

Supplement: Supplementary file 6 [file DataSheet1.zip › IHC/WT MSU12h pJAK2.jpg]

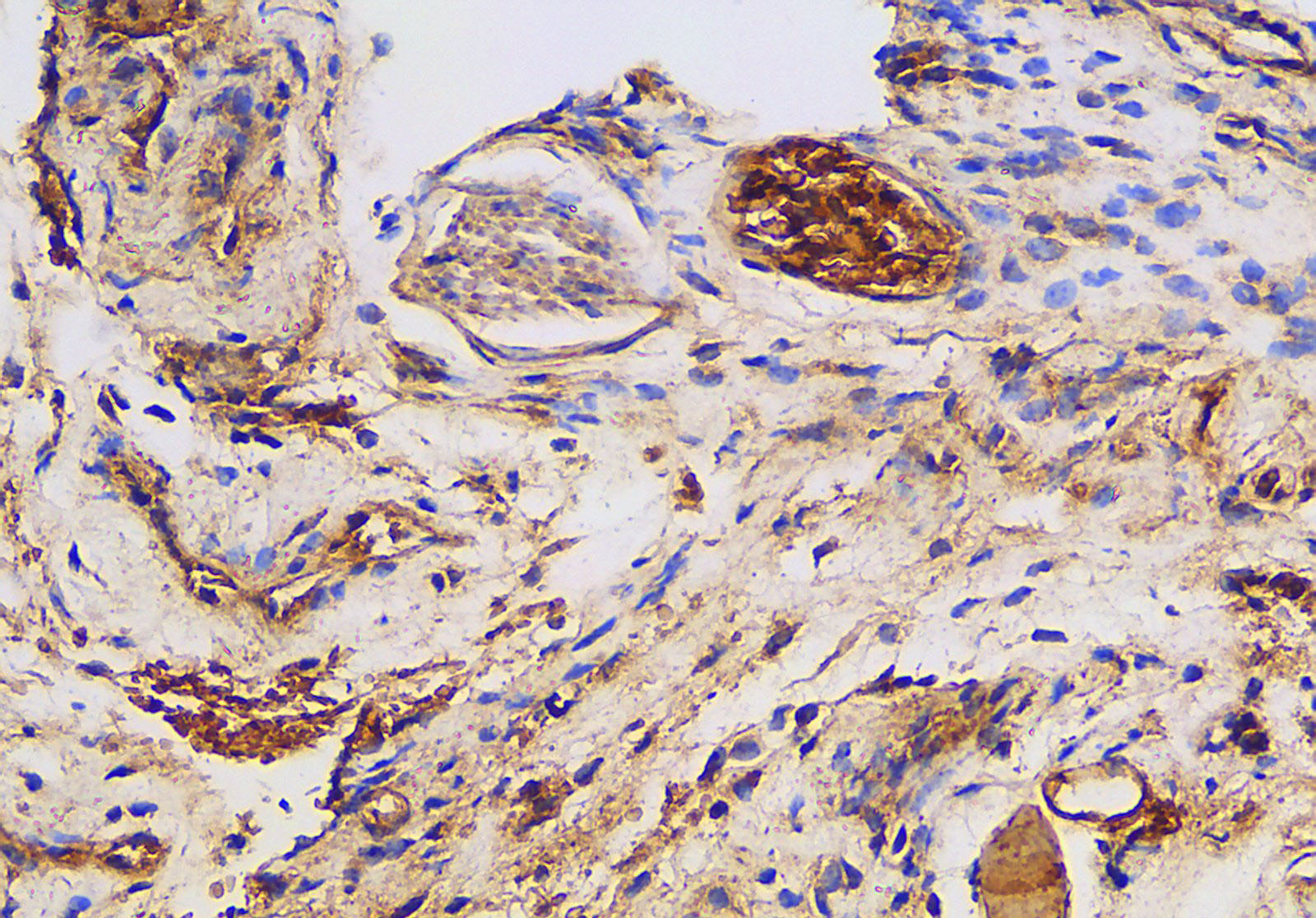

Supplement: Supplementary file 6 [file DataSheet1.zip › IHC/WT MSU12h pSTAT1.jpg]

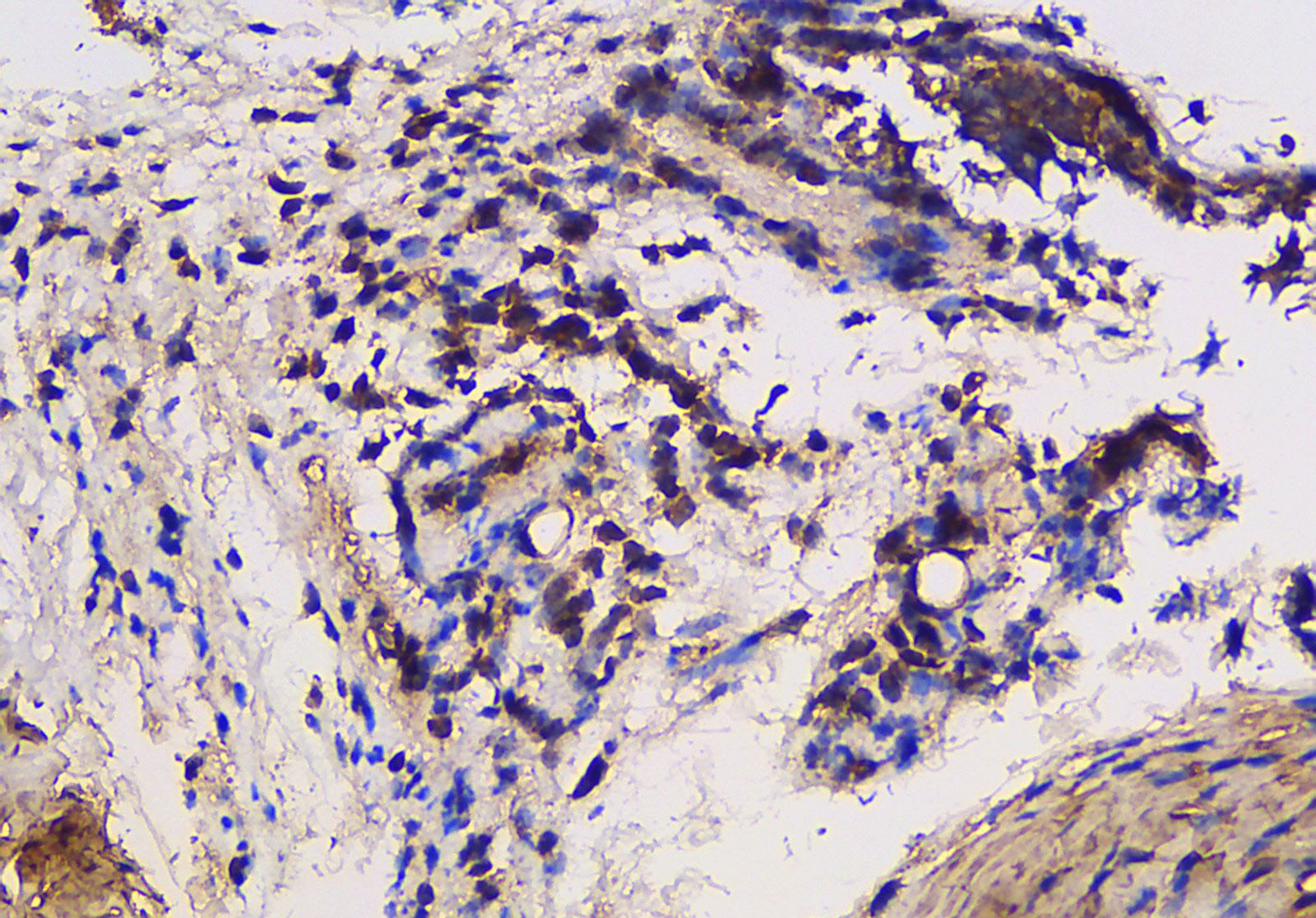

Supplement: Supplementary file 6 [file DataSheet1.zip › IHC/WT MSU12h pSTAT3.jpg]

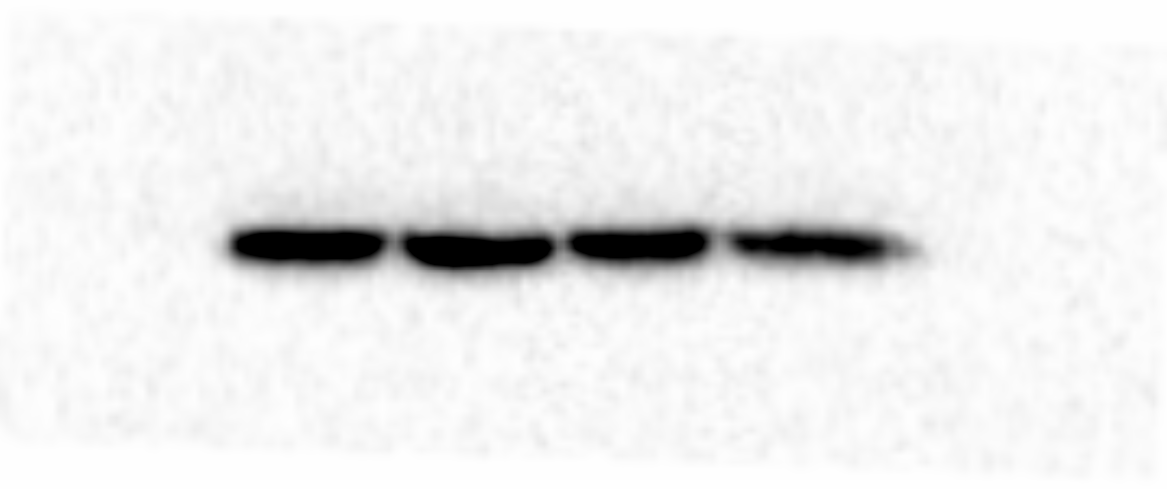

Supplement: Supplementary file 7 [file DataSheet6.zip › 4、IL-6 knockout mouse and WT mouse WB strips/GAPDH-2.tif]

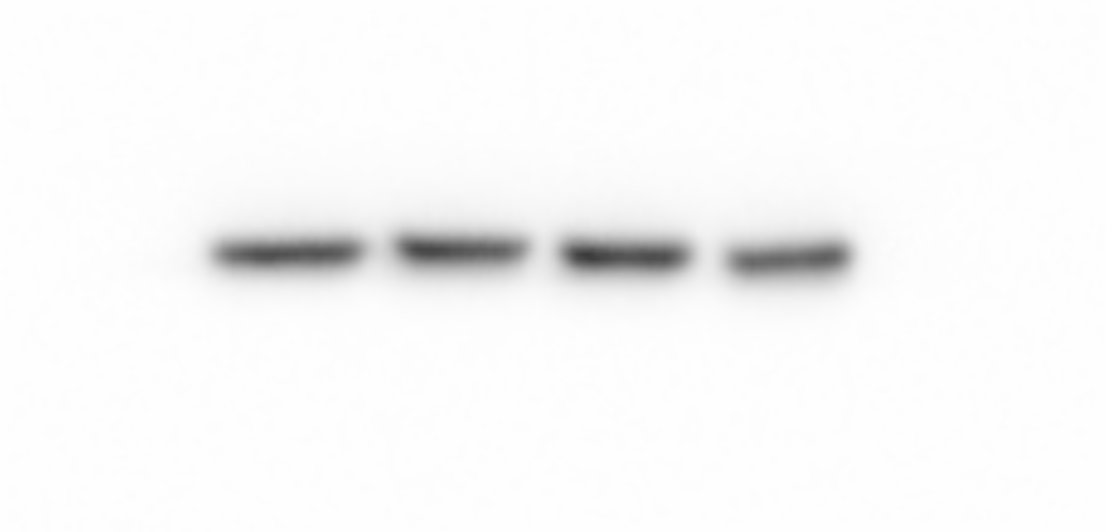

Supplement: Supplementary file 7 [file DataSheet6.zip › 4、IL-6 knockout mouse and WT mouse WB strips/GAPDH-4.tif]

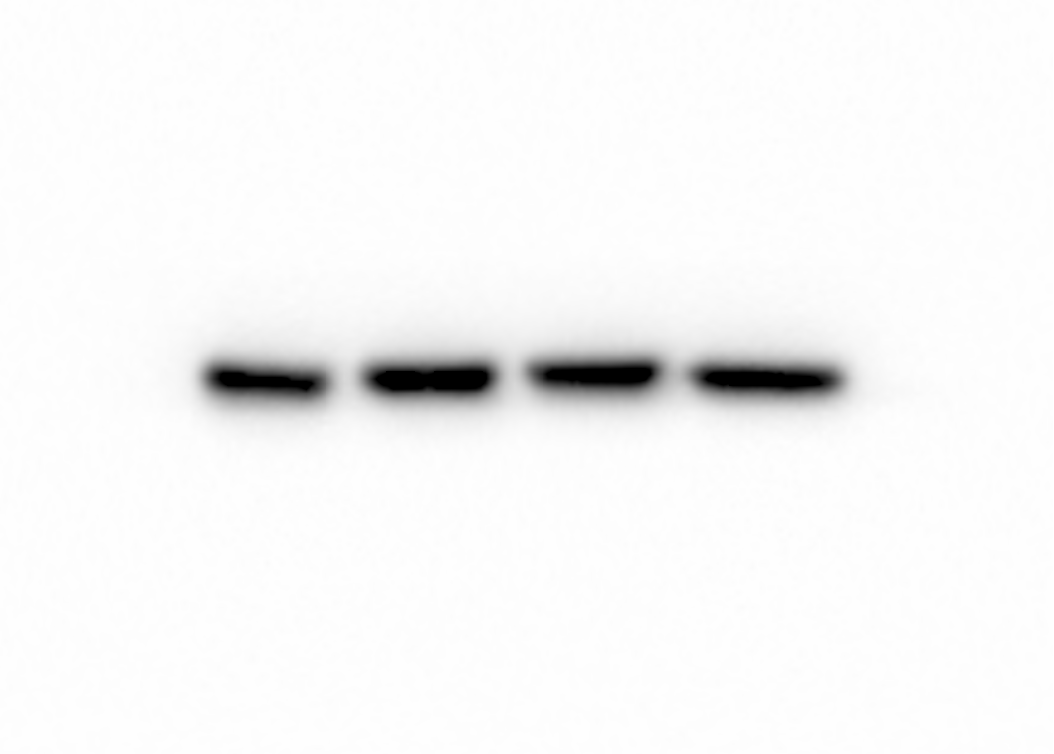

Supplement: Supplementary file 7 [file DataSheet6.zip › 4、IL-6 knockout mouse and WT mouse WB strips/GAPDH-5.tif]

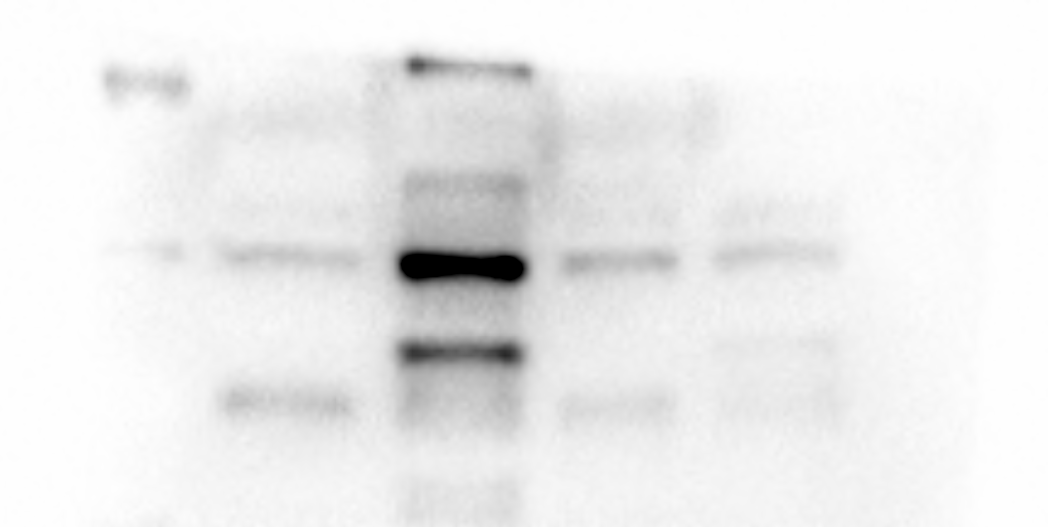

Supplement: Supplementary file 7 [file DataSheet6.zip › 4、IL-6 knockout mouse and WT mouse WB strips/IL-1β-2.tif]

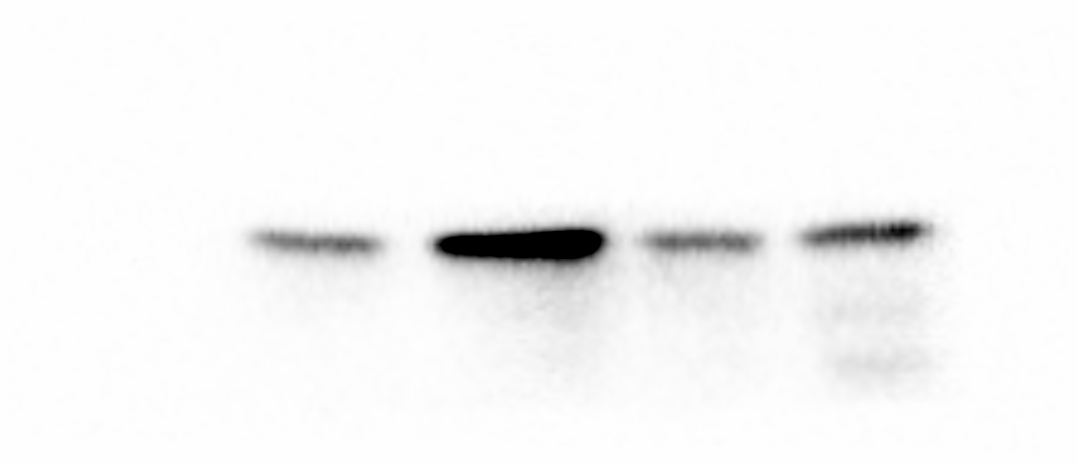

Supplement: Supplementary file 7 [file DataSheet6.zip › 4、IL-6 knockout mouse and WT mouse WB strips/IL-1β-3.tif]

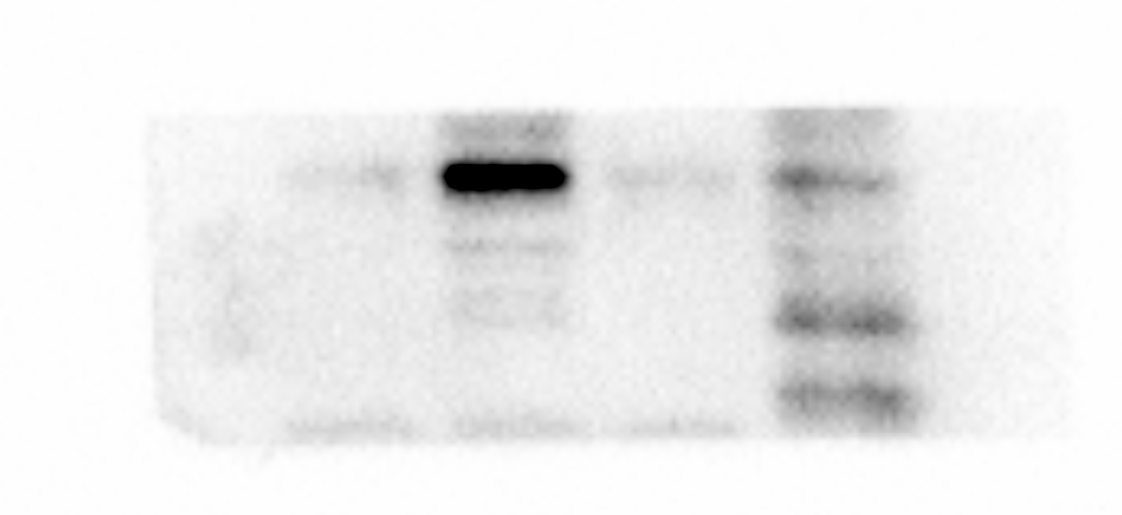

Supplement: Supplementary file 7 [file DataSheet6.zip › 4、IL-6 knockout mouse and WT mouse WB strips/IL-1β-4.tif]

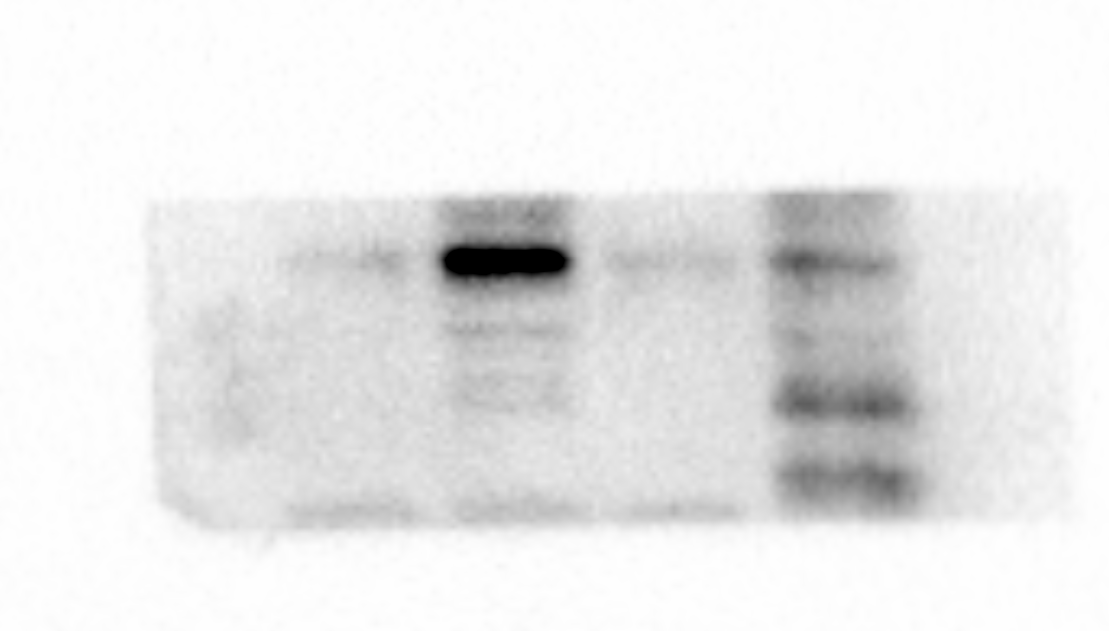

Supplement: Supplementary file 7 [file DataSheet6.zip › 4、IL-6 knockout mouse and WT mouse WB strips/IL-1β-5.tif]

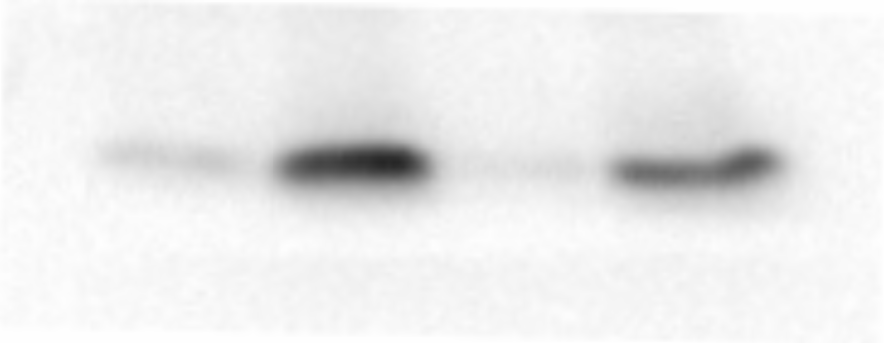

Supplement: Supplementary file 7 [file DataSheet6.zip › 4、IL-6 knockout mouse and WT mouse WB strips/IL6-2.tif]

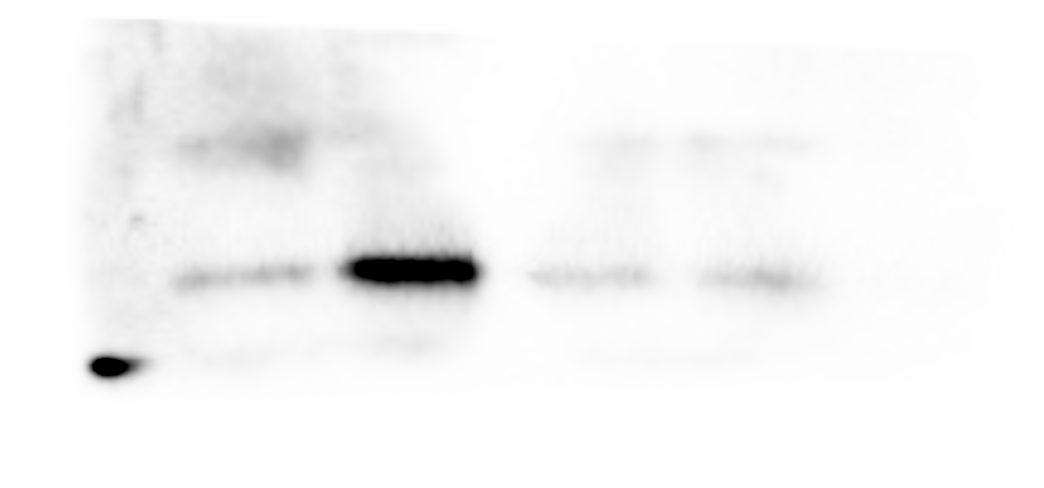

Supplement: Supplementary file 7 [file DataSheet6.zip › 4、IL-6 knockout mouse and WT mouse WB strips/IL6-3.tif]

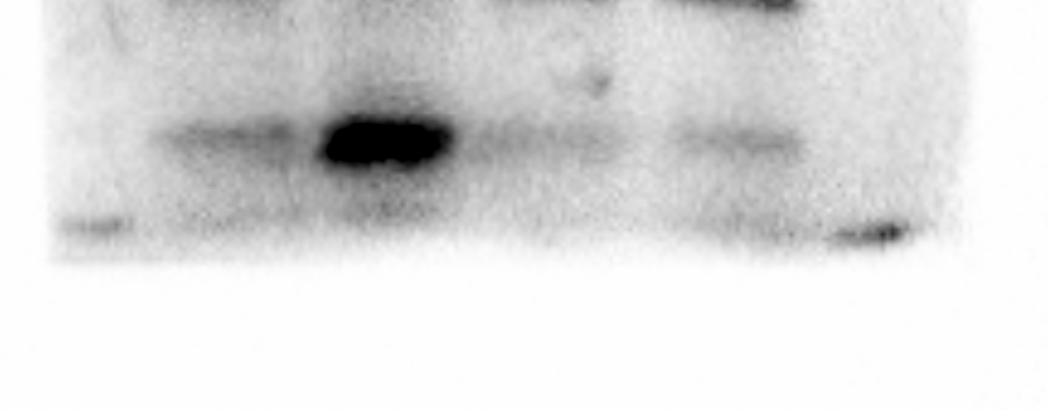

Supplement: Supplementary file 7 [file DataSheet6.zip › 4、IL-6 knockout mouse and WT mouse WB strips/IL6-4.tif]

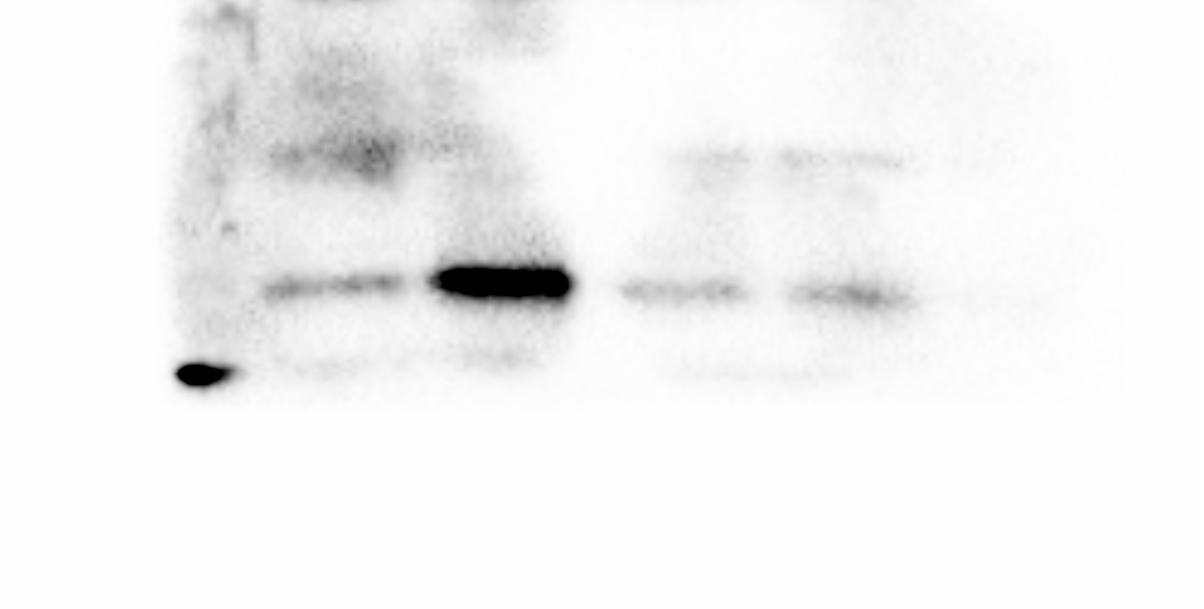

Supplement: Supplementary file 7 [file DataSheet6.zip › 4、IL-6 knockout mouse and WT mouse WB strips/IL6-5.tif]

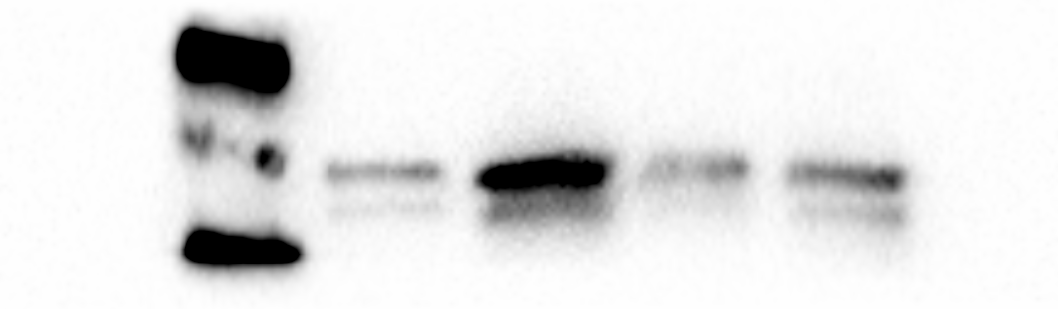

Supplement: Supplementary file 7 [file DataSheet6.zip › 4、IL-6 knockout mouse and WT mouse WB strips/JAK2-2.tif]

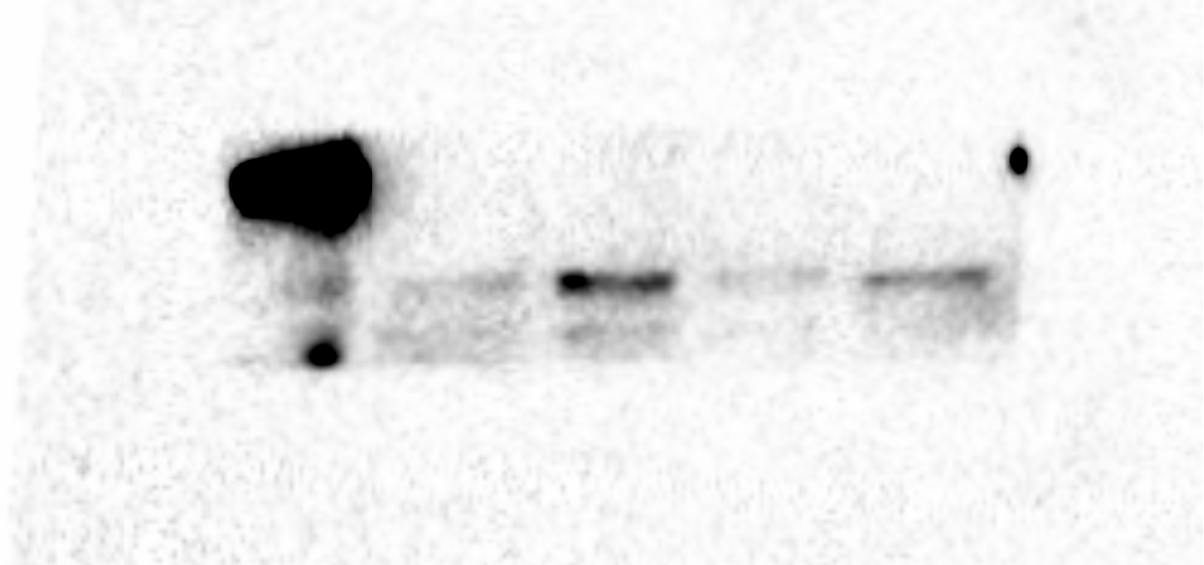

Supplement: Supplementary file 7 [file DataSheet6.zip › 4、IL-6 knockout mouse and WT mouse WB strips/JAK2-3.tif]

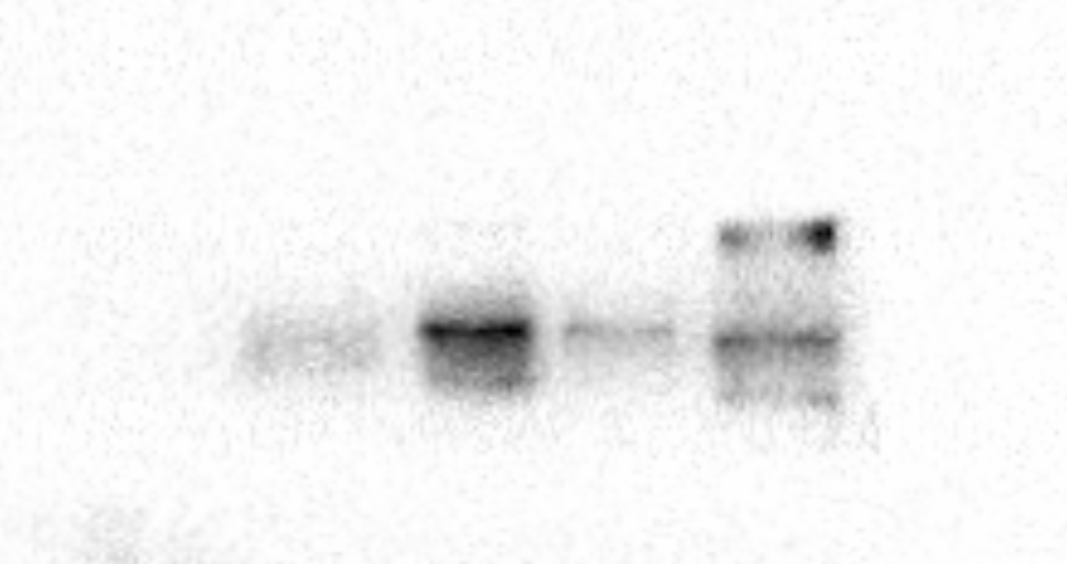

Supplement: Supplementary file 7 [file DataSheet6.zip › 4、IL-6 knockout mouse and WT mouse WB strips/JAK2-4.tif]

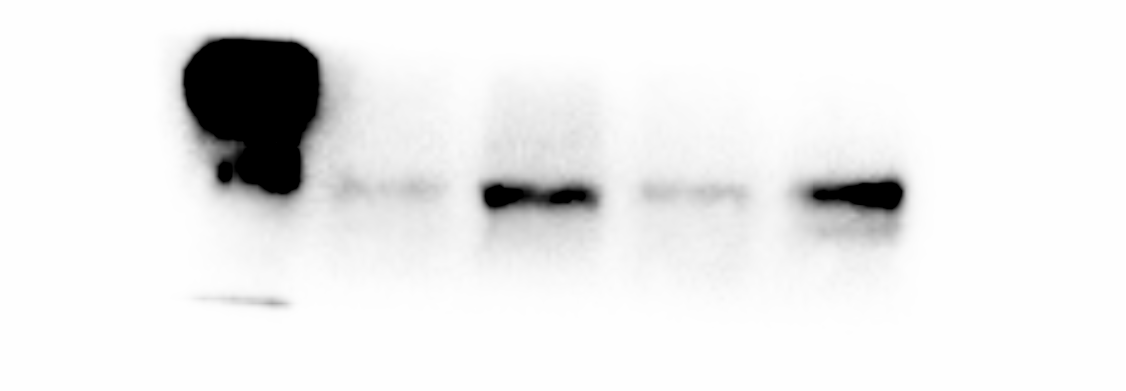

Supplement: Supplementary file 7 [file DataSheet6.zip › 4、IL-6 knockout mouse and WT mouse WB strips/P-JAK2-2.tif]

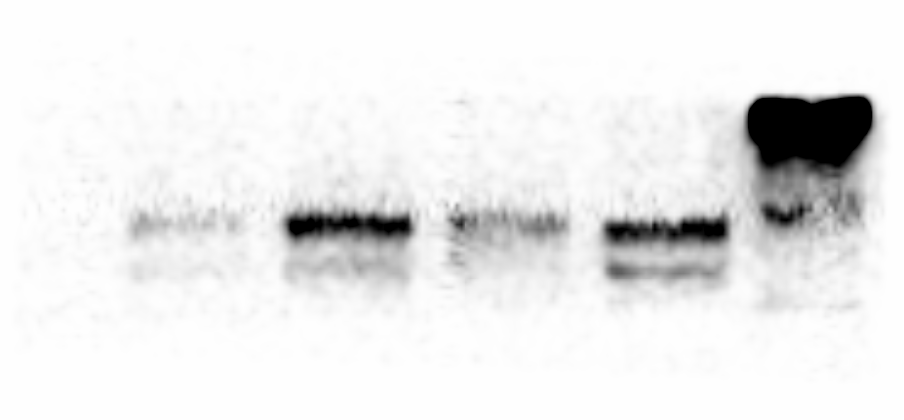

Supplement: Supplementary file 7 [file DataSheet6.zip › 4、IL-6 knockout mouse and WT mouse WB strips/P-JAK2-3.tif]

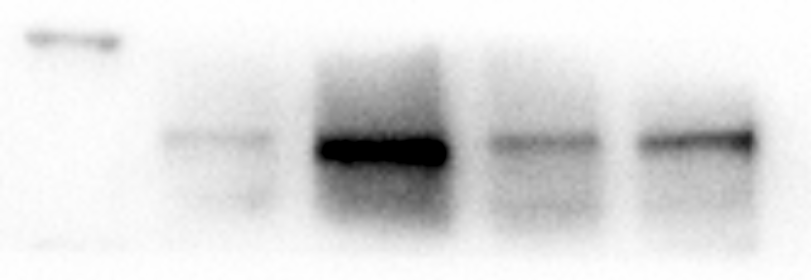

Supplement: Supplementary file 7 [file DataSheet6.zip › 4、IL-6 knockout mouse and WT mouse WB strips/P-JAK2-4.tif]

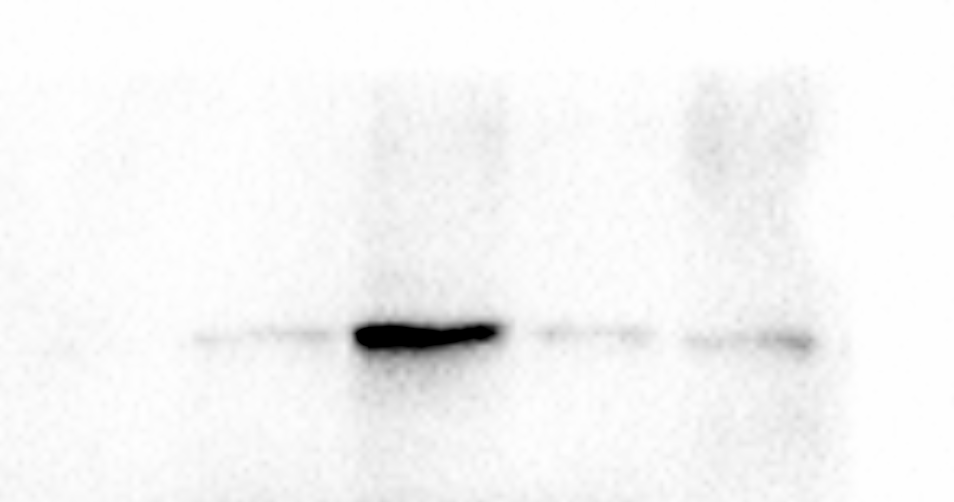

Supplement: Supplementary file 7 [file DataSheet6.zip › 4、IL-6 knockout mouse and WT mouse WB strips/P-JAK2-5.tif]

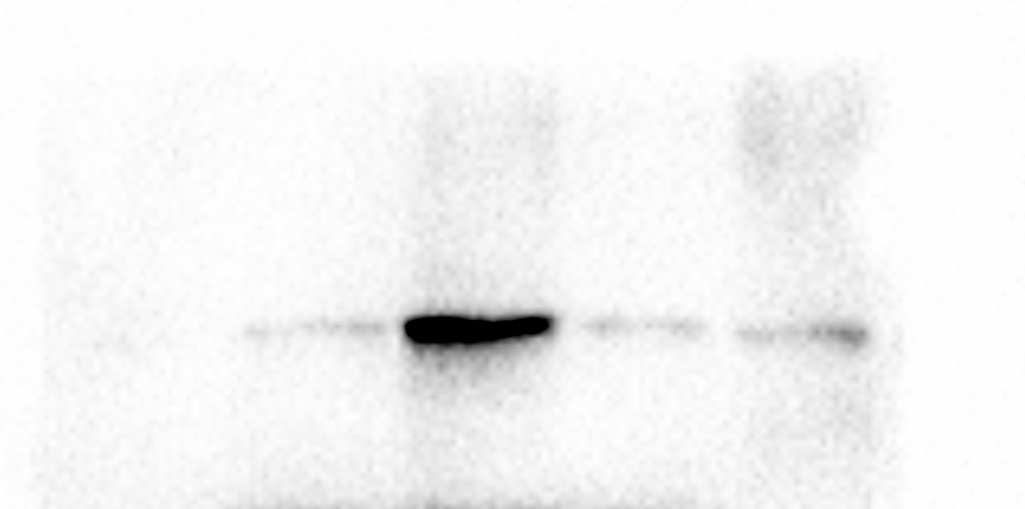

Supplement: Supplementary file 7 [file DataSheet6.zip › 4、IL-6 knockout mouse and WT mouse WB strips/P-JAK2-6.tif]

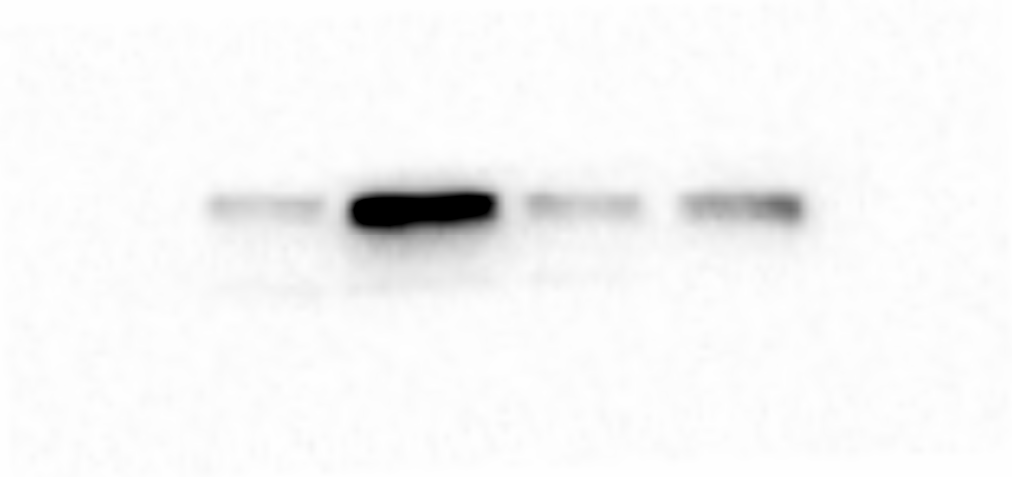

Supplement: Supplementary file 7 [file DataSheet6.zip › 4、IL-6 knockout mouse and WT mouse WB strips/STAT1-2.tif]

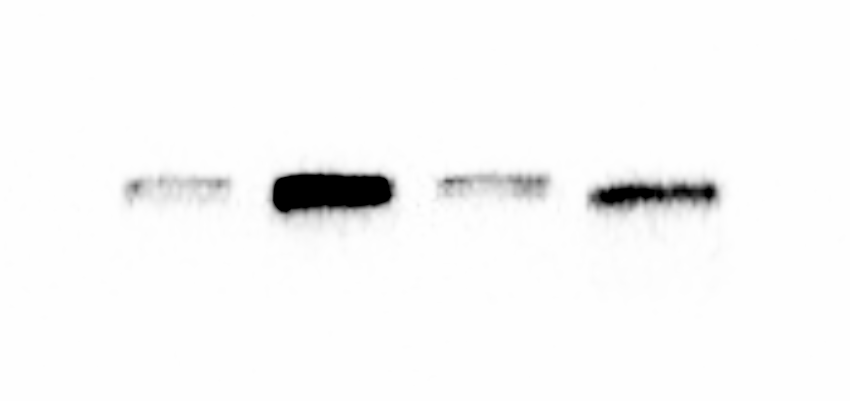

Supplement: Supplementary file 7 [file DataSheet6.zip › 4、IL-6 knockout mouse and WT mouse WB strips/STAT1-3.tif]

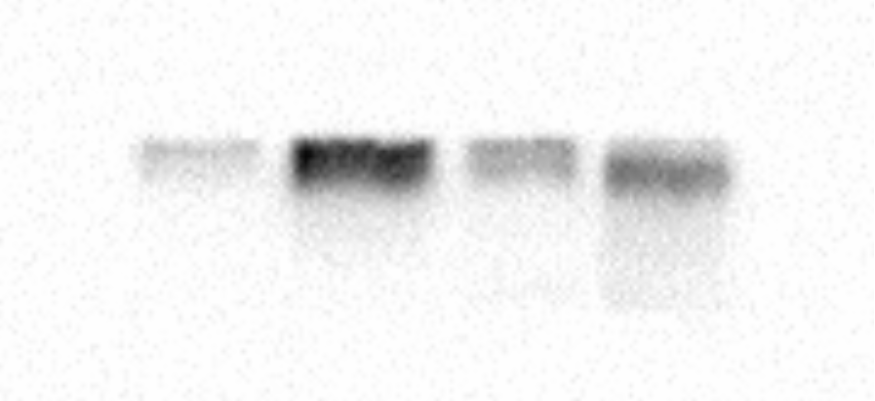

Supplement: Supplementary file 7 [file DataSheet6.zip › 4、IL-6 knockout mouse and WT mouse WB strips/STAT1-4.tif]

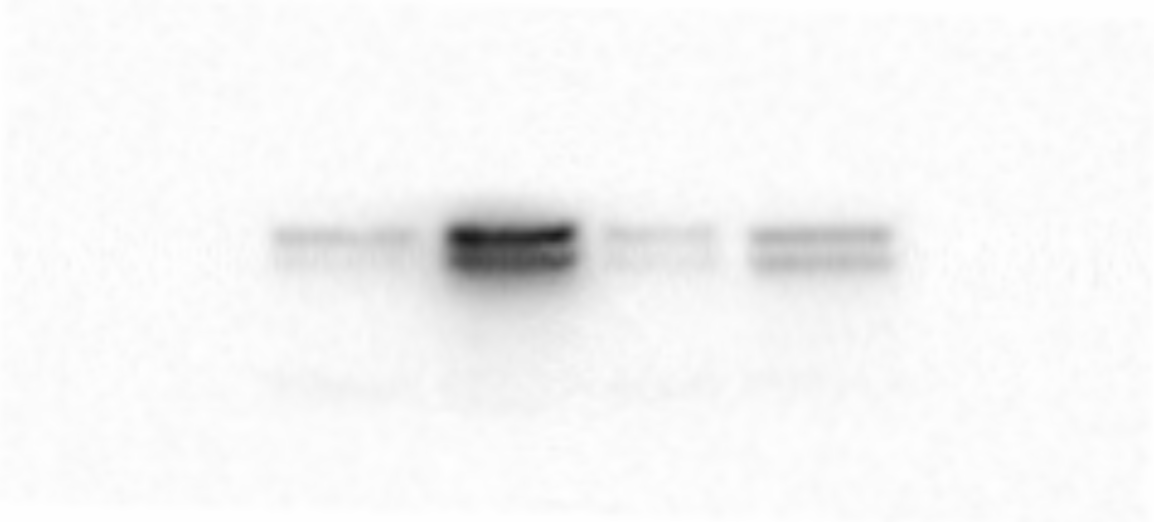

Supplement: Supplementary file 7 [file DataSheet6.zip › 4、IL-6 knockout mouse and WT mouse WB strips/STAT1-5.tif]

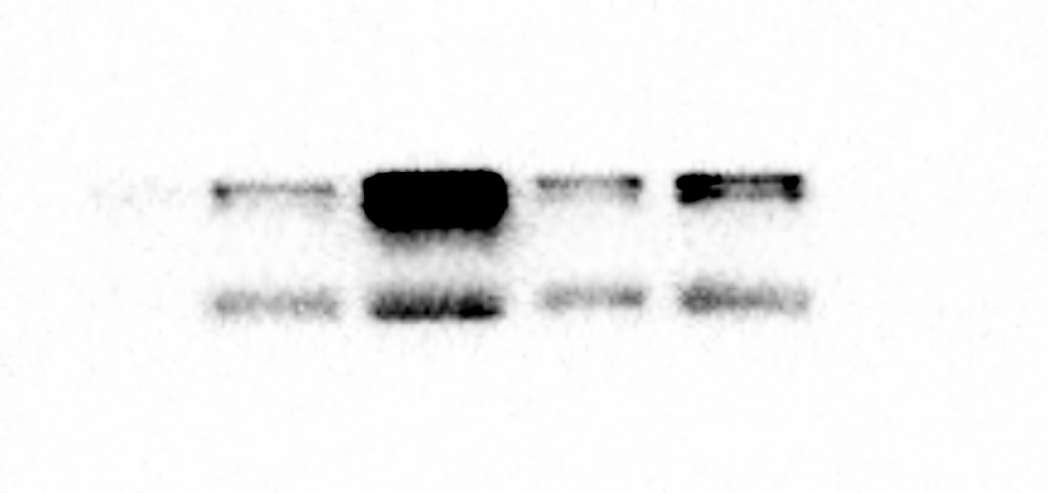

Supplement: Supplementary file 7 [file DataSheet6.zip › 4、IL-6 knockout mouse and WT mouse WB strips/STAT1-6.tif]

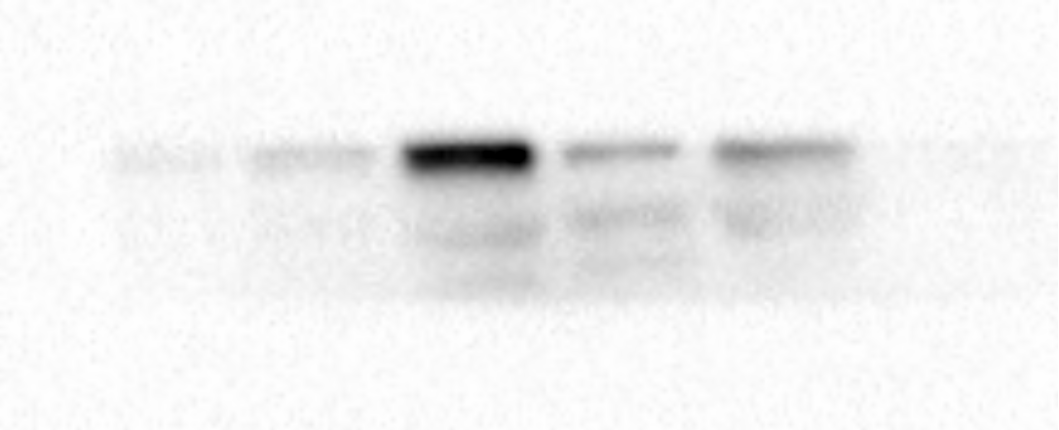

Supplement: Supplementary file 7 [file DataSheet6.zip › 4、IL-6 knockout mouse and WT mouse WB strips/STAT1-7.tif]

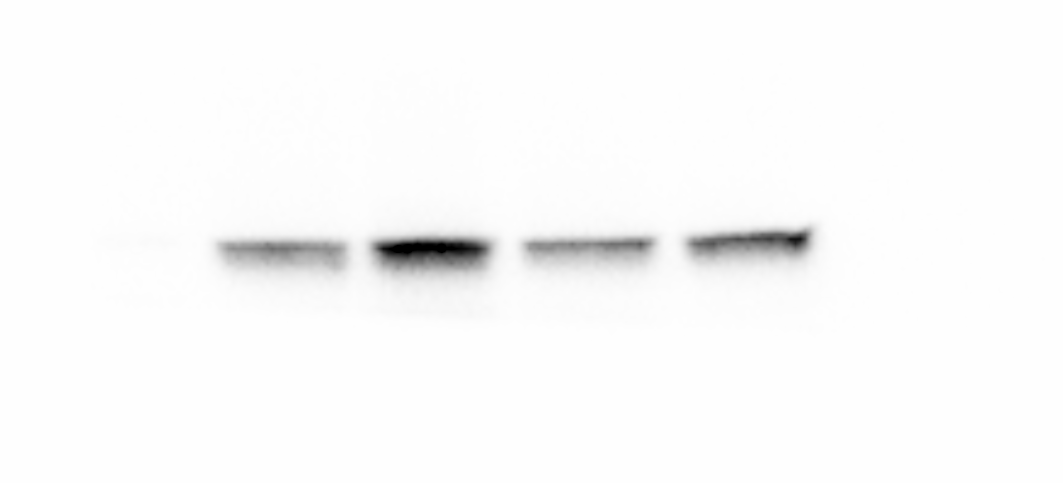

Supplement: Supplementary file 7 [file DataSheet6.zip › 4、IL-6 knockout mouse and WT mouse WB strips/STAT3-2.tif]
