# Supplementary figures and images for: The role and impact of the IL-6 mediated JAK2-STAT1/3 signaling pathway in the pathogenesis of gout (part 2 of 2)
Source: Front Pharmacol. 2025 Mar 18;16:1480844. doi: 10.3389/fphar.2025.1480844 (PMC11959054; doi:10.3389/fphar.2025.1480844)

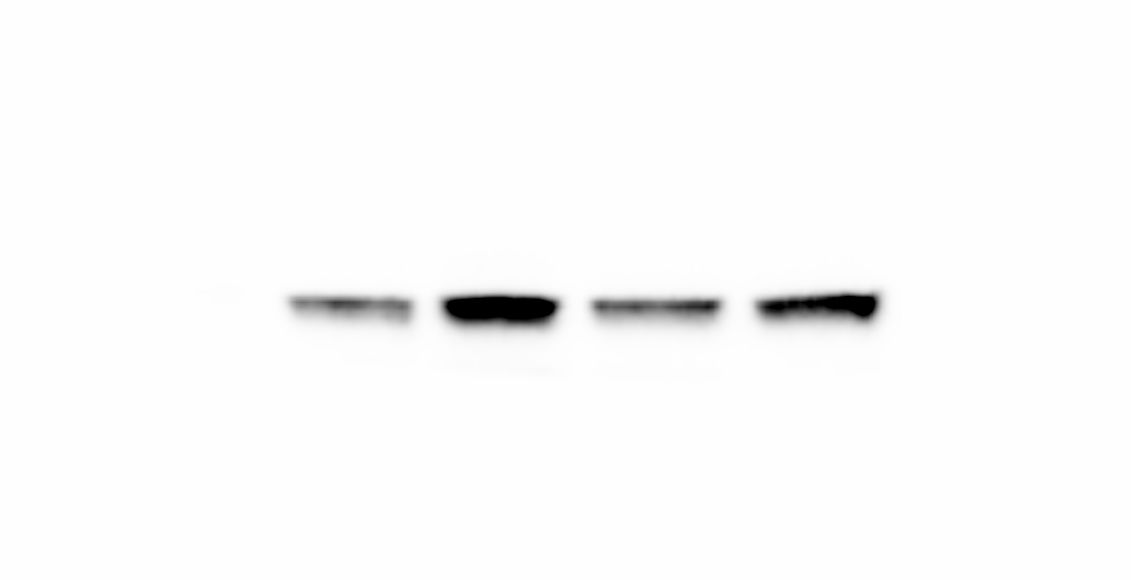

Supplement: Supplementary file 7 [file DataSheet6.zip › 4、IL-6 knockout mouse and WT mouse WB strips/STAT3-3.tif]

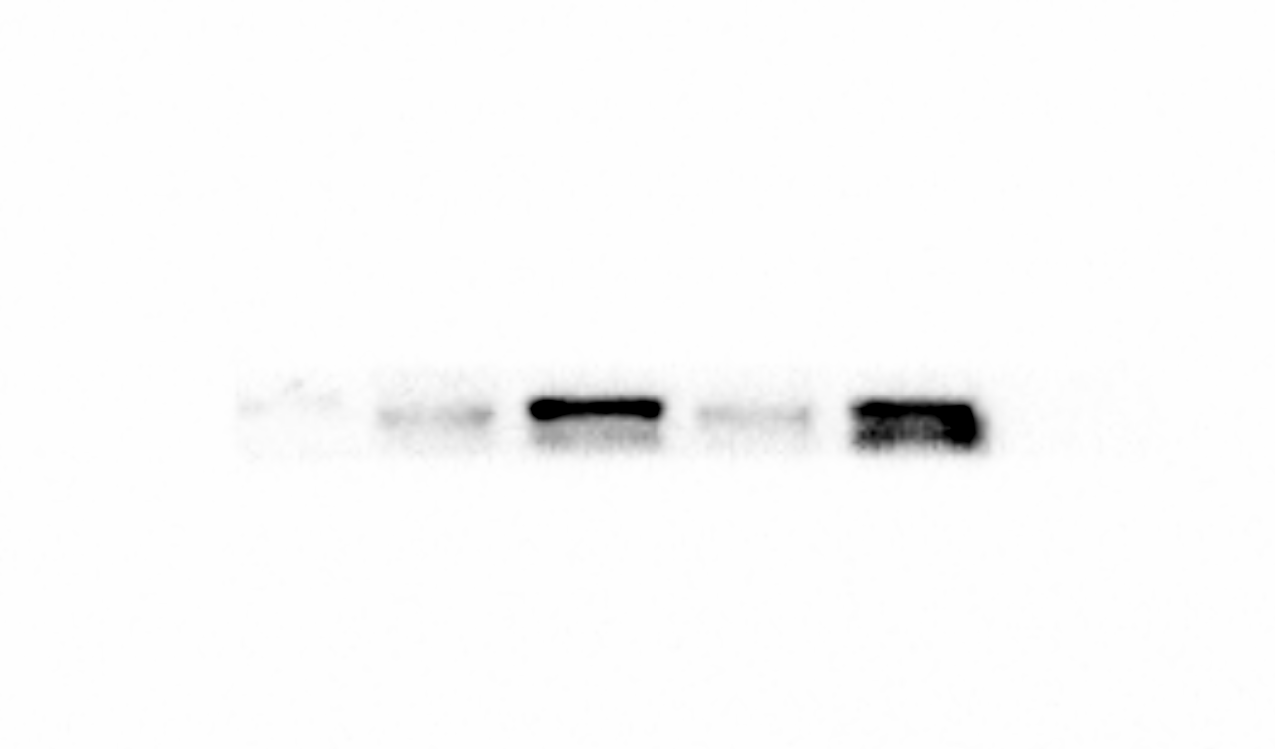

Supplement: Supplementary file 7 [file DataSheet6.zip › 4、IL-6 knockout mouse and WT mouse WB strips/STAT3-4.tif]

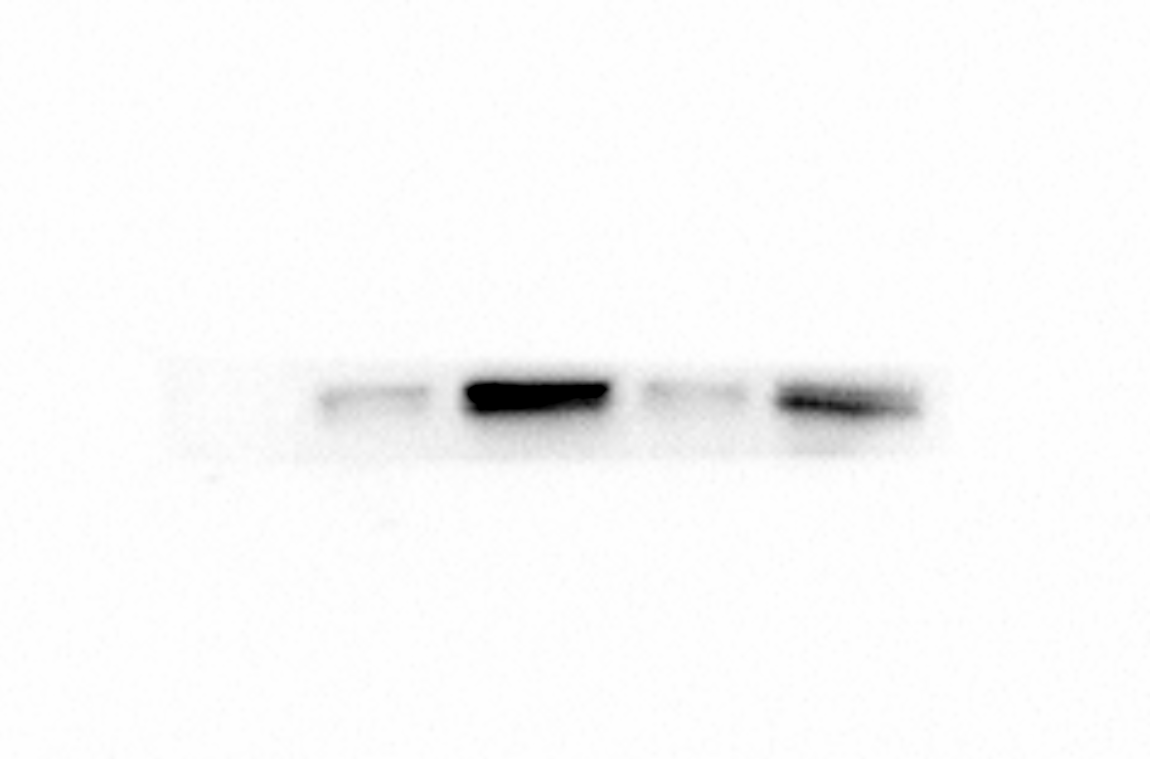

Supplement: Supplementary file 7 [file DataSheet6.zip › 4、IL-6 knockout mouse and WT mouse WB strips/STAT3-5.tif]

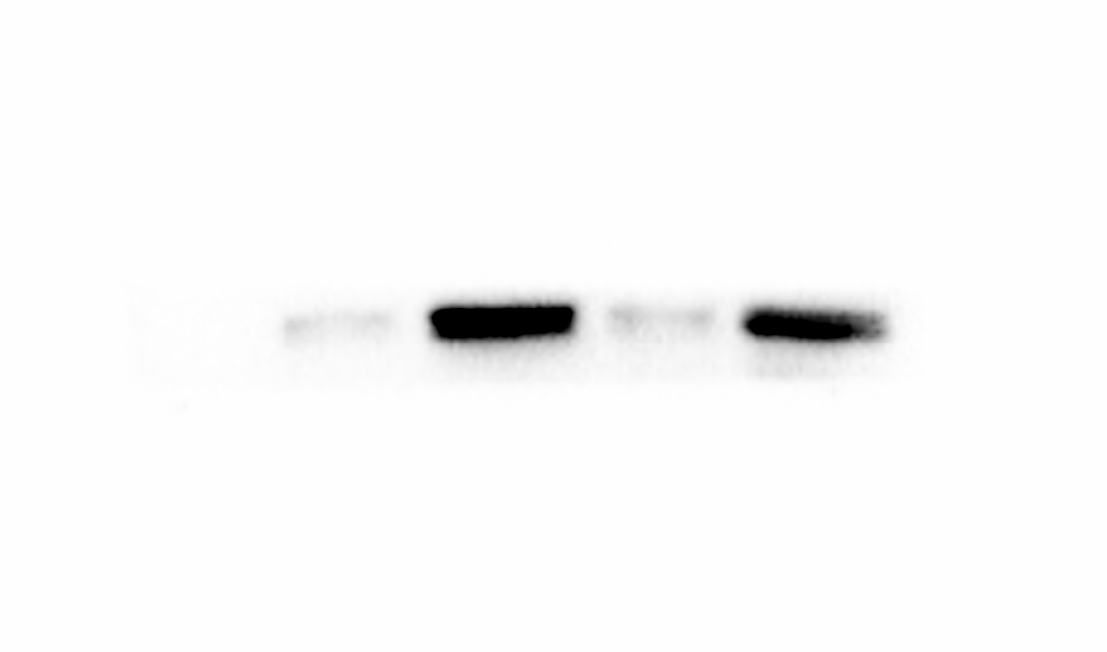

Supplement: Supplementary file 7 [file DataSheet6.zip › 4、IL-6 knockout mouse and WT mouse WB strips/STAT3-6.tif]

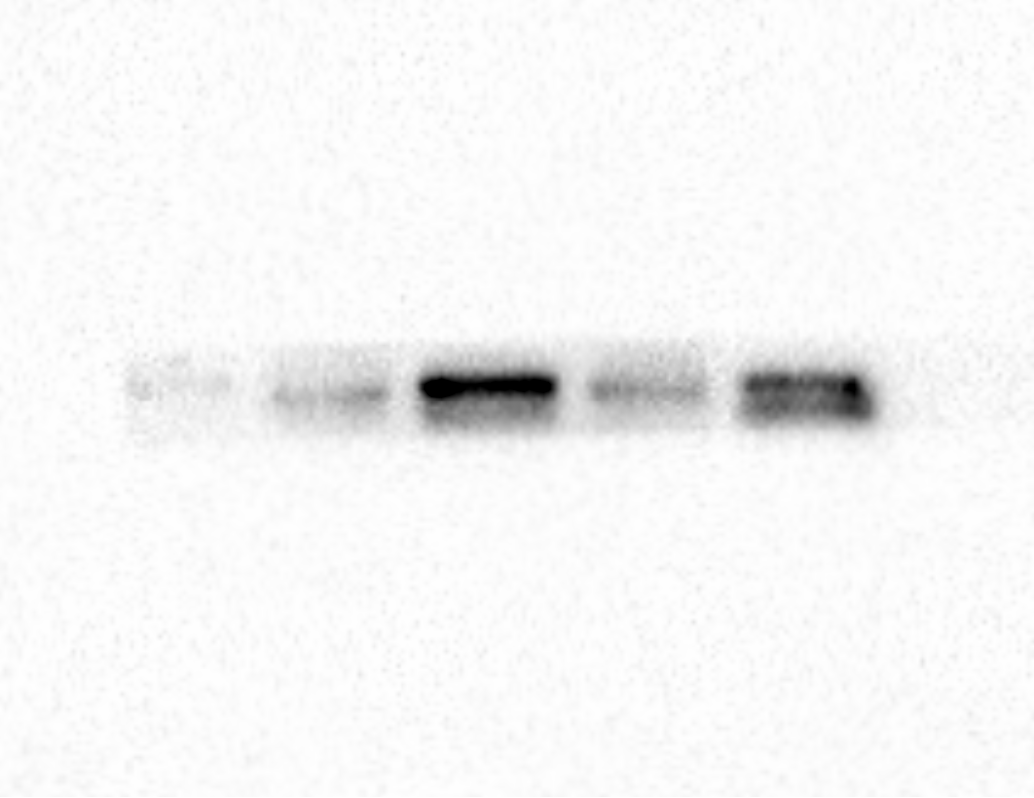

Supplement: Supplementary file 7 [file DataSheet6.zip › 4、IL-6 knockout mouse and WT mouse WB strips/STAT3-7.tif]

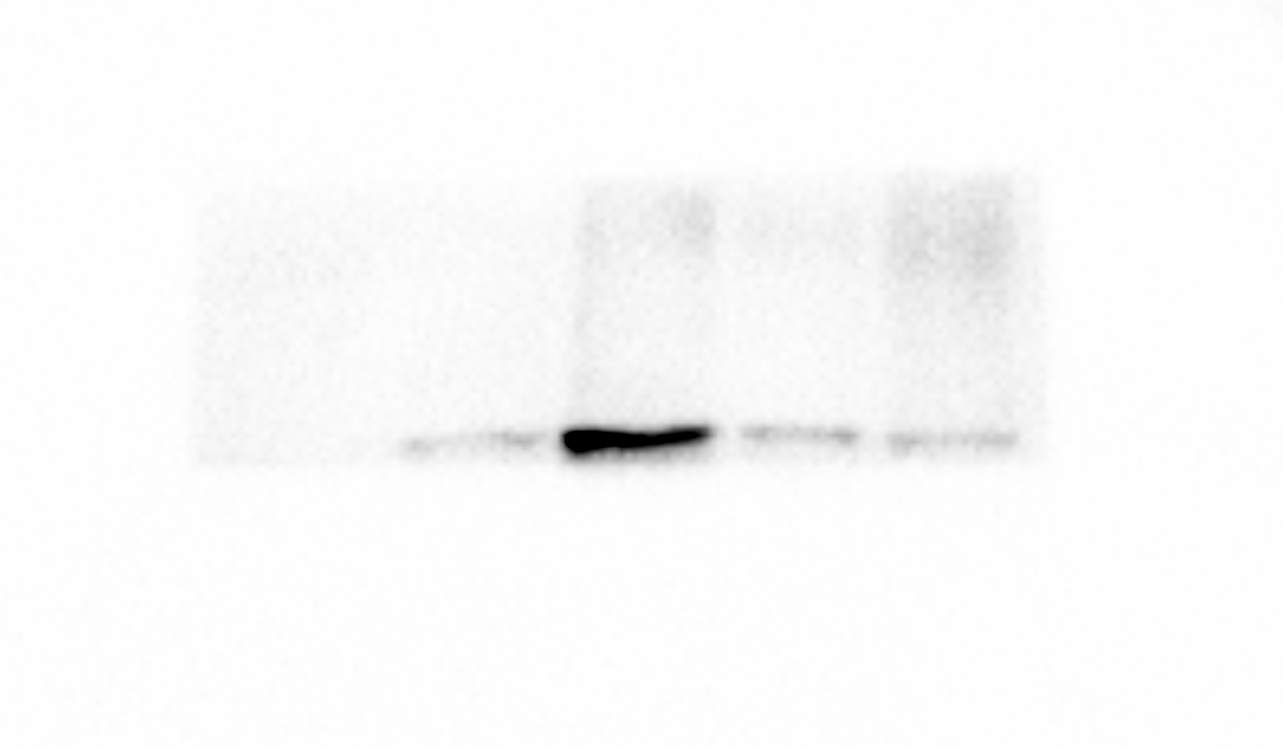

Supplement: Supplementary file 7 [file DataSheet6.zip › 4、IL-6 knockout mouse and WT mouse WB strips/p-STAT1-2.tif]

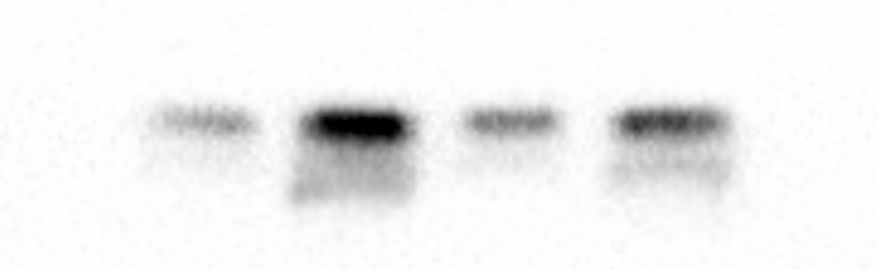

Supplement: Supplementary file 7 [file DataSheet6.zip › 4、IL-6 knockout mouse and WT mouse WB strips/p-STAT3-15-1.tif]

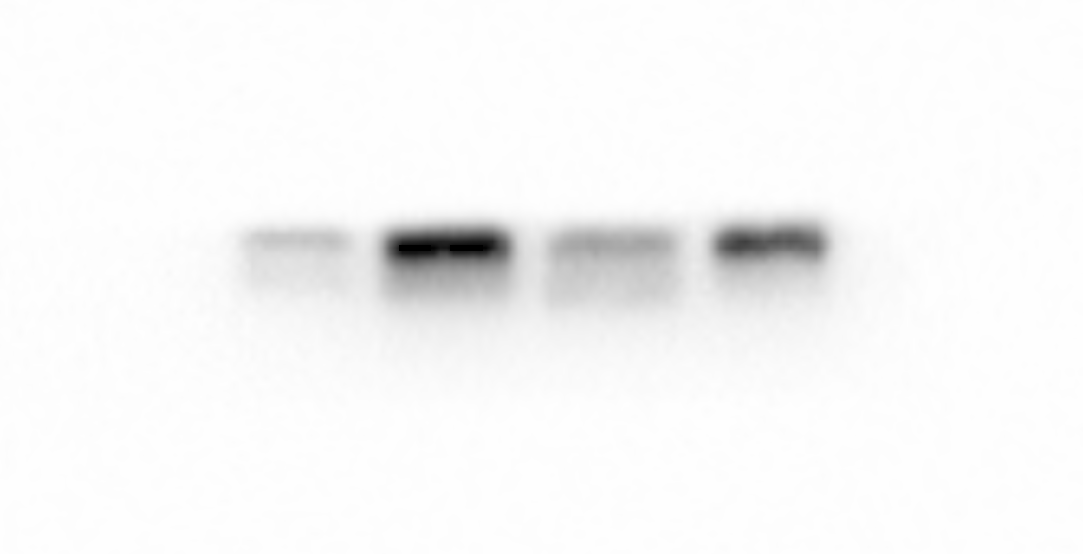

Supplement: Supplementary file 7 [file DataSheet6.zip › 4、IL-6 knockout mouse and WT mouse WB strips/p-STAT3-2.tif]

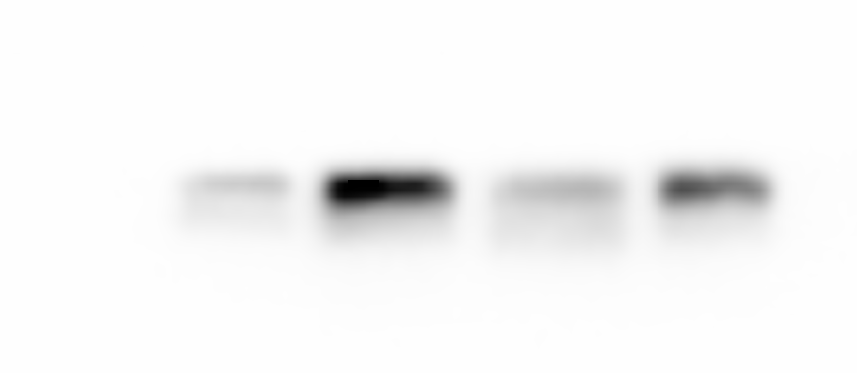

Supplement: Supplementary file 7 [file DataSheet6.zip › 4、IL-6 knockout mouse and WT mouse WB strips/p-STAT3-3.tif]

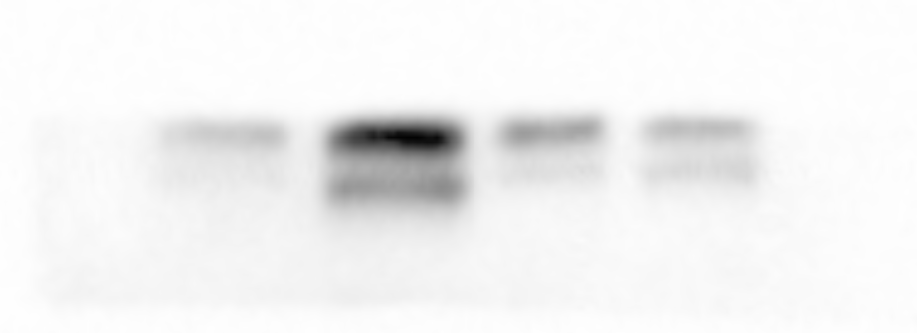

Supplement: Supplementary file 7 [file DataSheet6.zip › 4、IL-6 knockout mouse and WT mouse WB strips/p-STAT3-4.tif]

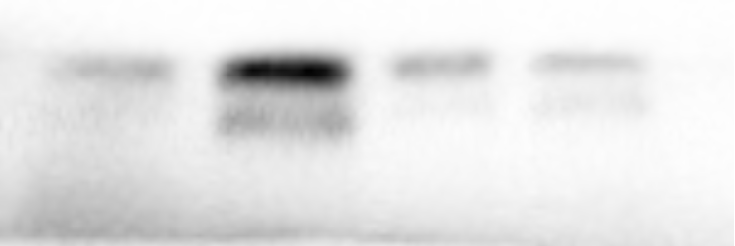

Supplement: Supplementary file 7 [file DataSheet6.zip › 4、IL-6 knockout mouse and WT mouse WB strips/p-STAT3-5.tif]

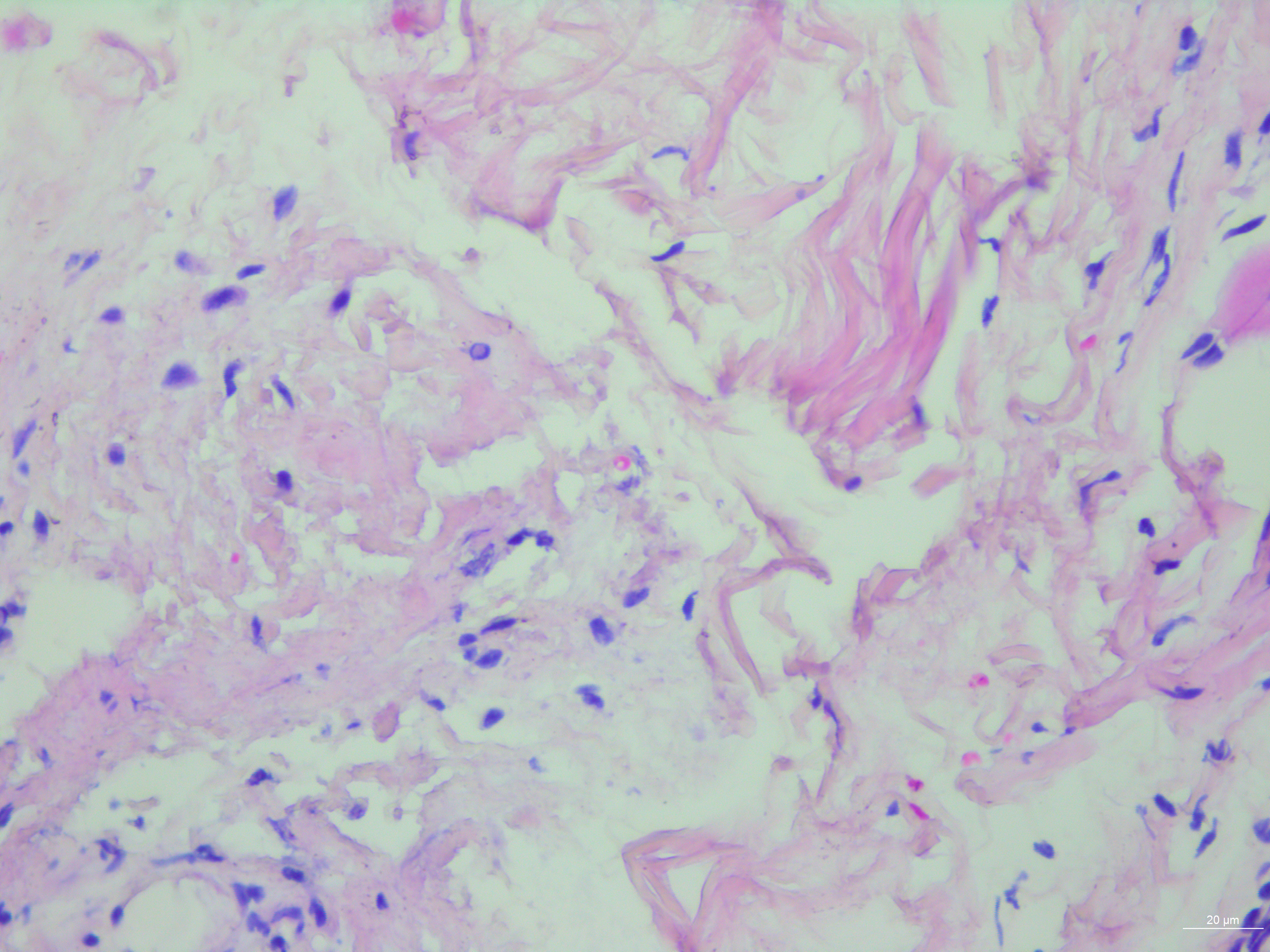

Supplement: Supplementary file 8 [file DataSheet2.zip › IL-6 KO HE staining/IL-6 KO 0h.jpg]

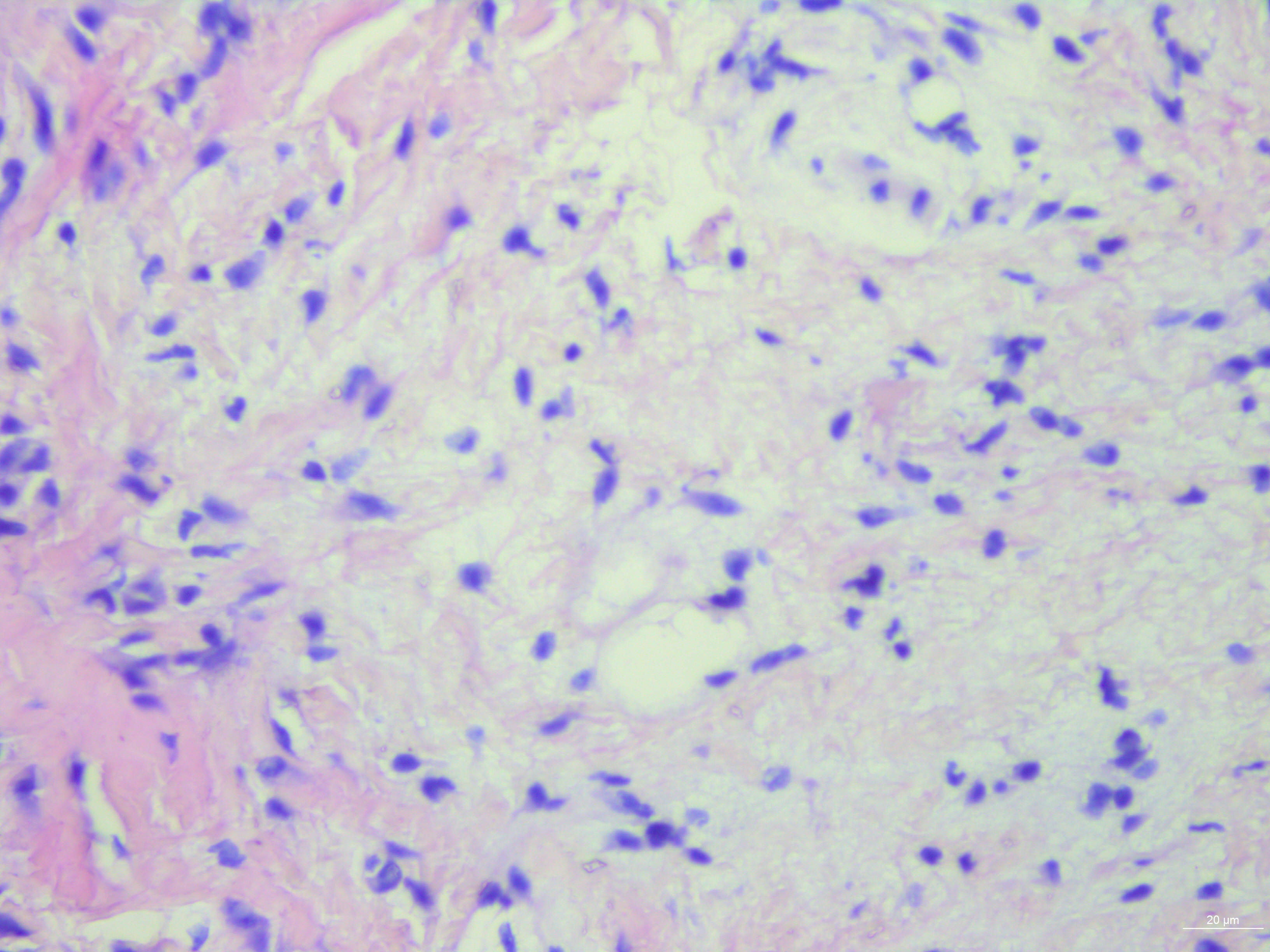

Supplement: Supplementary file 8 [file DataSheet2.zip › IL-6 KO HE staining/IL-6 KO MSU12h.jpg]

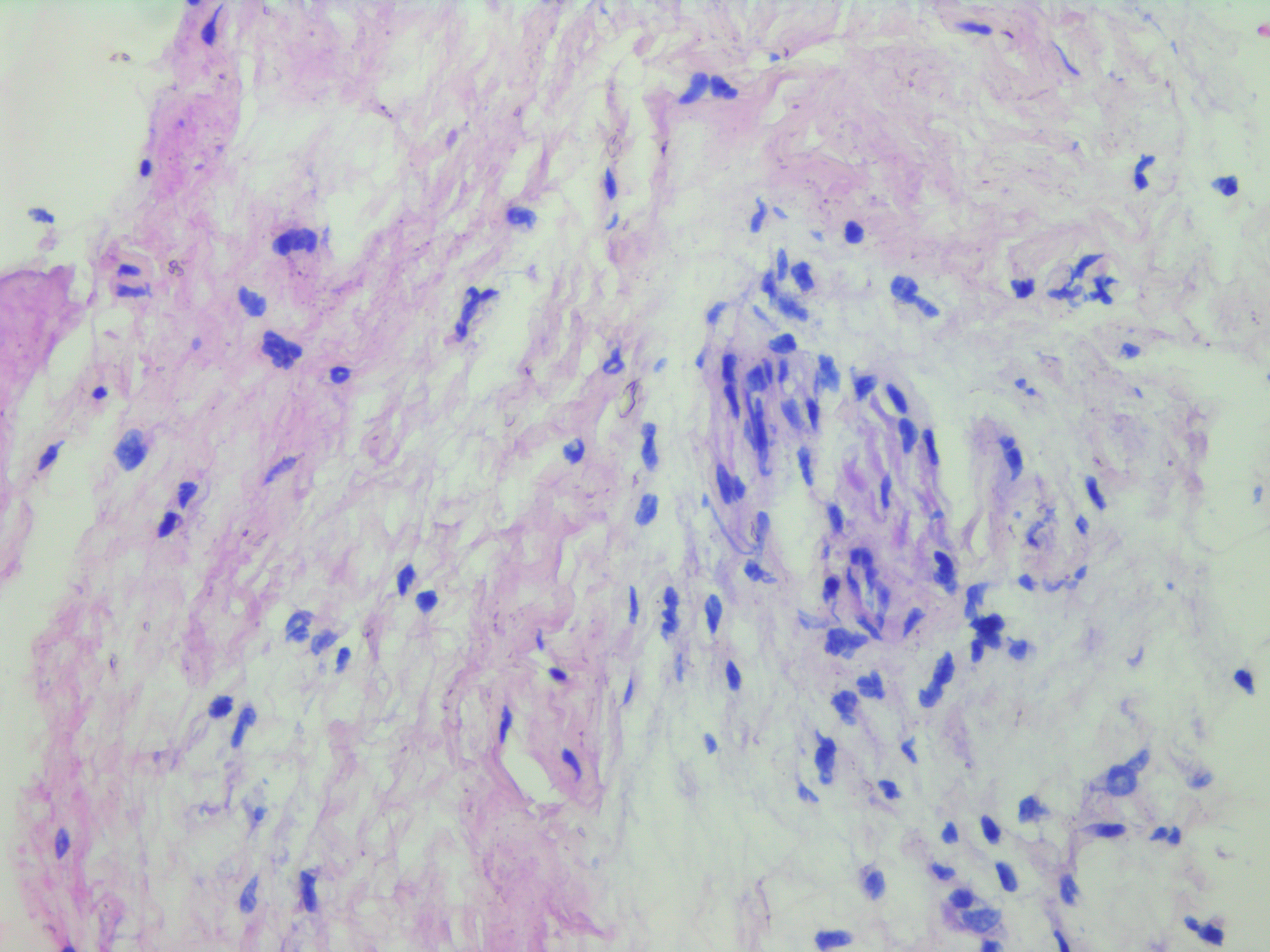

Supplement: Supplementary file 8 [file DataSheet2.zip › IL-6 KO HE staining/IL-6 KO MSU24h.jpg]

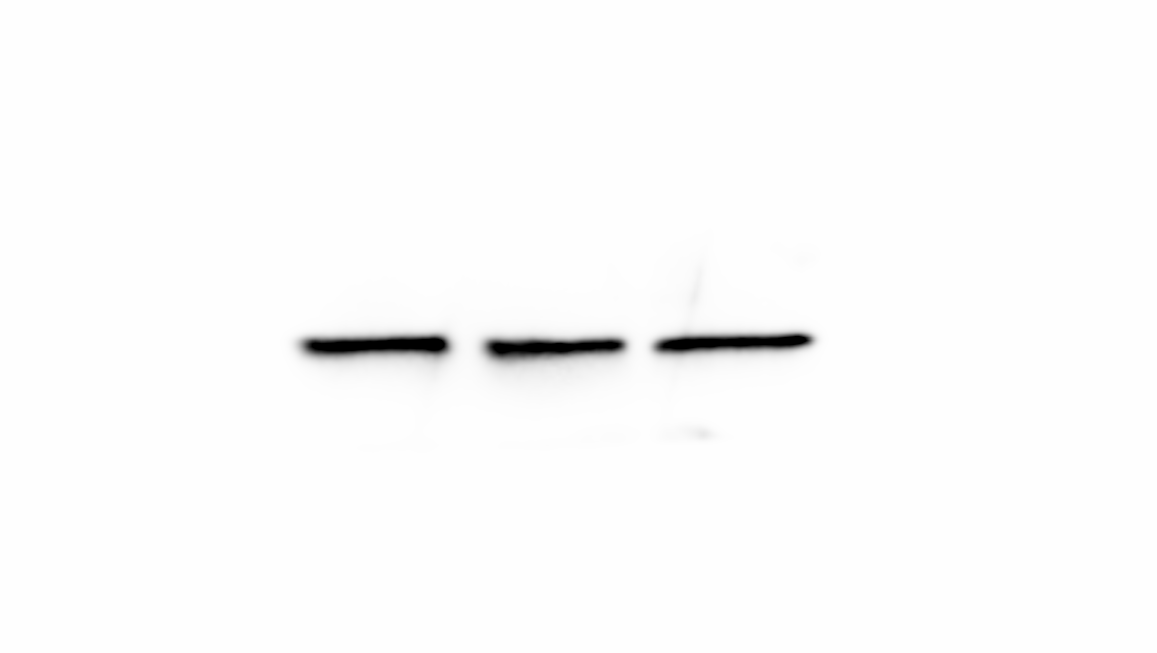

Supplement: Supplementary file 9 [file DataSheet5.zip › 1、WB bands of human blood (AGIGHC)/GAPDH-2.tif]

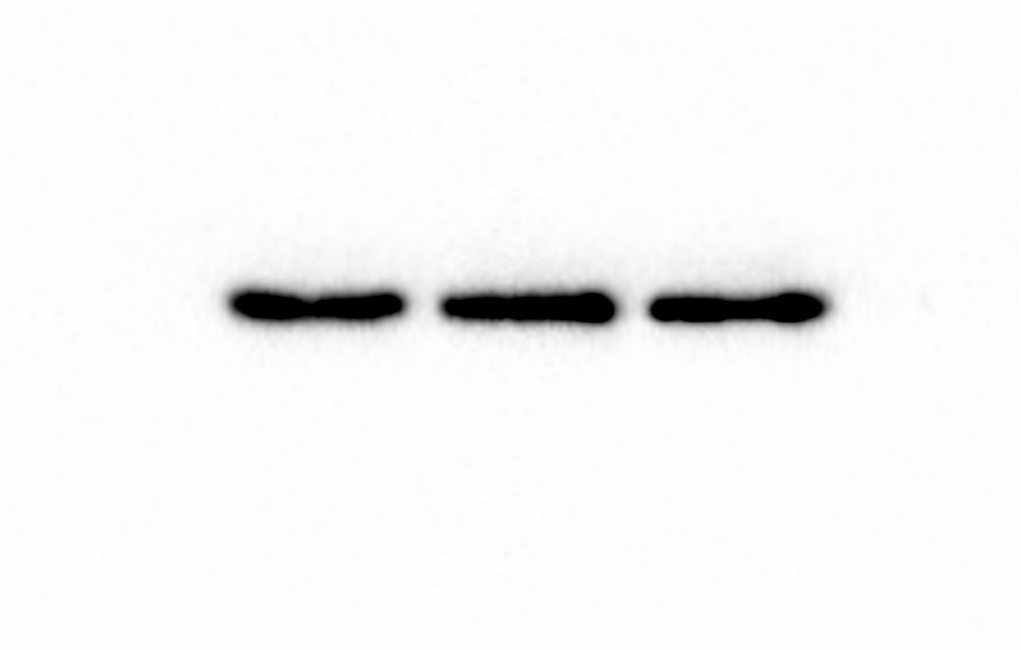

Supplement: Supplementary file 9 [file DataSheet5.zip › 1、WB bands of human blood (AGIGHC)/GAPDH-3.tif]

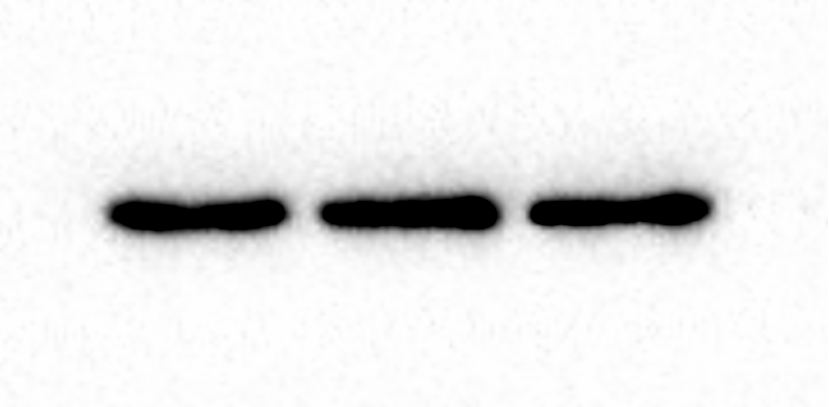

Supplement: Supplementary file 9 [file DataSheet5.zip › 1、WB bands of human blood (AGIGHC)/GAPDH-4.tif]

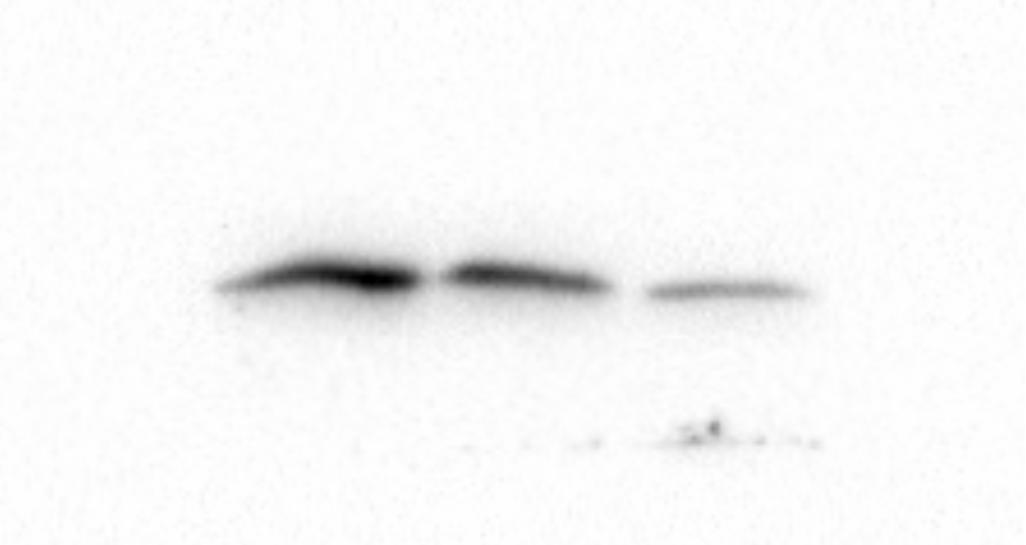

Supplement: Supplementary file 9 [file DataSheet5.zip › 1、WB bands of human blood (AGIGHC)/IL-1β-2.tif]

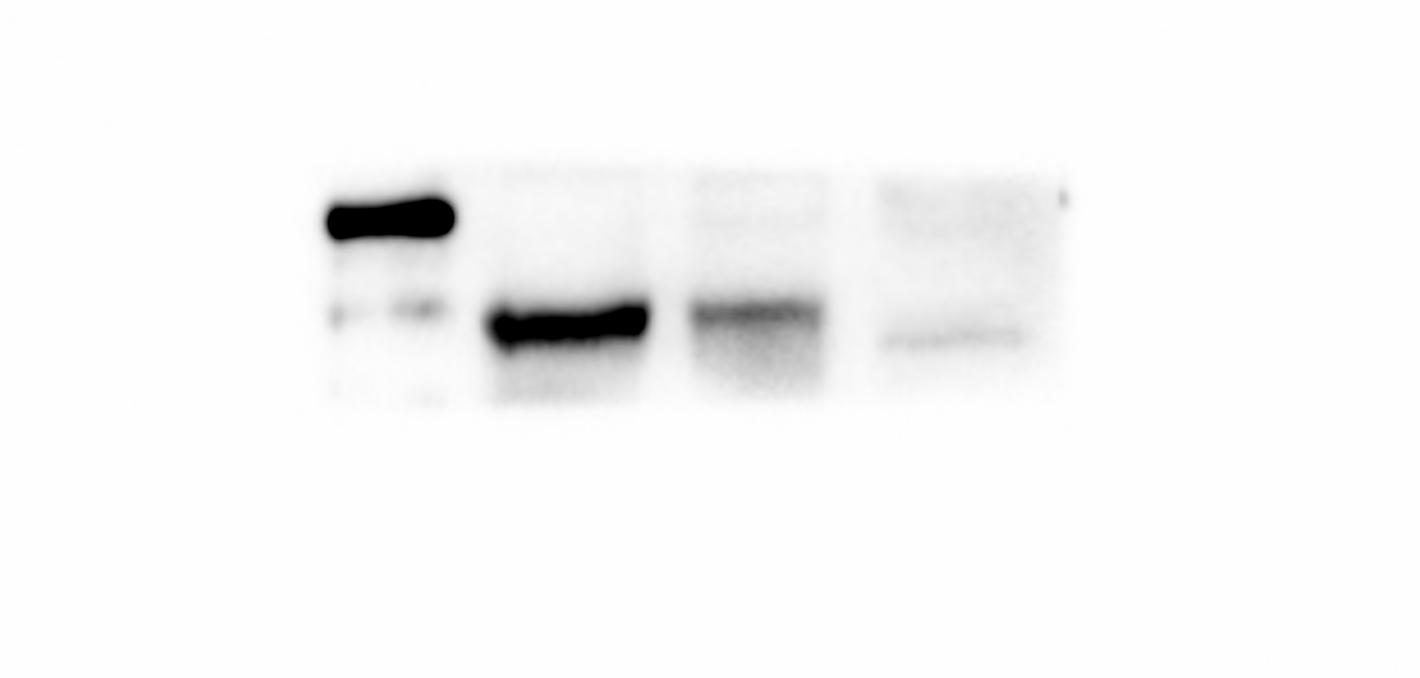

Supplement: Supplementary file 9 [file DataSheet5.zip › 1、WB bands of human blood (AGIGHC)/JAK2-2.tif]

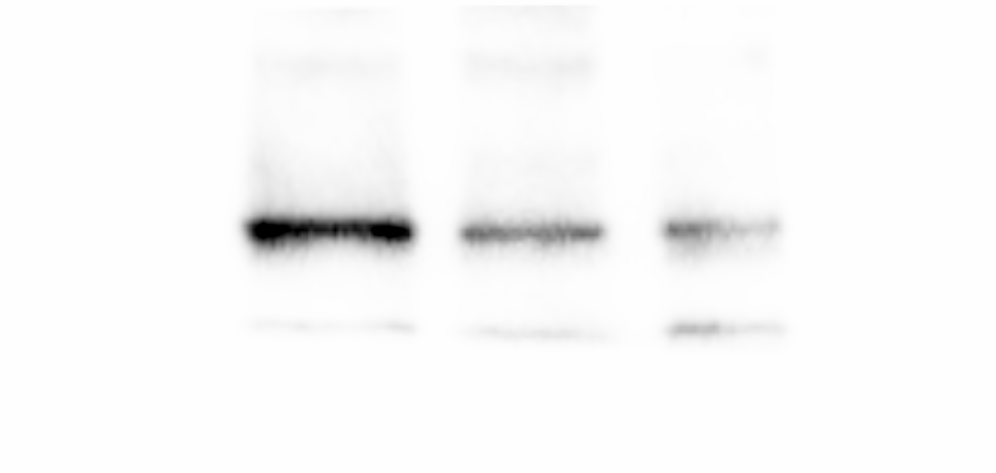

Supplement: Supplementary file 9 [file DataSheet5.zip › 1、WB bands of human blood (AGIGHC)/JAK2-3.tif]

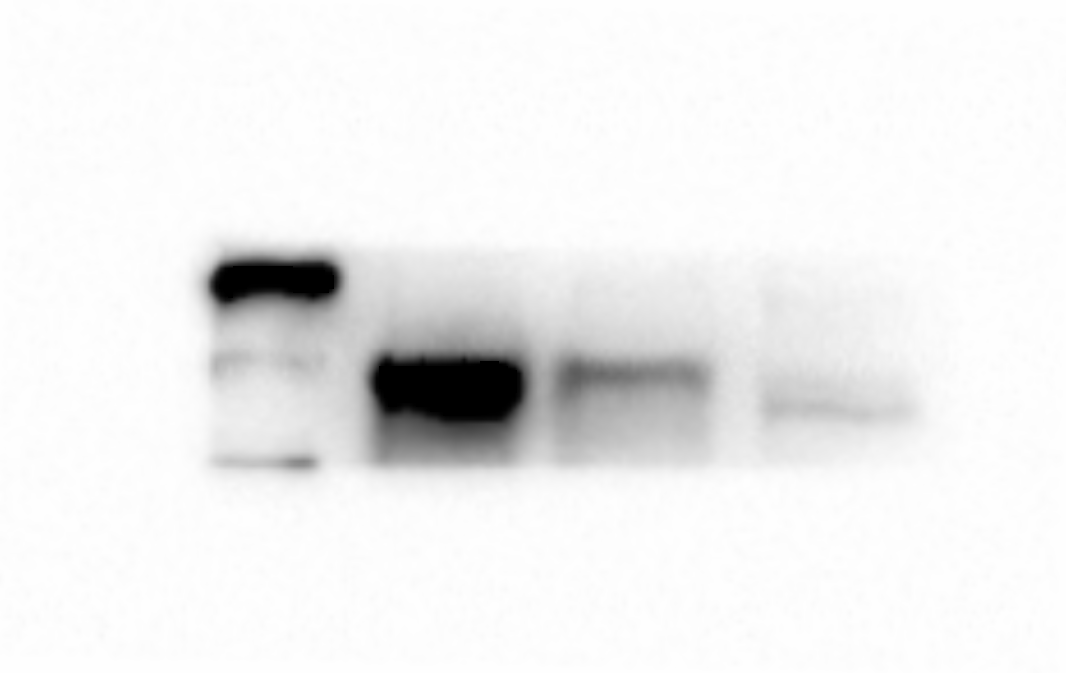

Supplement: Supplementary file 9 [file DataSheet5.zip › 1、WB bands of human blood (AGIGHC)/JAK2-4.tif]

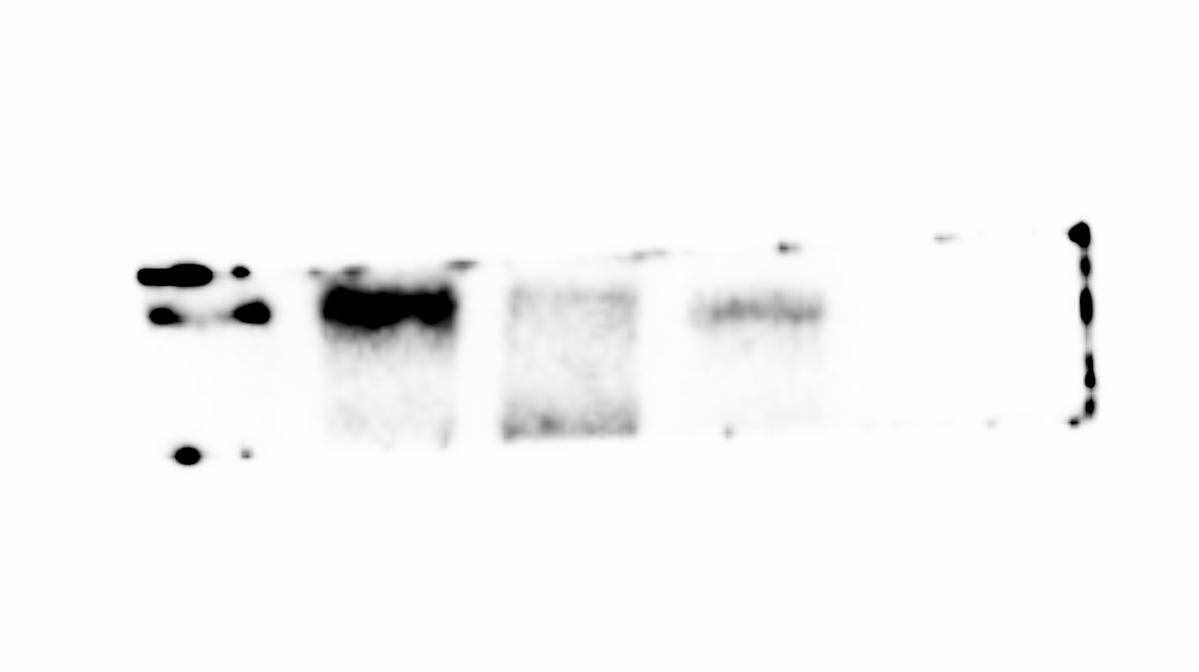

Supplement: Supplementary file 9 [file DataSheet5.zip › 1、WB bands of human blood (AGIGHC)/P-JAK2-4.tif]

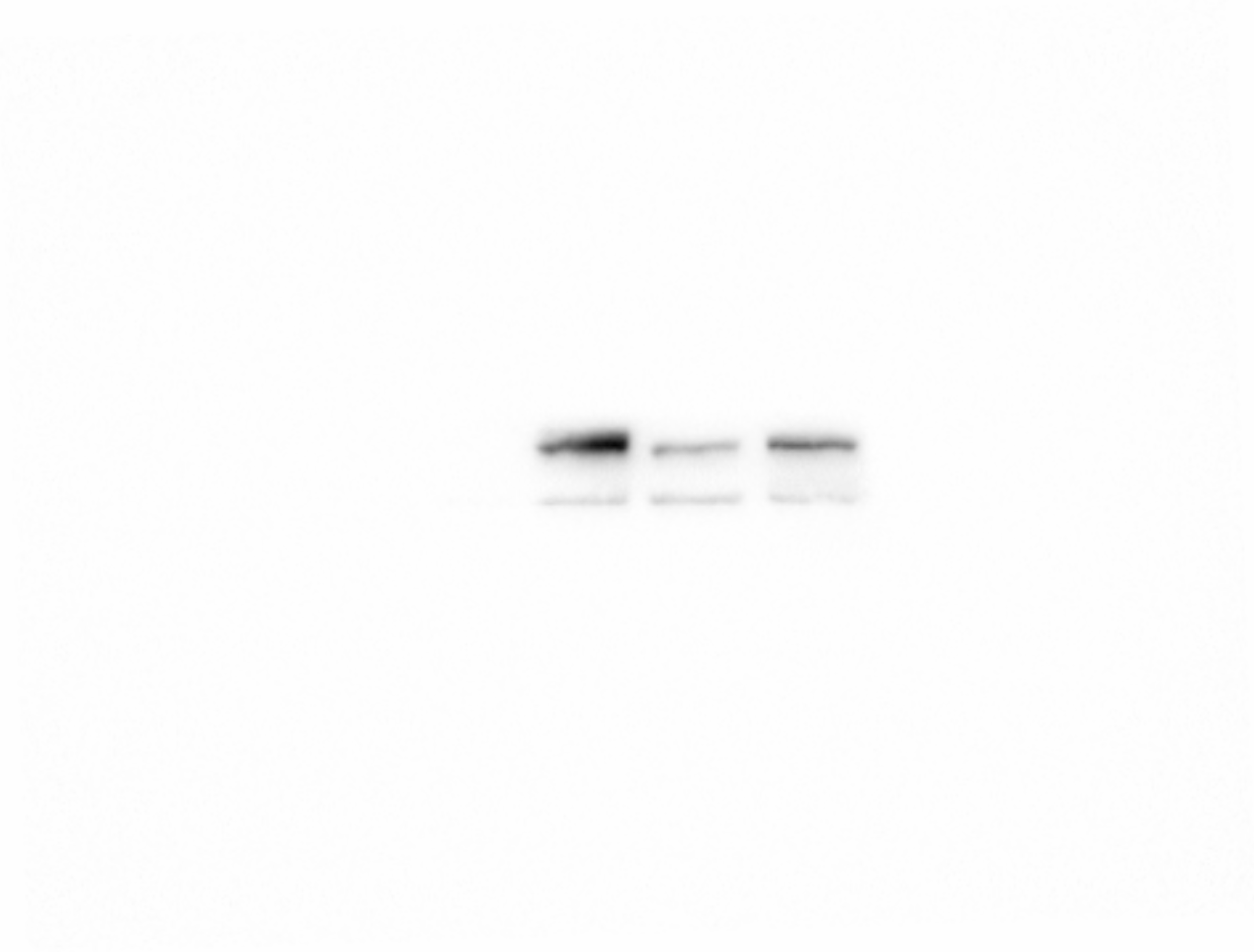

Supplement: Supplementary file 9 [file DataSheet5.zip › 1、WB bands of human blood (AGIGHC)/STAT1-2.tif]

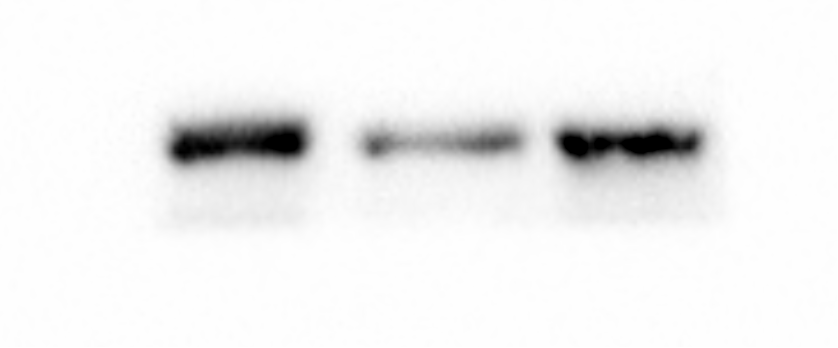

Supplement: Supplementary file 9 [file DataSheet5.zip › 1、WB bands of human blood (AGIGHC)/STAT1-4.tif]

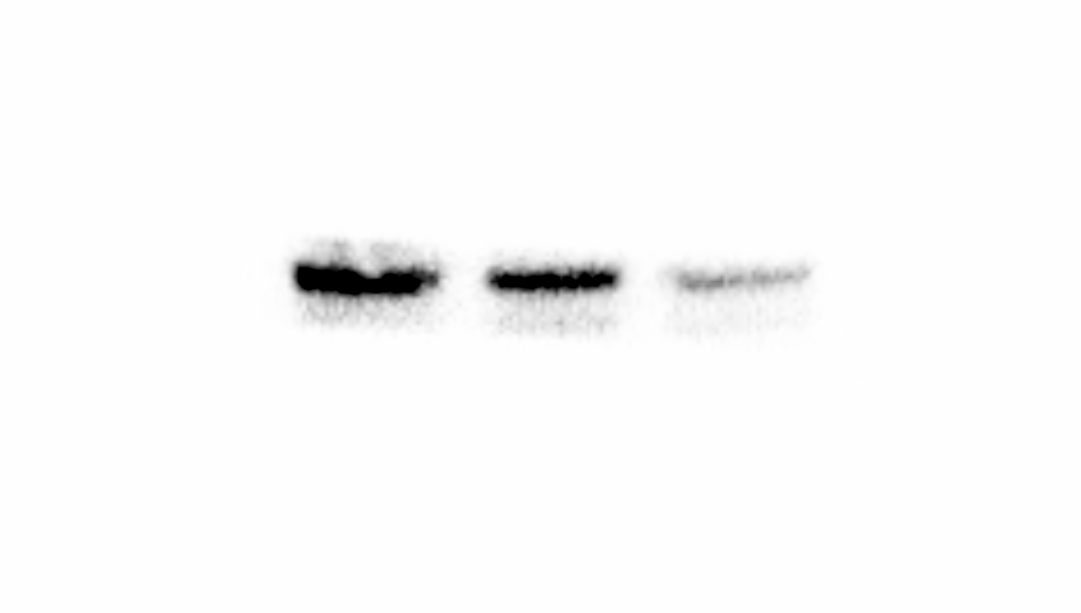

Supplement: Supplementary file 9 [file DataSheet5.zip › 1、WB bands of human blood (AGIGHC)/STAT3-2.tif]

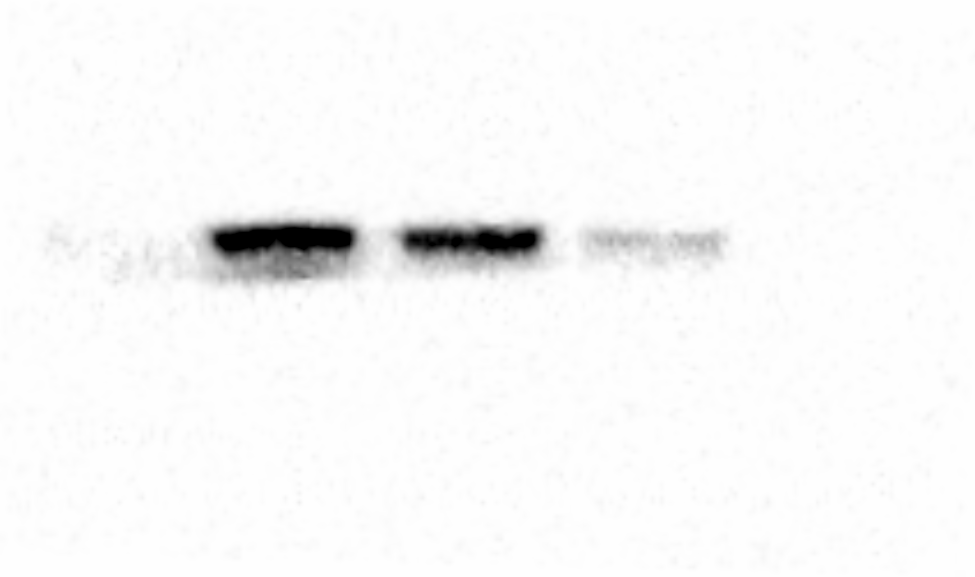

Supplement: Supplementary file 9 [file DataSheet5.zip › 1、WB bands of human blood (AGIGHC)/STAT3-4.tif]

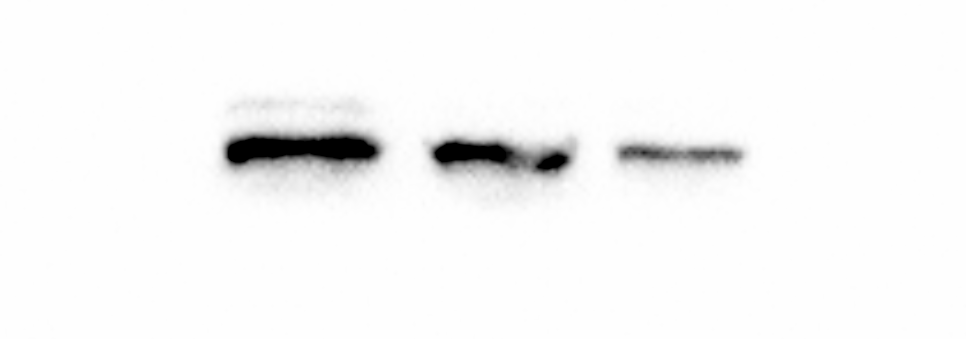

Supplement: Supplementary file 9 [file DataSheet5.zip › 1、WB bands of human blood (AGIGHC)/STAT3-5.tif]

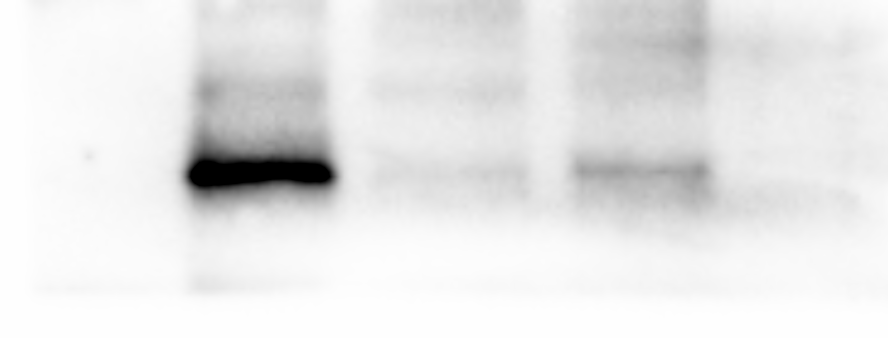

Supplement: Supplementary file 9 [file DataSheet5.zip › 1、WB bands of human blood (AGIGHC)/p-JAK2-2.tif]

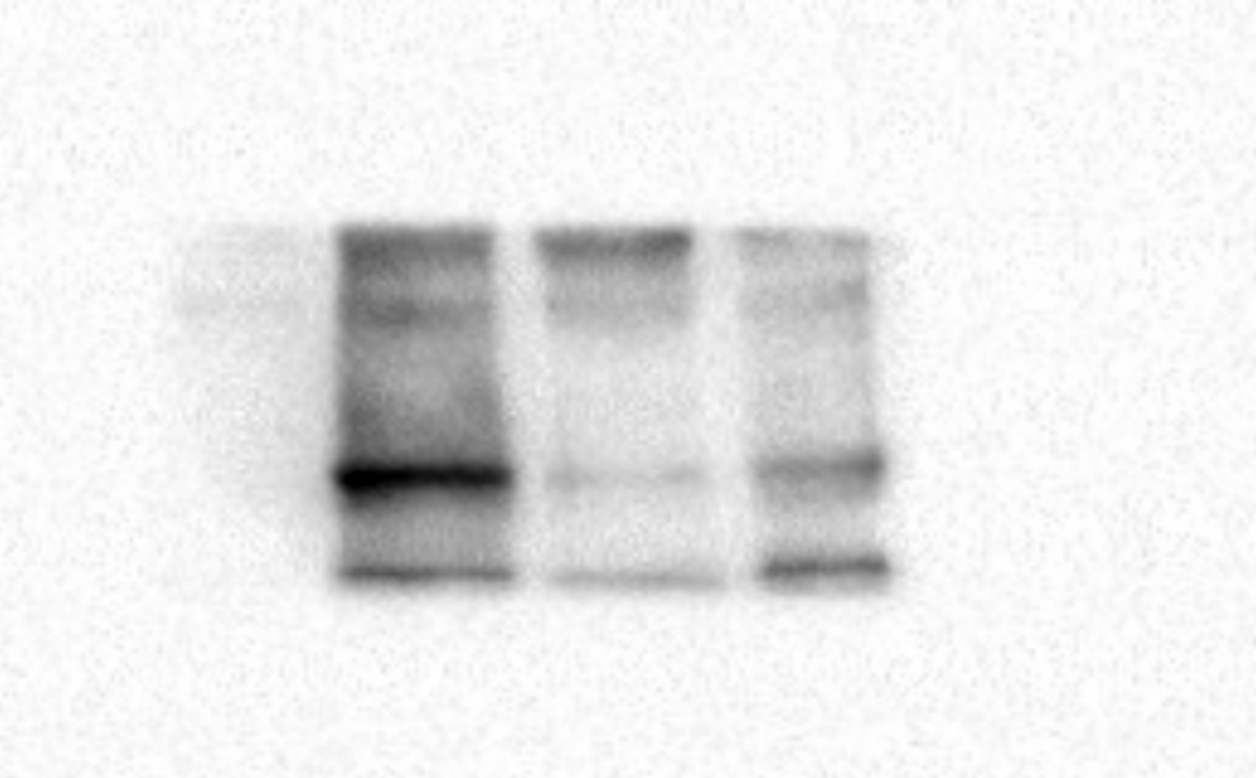

Supplement: Supplementary file 9 [file DataSheet5.zip › 1、WB bands of human blood (AGIGHC)/p-JAK2-3.tif]

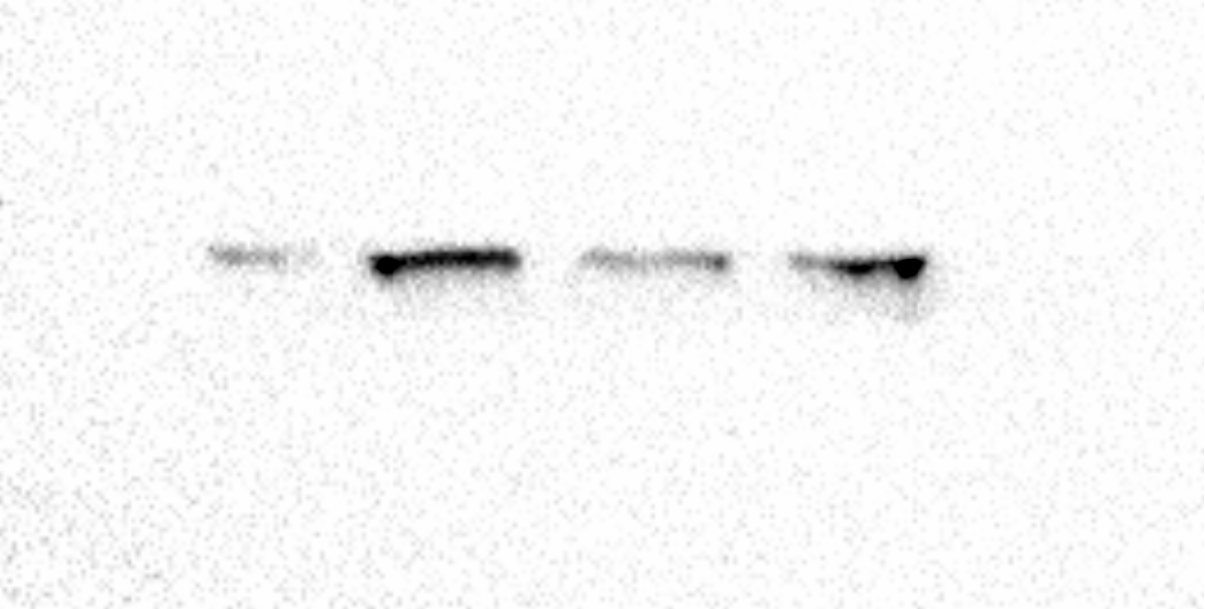

Supplement: Supplementary file 9 [file DataSheet5.zip › 1、WB bands of human blood (AGIGHC)/p-STAT1-2.tif]

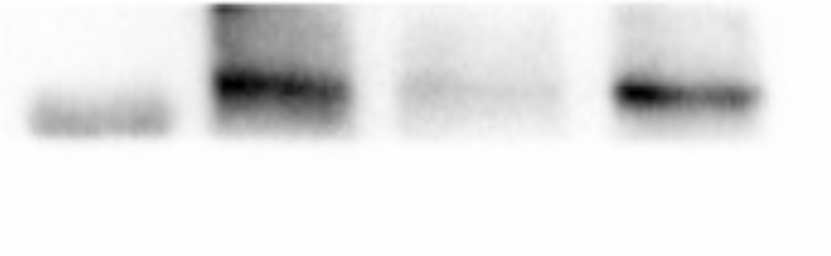

Supplement: Supplementary file 9 [file DataSheet5.zip › 1、WB bands of human blood (AGIGHC)/p-STAT1-3.tif]

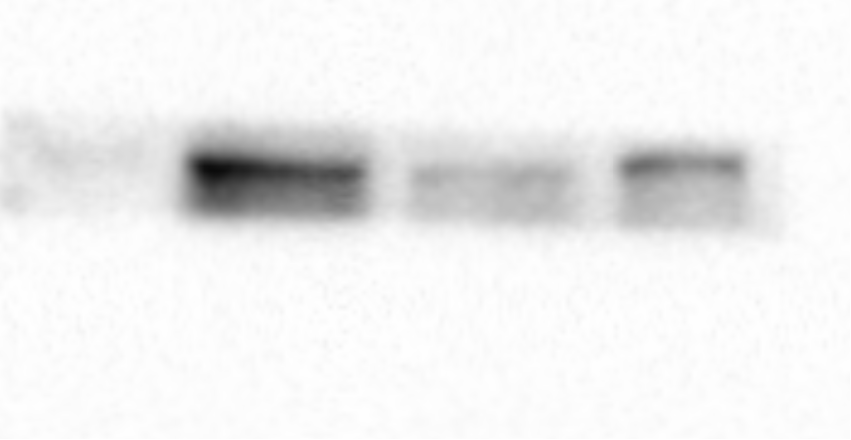

Supplement: Supplementary file 9 [file DataSheet5.zip › 1、WB bands of human blood (AGIGHC)/p-STAT1-4.tif]

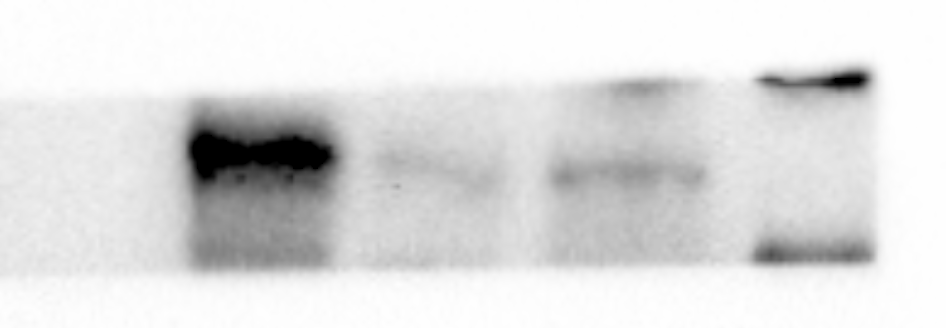

Supplement: Supplementary file 9 [file DataSheet5.zip › 1、WB bands of human blood (AGIGHC)/p-STAT3-3.tif]

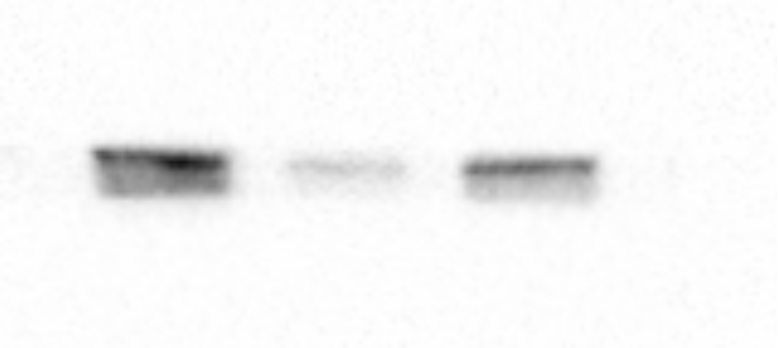

Supplement: Supplementary file 9 [file DataSheet5.zip › 1、WB bands of human blood (AGIGHC)/p-STAT3-4.tif]

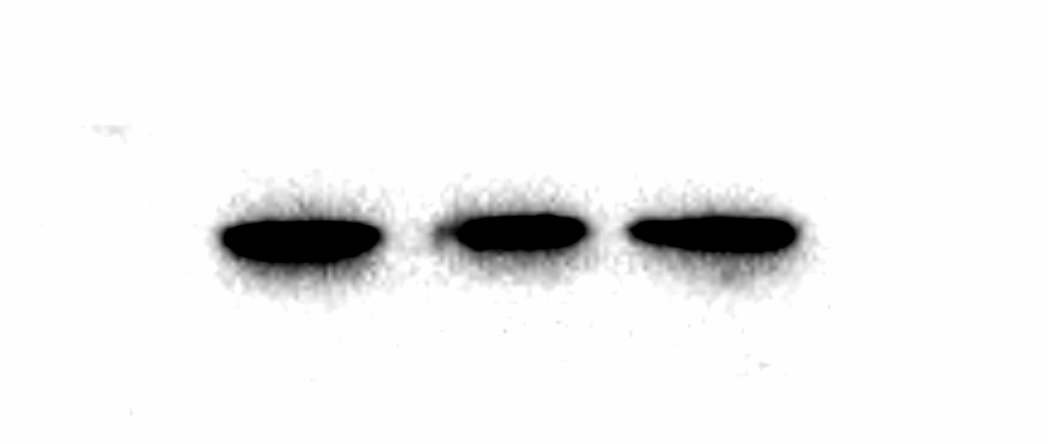

Supplement: Supplementary file 10 [file DataSheet7.zip › 3、WB bands from THP-1 cell gout model/GAPDH-2.tif]

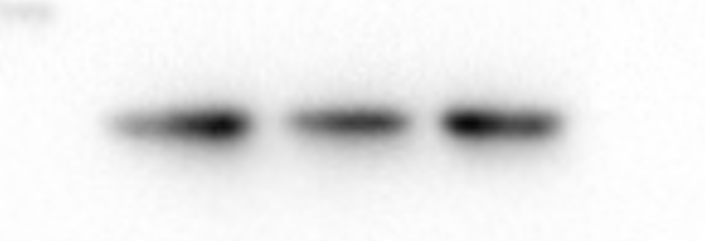

Supplement: Supplementary file 10 [file DataSheet7.zip › 3、WB bands from THP-1 cell gout model/GAPDH-3.tif]

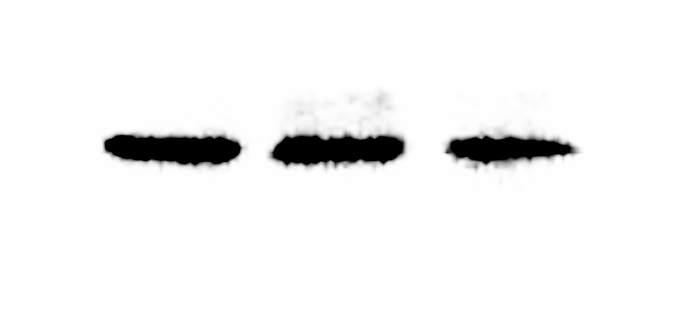

Supplement: Supplementary file 10 [file DataSheet7.zip › 3、WB bands from THP-1 cell gout model/GAPDH-4.tif]

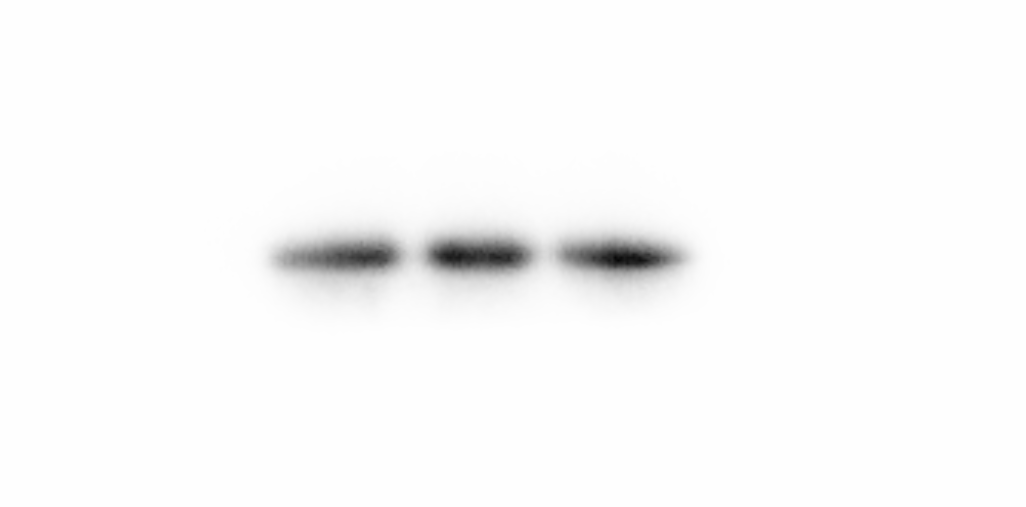

Supplement: Supplementary file 10 [file DataSheet7.zip › 3、WB bands from THP-1 cell gout model/GAPDH-5.tif]

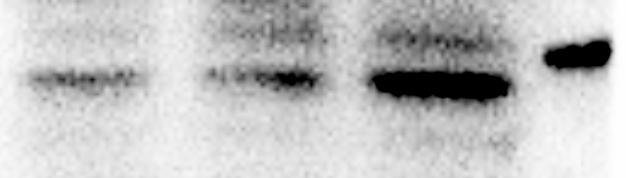

Supplement: Supplementary file 10 [file DataSheet7.zip › 3、WB bands from THP-1 cell gout model/IL-1β-2.tif]

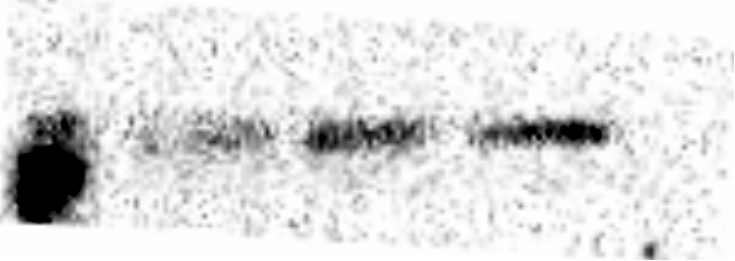

Supplement: Supplementary file 10 [file DataSheet7.zip › 3、WB bands from THP-1 cell gout model/IL-1β-3.tif]

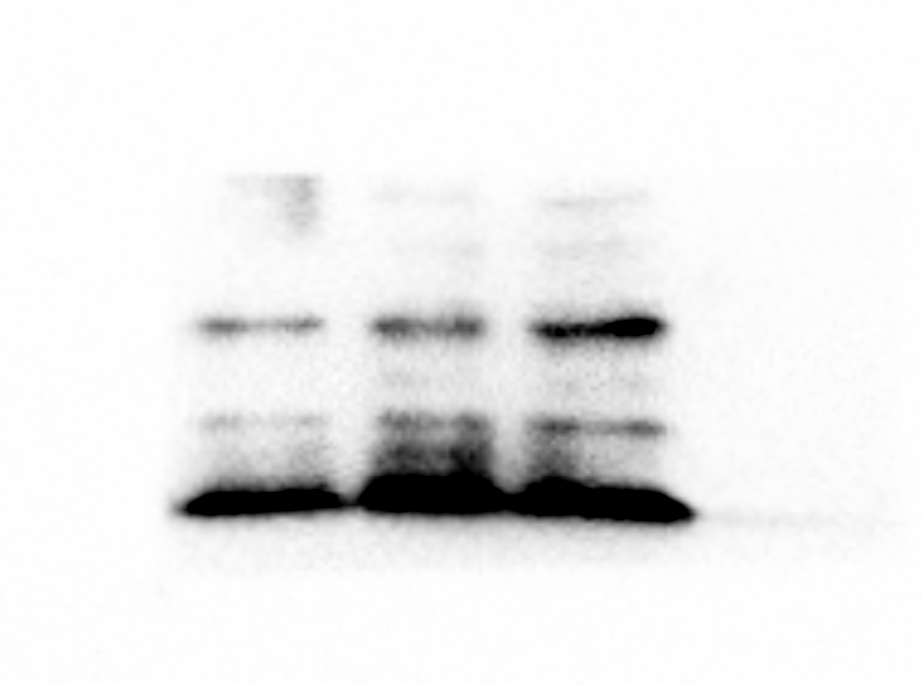

Supplement: Supplementary file 10 [file DataSheet7.zip › 3、WB bands from THP-1 cell gout model/IL-1β-4.tif]

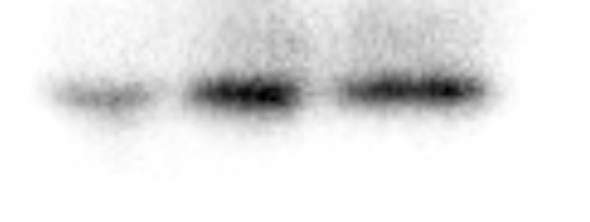

Supplement: Supplementary file 10 [file DataSheet7.zip › 3、WB bands from THP-1 cell gout model/IL6-2.tif]

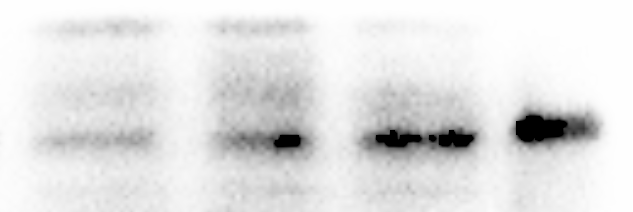

Supplement: Supplementary file 10 [file DataSheet7.zip › 3、WB bands from THP-1 cell gout model/IL6-3.tif]

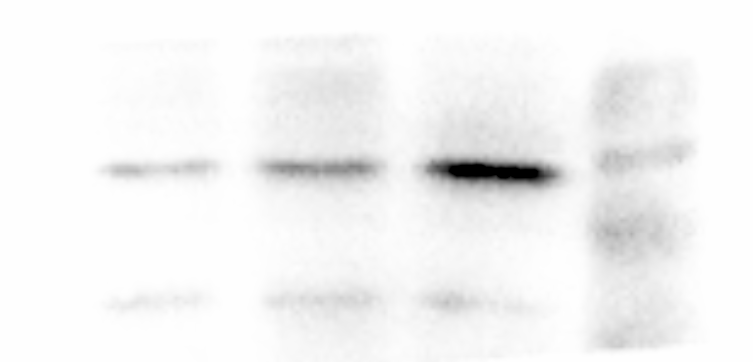

Supplement: Supplementary file 10 [file DataSheet7.zip › 3、WB bands from THP-1 cell gout model/IL6-4.tif]

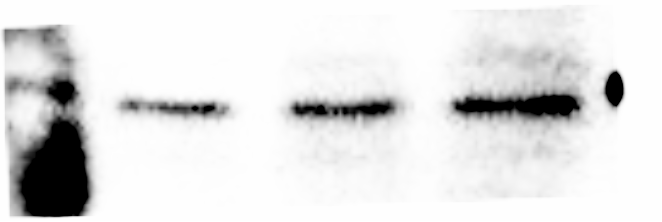

Supplement: Supplementary file 10 [file DataSheet7.zip › 3、WB bands from THP-1 cell gout model/IL6-5.tif]

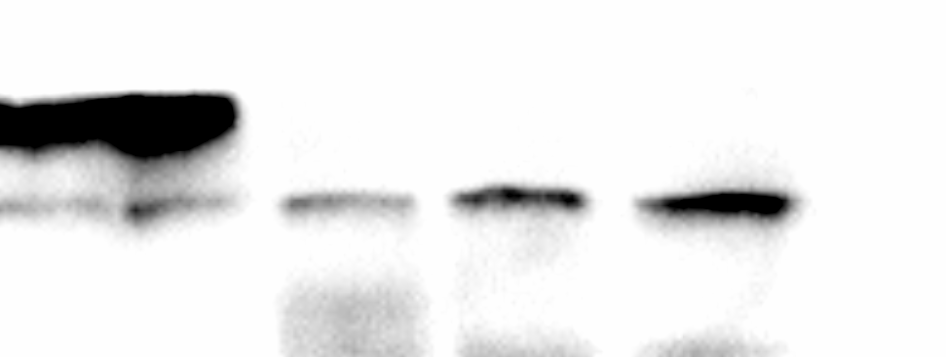

Supplement: Supplementary file 10 [file DataSheet7.zip › 3、WB bands from THP-1 cell gout model/JAK2-2.tif]

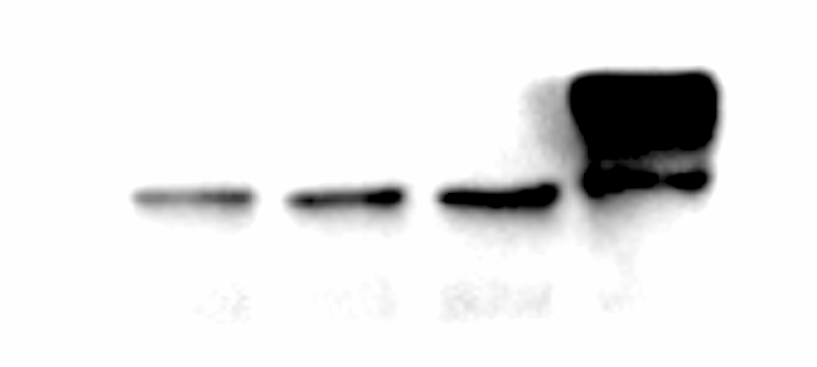

Supplement: Supplementary file 10 [file DataSheet7.zip › 3、WB bands from THP-1 cell gout model/JAK2-3.tif]

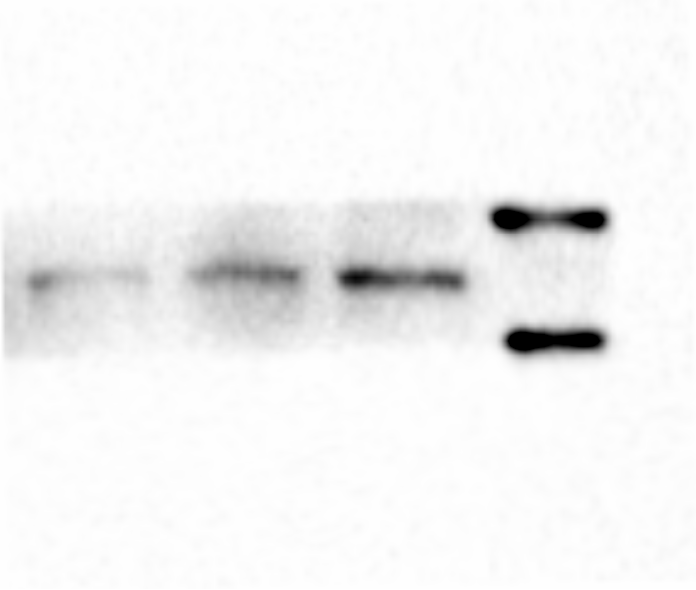

Supplement: Supplementary file 10 [file DataSheet7.zip › 3、WB bands from THP-1 cell gout model/JAK2-4.tif]

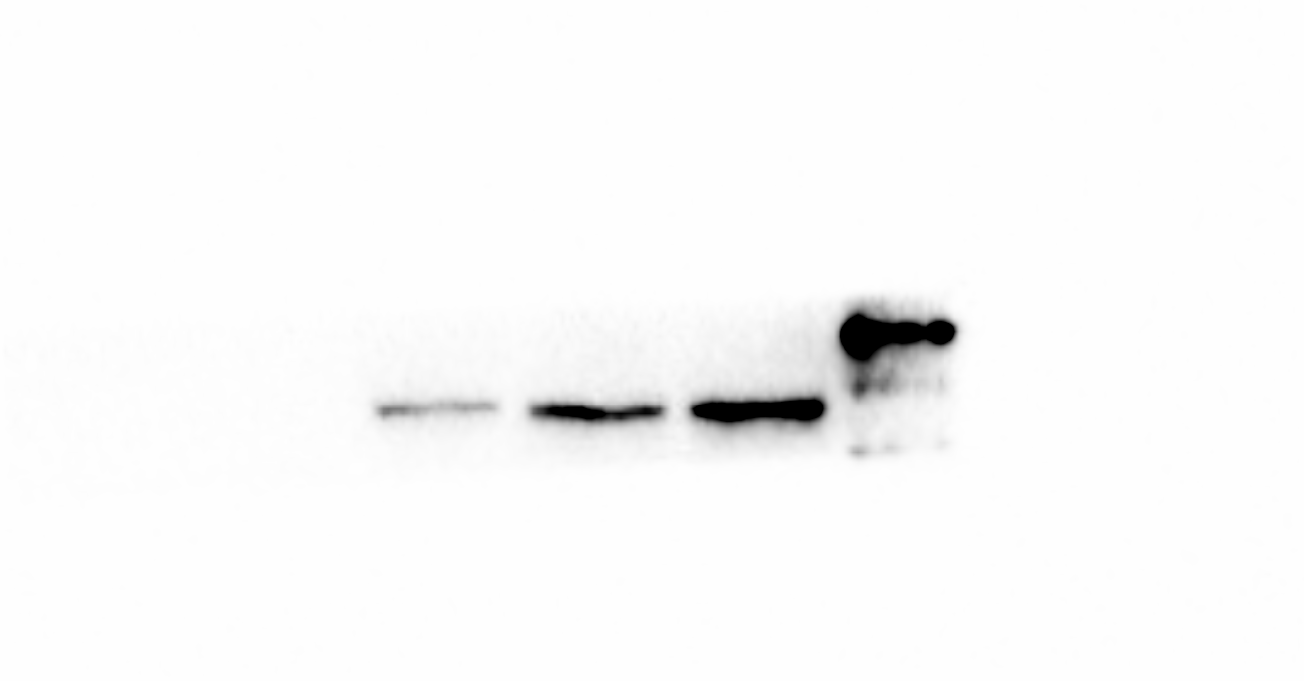

Supplement: Supplementary file 10 [file DataSheet7.zip › 3、WB bands from THP-1 cell gout model/JAK2-5.tif]

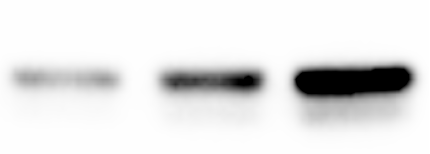

Supplement: Supplementary file 10 [file DataSheet7.zip › 3、WB bands from THP-1 cell gout model/STAT1-2.tif]

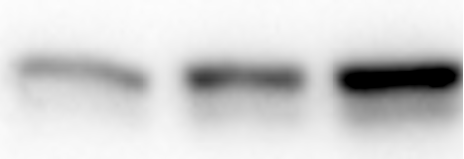

Supplement: Supplementary file 10 [file DataSheet7.zip › 3、WB bands from THP-1 cell gout model/STAT1-3.tif]

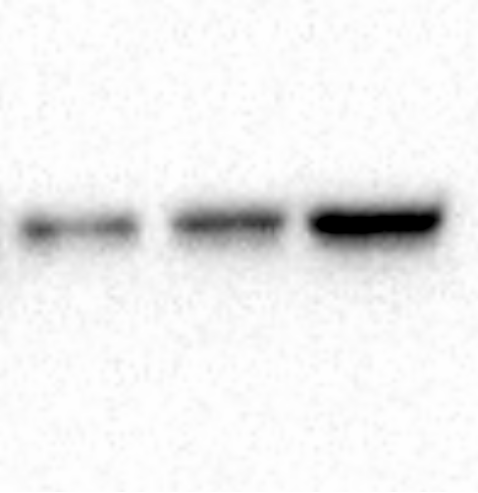

Supplement: Supplementary file 10 [file DataSheet7.zip › 3、WB bands from THP-1 cell gout model/STAT1-4.tif]

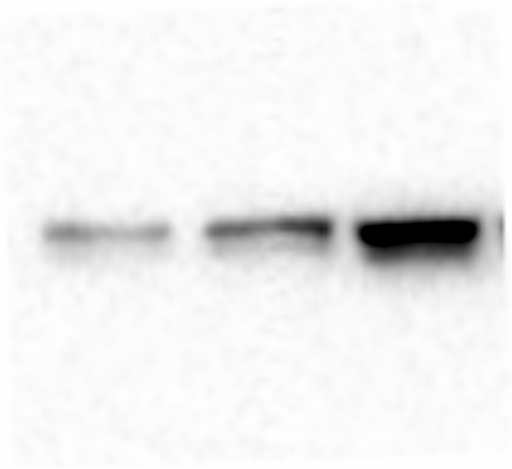

Supplement: Supplementary file 10 [file DataSheet7.zip › 3、WB bands from THP-1 cell gout model/STAT1-5.tif]

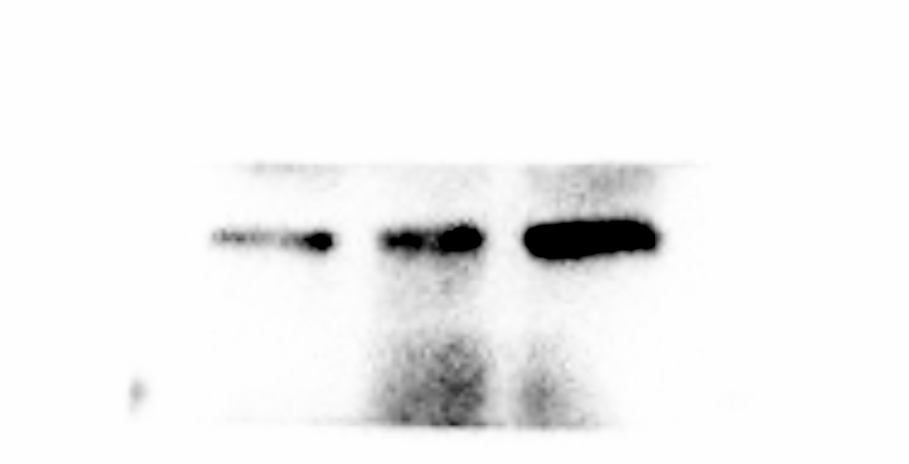

Supplement: Supplementary file 10 [file DataSheet7.zip › 3、WB bands from THP-1 cell gout model/STAT3-2.tif]

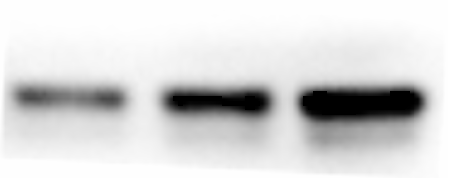

Supplement: Supplementary file 10 [file DataSheet7.zip › 3、WB bands from THP-1 cell gout model/STAT3-3.tif]

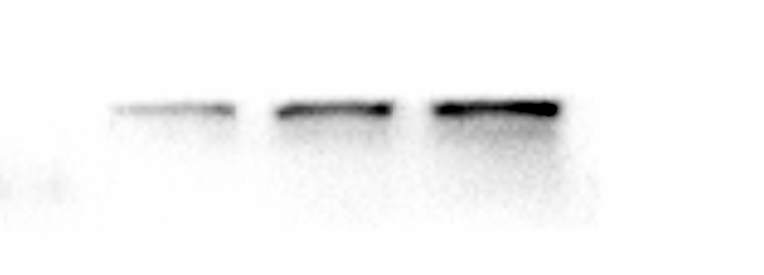

Supplement: Supplementary file 10 [file DataSheet7.zip › 3、WB bands from THP-1 cell gout model/STAT3-4.tif]

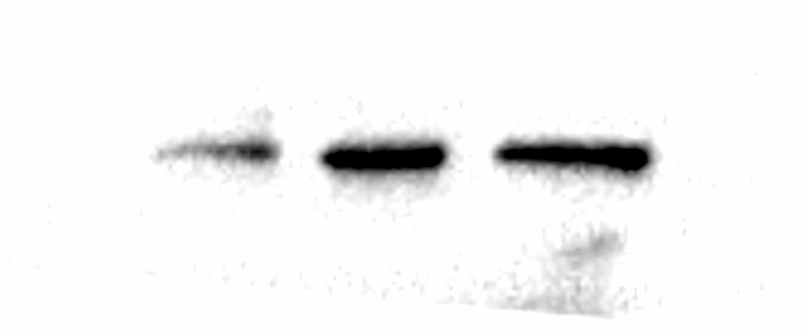

Supplement: Supplementary file 10 [file DataSheet7.zip › 3、WB bands from THP-1 cell gout model/p-JAK2-2.tif]

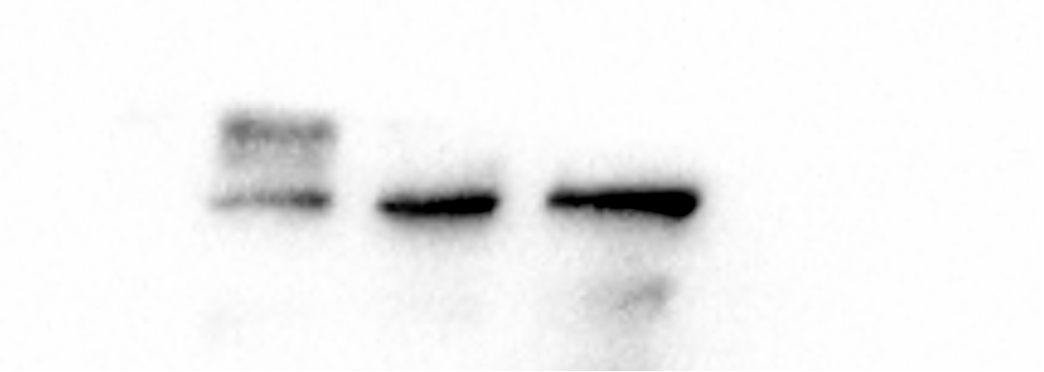

Supplement: Supplementary file 10 [file DataSheet7.zip › 3、WB bands from THP-1 cell gout model/p-JAK2-3.tif]

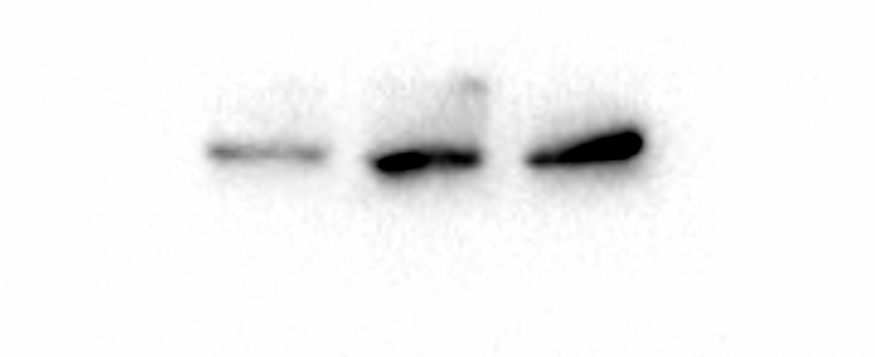

Supplement: Supplementary file 10 [file DataSheet7.zip › 3、WB bands from THP-1 cell gout model/p-JAK2-4.tif]

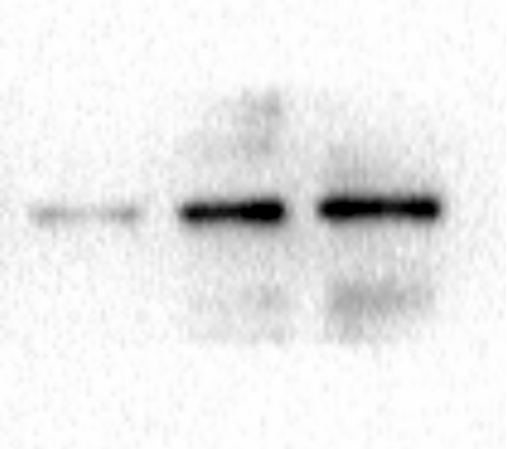

Supplement: Supplementary file 10 [file DataSheet7.zip › 3、WB bands from THP-1 cell gout model/p-JAK2-5.tif]

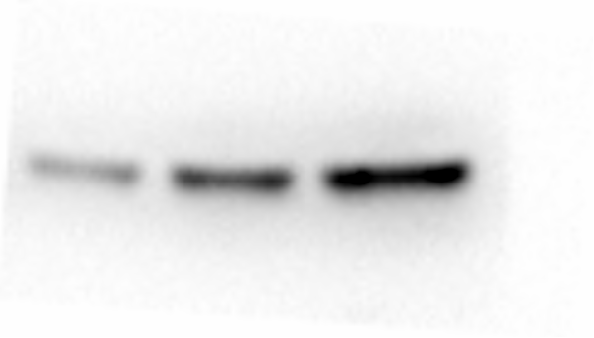

Supplement: Supplementary file 10 [file DataSheet7.zip › 3、WB bands from THP-1 cell gout model/p-STAT1-2.tif]

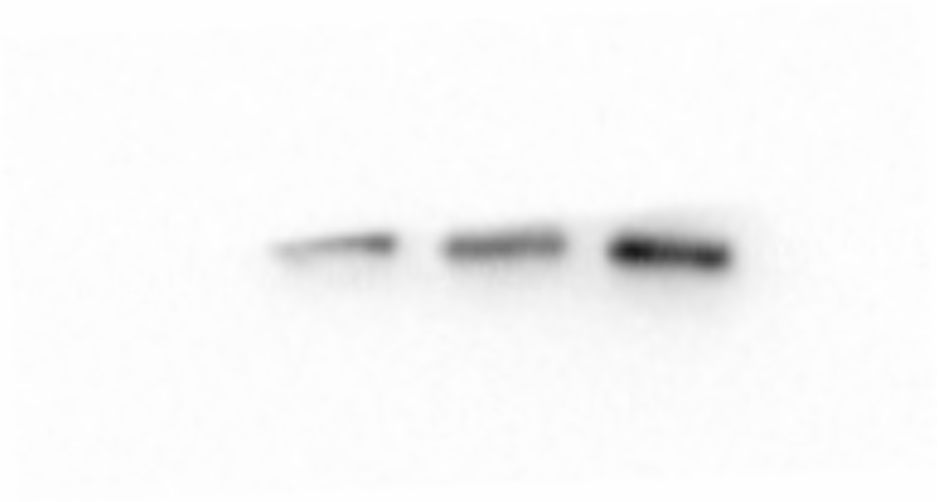

Supplement: Supplementary file 10 [file DataSheet7.zip › 3、WB bands from THP-1 cell gout model/p-STAT1-3.tif]

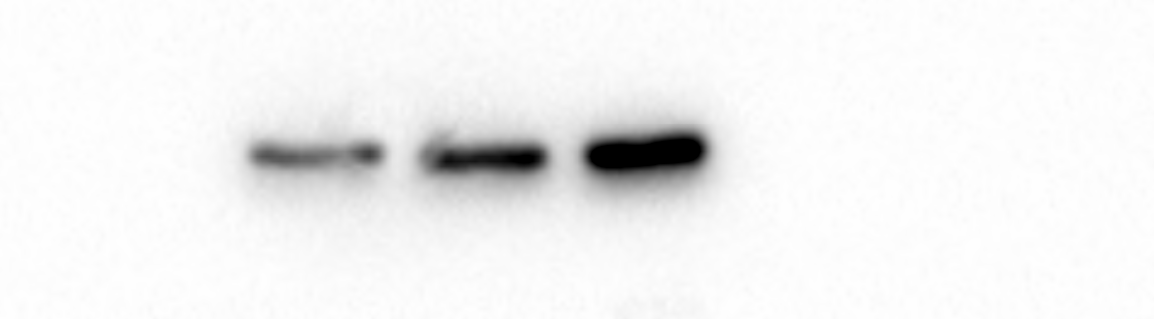

Supplement: Supplementary file 10 [file DataSheet7.zip › 3、WB bands from THP-1 cell gout model/p-STAT1-4.tif]

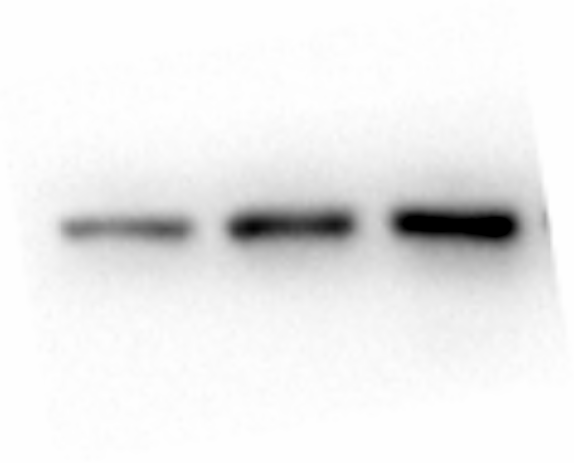

Supplement: Supplementary file 10 [file DataSheet7.zip › 3、WB bands from THP-1 cell gout model/p-STAT1-5.tif]

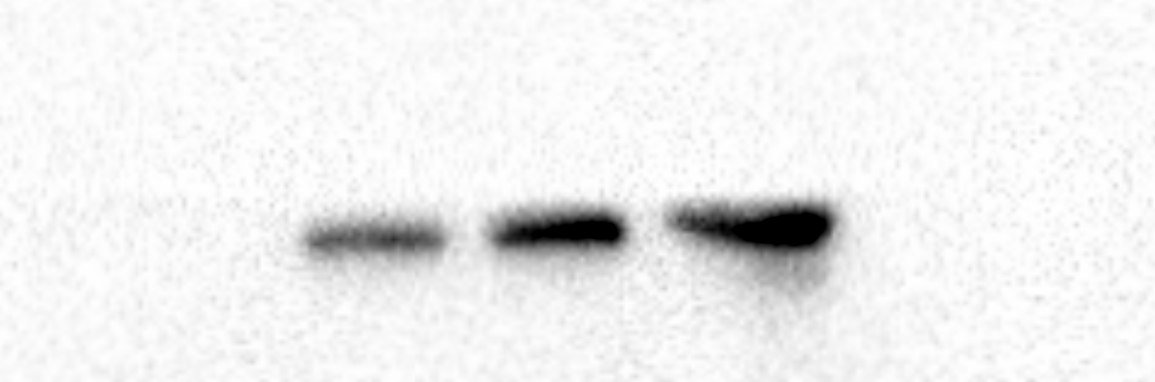

Supplement: Supplementary file 10 [file DataSheet7.zip › 3、WB bands from THP-1 cell gout model/p-STAT3-2.tif]

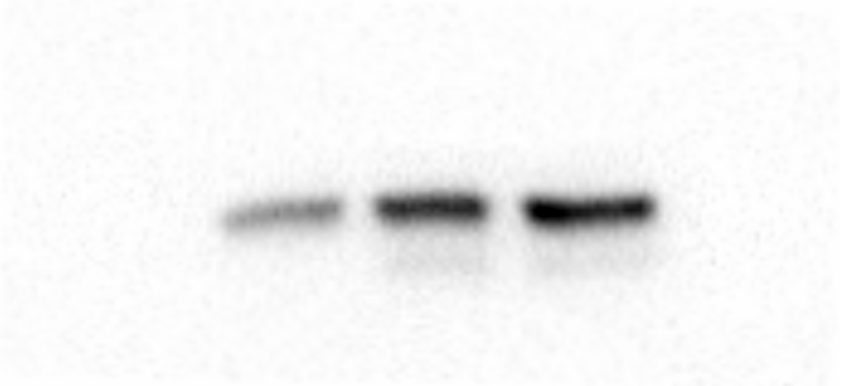

Supplement: Supplementary file 10 [file DataSheet7.zip › 3、WB bands from THP-1 cell gout model/p-STAT3-3.tif]

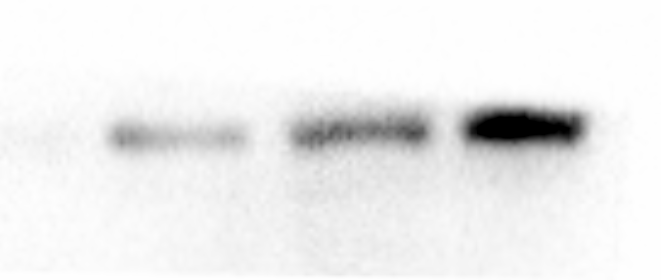

Supplement: Supplementary file 10 [file DataSheet7.zip › 3、WB bands from THP-1 cell gout model/p-STAT3-4.tif]
